# Occurrence and Co-Occurrence of Regulated and Emerging Mycotoxins in Foods Marketed to U.S. Toddlers

**DOI:** 10.3390/ijerph23080949

**Published:** 2026-07-23

**Authors:** Susan Gonya, Julie Brunkhorst, Michael Laviolette

**Affiliations:** 1 Clean Food Solutions Research Institute, East Kingston, NH 03827, USA; 2 Trilogy Analytical Laboratory, Washington, MO 63090, USA; julie@trilogylab.com; 3 Independent Researcher, Boston, MA 02115, USA

**Keywords:** mycotoxins, food safety, emerging mycotoxins, toddler foods, multi-toxin contamination, pediatric nutrition, risk assessment

## Abstract

**Highlights:**

**Public health relevance—How does this work relate to a public health issue?**

- Mycotoxin contamination in foods marketed to U.S. toddlers appears to be highly prevalent, indicating routine early-life exposure to foodborne toxicants with the potential to adversely affect child health.
- Early childhood is a critical window of vulnerability in which small body size and immature metabolic and immune systems may increase susceptibility to mycotoxin exposure arising from food production, storage conditions, and climate-related factors.

**Public health significance—Why is this work of significance to public health?**

- This study presents novel U.S.-based data on the occurrence and co-occurrence of 34 regulated and emerging mycotoxins in toddler foods, addressing a significant data gap in North America that has contributed to their under-recognition in public health sectors.
- The findings highlight systemic limitations of single-toxin regulatory frameworks, particularly in the context of complex, real-world exposures that may disproportionately impact vulnerable populations, such as young children.

**Public health implications—What are the key implications or messages for practitioners, policy makers, and/or researchers in public health?**

- Public health frameworks should integrate the assessment of multi-toxin exposures and environmental drivers into surveillance programs to enhance food safety and promote health equity.
- Policies and interventions should prioritize enhanced surveillance, updated regulatory standards, and child-specific food safety measures to reduce dietary mycotoxin exposure and improve equitable access to safer food systems.

**Abstract:**

Foodborne mycotoxins are toxic secondary metabolites produced by filamentous fungi. At sufficient concentrations, ingested mycotoxins have been found to disrupt the microbiome and exert toxic effects on gastrointestinal, hepatic, renal, and other tissues. Toddlers are particularly vulnerable to ill effects due to increased intake relative to lower body weight and immature detoxification, metabolic, and immune function. Despite this susceptibility, their adverse health effects remain under-recognized in public health sectors, and data on mycotoxin contamination in foods marketed to young children in North America remain sparse. To investigate this, 118 food products, including cereals, snacks, pasta, first foods, juices, and staple ingredients, were purchased at retail and analyzed for 34 mycotoxins using liquid chromatography–tandem mass spectrometry. The results were compared with the United States Food and Drug Administration (FDA) regulatory thresholds and European tolerable daily intake (TDI) limits. Of the 34 analytes, 32 were detected, and 25 were quantified. At least one mycotoxin was quantified in 87 percent of products, with a mean of 3.7 per item and a maximum of 13. Multi-toxin contamination was common; many products exceeded the FDA and TDI limits, and many remain unregulated. These findings highlight regulatory gaps in food safety and underscore the need for stronger child-focused oversight, particularly regarding emerging mycotoxins and multi-toxin contamination.

## 1. Introduction

Mycotoxins are low-molecular-weight secondary metabolites produced by filamentous fungi under favorable environmental and storage conditions [1,2]. Since the 1960s, hundreds of mycotoxins have been identified, with dietary ingestion being the primary route of human exposure [1]. Inhalation and dermal contact may also occur in mold-contaminated indoor environments, such as water-damaged buildings, and in occupational settings, including bakeries and breweries, making complete avoidance difficult [2,3,4].

Historically, outbreaks of mycotoxin-related foodborne illnesses were often regionally confined before the expansion of global trade [2,5]. However, mycotoxin contamination is now widespread, with an estimated 60–80% of cereal grains and nut crops affected worldwide, posing risks to both human and animal health [1]. As a result, humans are continuously exposed to low and, at times, high levels of dietary mycotoxins, with infants and young children among the most disproportionately affected due to their lower body weight and higher relative toxin exposure [6,7,8].

The primary mycotoxin-producing genera affecting the human food supply include *Aspergillus*, *Penicillium*, *Fusarium*, and *Alternaria* species [2]. These fungi produce a chemically diverse range of toxic compounds, including aflatoxins, ochratoxins, trichothecenes (e.g., deoxynivalenol, nivalenol, HT-2 toxin, and T-2 toxin), zearalenone, fumonisins, enniatins, beauvericin, alternariol monomethyl ether, and alternariol [2]. Many of these metabolites have been shown to adversely affect multiple organ systems, including the gastrointestinal tract, liver, kidneys, nervous system, reproductive organs, and epithelial and immune tissues [3,4,9,10].

Within the context of organ-specific toxicities, many mycotoxins have acute and chronic health effects. For example, at low but chronic doses, the commonly occurring trichothecene mycotoxin deoxynivalenol (DON) may stimulate immune activation, inhibit protein synthesis, compromise the intestinal mucus barrier, increase intestinal permeability, and suppress appetite, thereby potentially contributing to growth stunting in children [11,12,13,14,15,16,17,18]. Higher, more acute exposures to DON have been linked to immune suppression, nausea, vomiting, abdominal pain, diarrhea, intestinal hemorrhage, and fever in both animal models and human outbreaks [5,9,11,12,13,16,19,20,21]. These effects have led some researchers to hypothesize that chronic trichothecene ingestion may contribute to inflammatory GI disorders, including inflammatory bowel disease (IBD) [3,13,19,20,21].

Some mycotoxins have been found to cross the blood–brain barrier following their systemic absorption. DON and ochratoxin A (OTA) can cross the blood–brain barrier and have been shown to damage astrocytes and glial cells in animal and in vitro models [15,22]. Detoxification differences between developmentally typical children and their siblings with autism spectrum disorder (ASD) have raised concerns about their potential role in ASD [14,15,22,23,24,25].

Other mycotoxins also exhibit distinct organ-specific effects. Cyclopiazonic acid, an unregulated mycotoxin, has been linked to gastrointestinal (GI) and neurological toxicity, including human Kodua poisoning [26,27,28,29,30]. Aflatoxins are well-established hepatotoxins and hepatocarcinogens, while enniatins and *Alternaria* toxins, including alternariol (AOH) and alternariol monomethyl ether (AME), may also contribute to liver pathology, although their roles remain less well-defined [31,32,33,34,35,36,37,38]. Zearalenone (ZEA), AOH, and AME exhibit hormone-disruptive activity, and ZEA has been associated with reproductive abnormalities, including polycystic ovarian syndrome in animal models [2,37,39,40,41,42,43,44]. Fumonisin exposure has been implicated in the development of neural tube defects in infants and possibly esophageal cancer in humans [2,45,46]. Aflatoxin B1, fumonisins, and DON have recently been under investigation for their potential role in growth stunting in children [47,48].

In addition to direct toxicity, many mycotoxins disrupt intestinal microbial communities in a manner similar to that of antibiotics. DON alters the relative abundance of *Bacteroides* and *Firmicutes*, resembling microbial shifts reported in IBD and ASD [49,50,51,52,53]. Aflatoxins reduce the number of lactic acid-producing bacteria, and OTA targets lactobacilli, including *Lactobacillus reuteri* [54]. Beauvericin exhibits broad antimicrobial activity against Gram-positive and Gram-negative organisms, potentially contributing to dysbiosis [55].

Collectively, these findings highlight the potential negative impact of foodborne mycotoxins on the human microbiome and overall health. Despite decades of research and the identification of many foodborne mycotoxins with known toxicity, numerous fungal metabolites remain under-recognized in clinical and public health fields [9,30,56,57].

Exposure can start in utero, as mycotoxins have been found in amniotic fluid and are linked to impaired fetal growth [58]. In a prospective cohort study, Tan et al. [59] reported that mothers in the highest tertile of DON intake were more likely to deliver infants with lower birth weights and reduced birth lengths relative to gestational age (all *p*-trends < 0.05). Postnatally, exposure may continue through contaminated breast milk, infant formula, and cow’s milk, with additional increases as solid foods are introduced [6,8,59,60,61,62].

**Figure 1:**
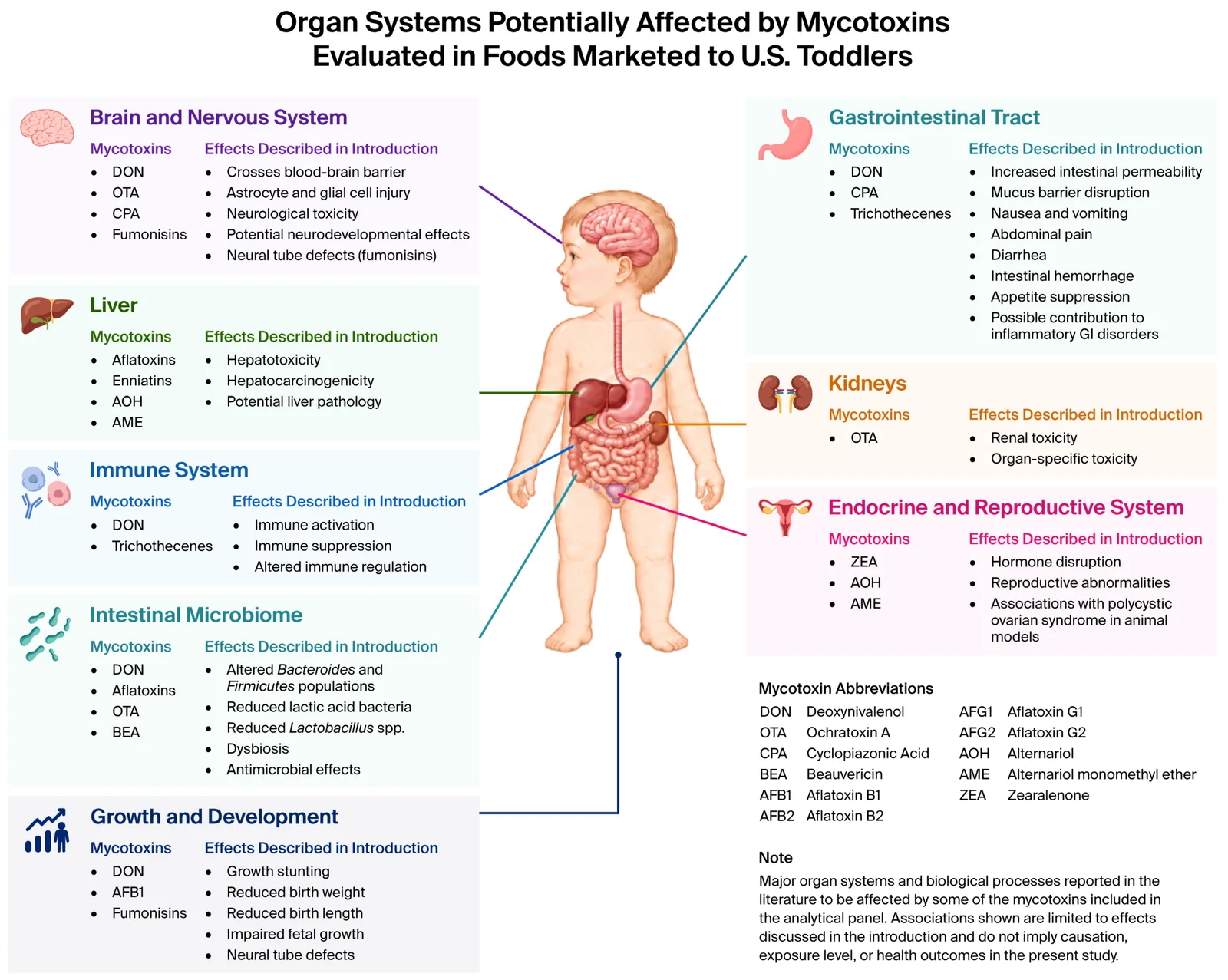
Organ systems potentially affected by mycotoxins.

Following weaning, toddlers consume a broader range of solid foods, many of which are grain-based products, such as cereals, crackers, breads, and snack foods. Grains are common sources of mycotoxins and are consumed more frequently relative to body size; this transition may further increase exposure compared to that in infancy [7,63]. These risks may be amplified by developmental vulnerabilities, including immature and less resilient microbiomes, underdeveloped metabolic and immune systems, reduced detoxification capacity, and lower body weight [8,64]. Figure 1 provides an overview of the major organ systems and biological processes reported in the literature for the mycotoxins summarized herein.

Compounding this concern, mycotoxins are imperceptible to humans by taste, odor, or visual inspection, rendering avoidance difficult without advanced analytical methods, such as liquid chromatography–tandem mass spectrometry (LC-MS/MS) [65]. Historically, most surveillance efforts have focused on a limited subset of regulated mycotoxins, partly because of the methods and standard materials available for quantification. The primary mycotoxin detection methods were High-Performance Liquid Chromatography with Fluorescence Detection (HPLC-FLD), High-Performance Liquid Chromatography with Ultraviolet Detection (HPLC-UV), and Gas Chromatography (GC). These methods can primarily identify single mycotoxins or groups of mycotoxins, but not to the extent that LC-MS/MS allows. Moreover, with more analytical standards becoming available each year, this instrumentation has evolved to allow the quantification of previously undetected and emerging mycotoxins. Libraries of hundreds of mycotoxins have been produced, enabling screening using LC-MS/MS and other time-of-flight instruments [66]. Thus, previous technological limitations may explain why only a small number of mycotoxins are currently subject to regulatory limits in the United States (U.S.). Currently, only limited data are available on the occurrence and co-occurrence of regulated and emerging mycotoxins in the U.S. food supply.

To address these gaps, the present study evaluated the occurrence and co-occurrence of 34 regulated and emerging mycotoxins in processed foods marketed for U.S. toddlers, providing a snapshot of potential early life dietary exposure. Specifically, this investigation seeks to address the following questions: (1) which of the 34 mycotoxins tested U.S. toddlers are likely to be exposed to through commonly available processed foods, and (2) whether observed contamination levels exceed established European tolerable daily intake (TDI) values when adjusted for toddler body weight, thereby potentially increasing risks to child health. European TDI values were used where available, as no established pediatric TDI values are available in the U.S.

## 2. Materials and Methods

**Figure 2:**
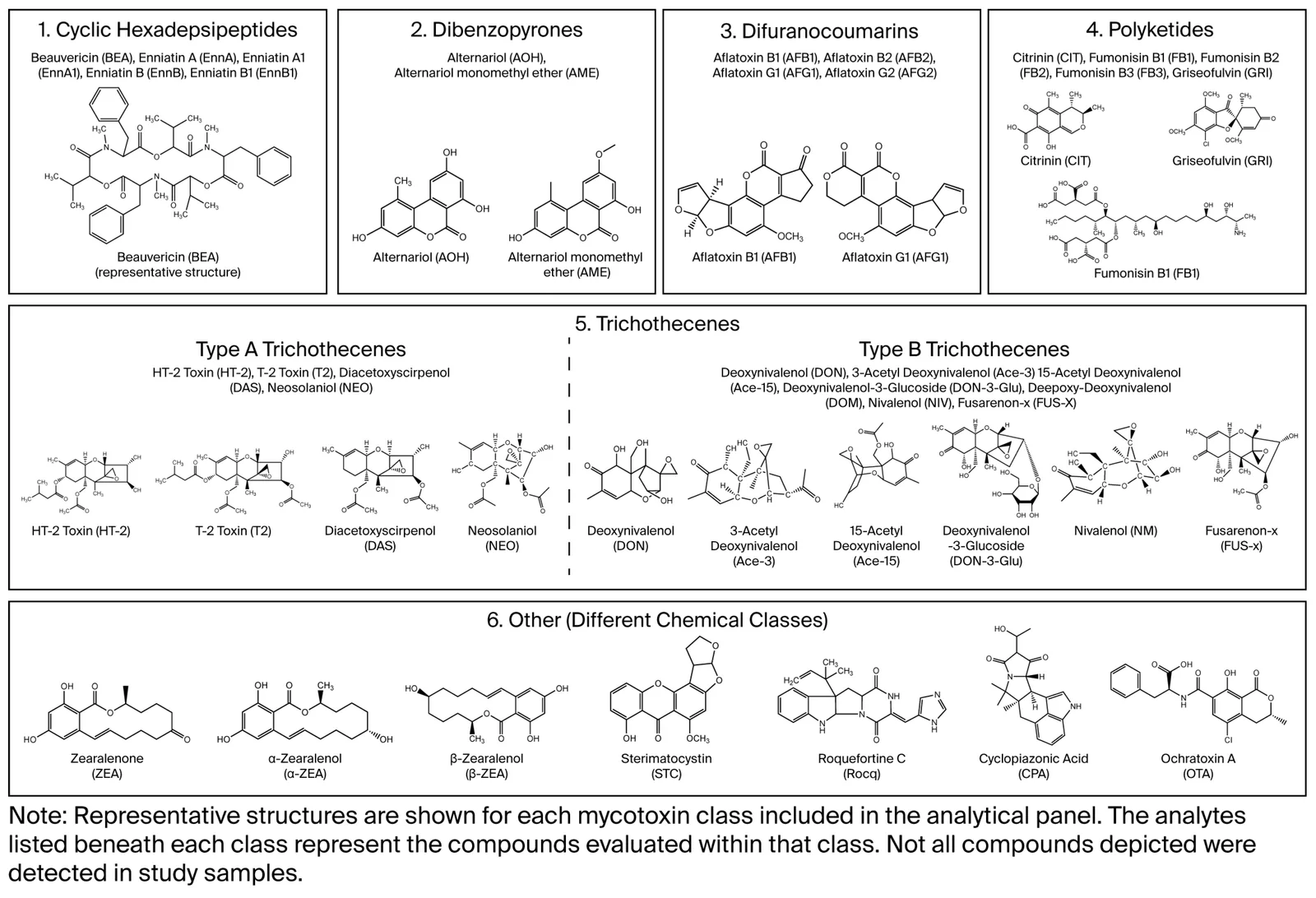
Representative chemical structures of analytical panel mycotoxin classes.

A total of 118 food products were screened for 34 mycotoxins using LC–MS/MS to assess multi-mycotoxin contamination. Figure 2 shows the representative structures of each mycotoxin class included in the analytical panel. The analytes pictured beneath each structure represent the compounds evaluated within that chemical class (see Appendix A for additional information on the analytes tested). A validated multi-analyte method was applied for sample preparation and quantification, as described by Kresse et al. [65]. Quality control measures were implemented throughout the analytical process.

### 2.1. Sample Selection and Procurement

Food products specifically marketed for toddler consumption, including cereals, snacks, pasta products, first foods, juices, and selected cooking ingredients commonly used in foods prepared for young children, were selected for analysis. Toddlers were defined as children aged at least 12 months but less than 36 months. Only vegetable-based, non-dairy items were included, as defined a priori by the study team. Cereal grain-based foods and some non-cereal grain ingredients were selected for testing. Non-cereal-based ingredients included chocolate powder, soy-based formula, almond flour, coconut flour, arrowroot, tapioca starch, and other miscellaneous products (e.g., first food beans and apple juice).

Products were purchased at retail locations and by mail order in the Midwestern United States (Missouri) and Northeastern United States (Massachusetts, New Hampshire, and Maine) between 1 June 2024 and 1 October 2025. In the absence of a formal randomization protocol, a standardized systematic sampling approach was implemented to reduce selection bias when purchasing directly from individual stores. Specifically, the second item in each product row was selected, provided that its packaging was intact. Additionally, only one product was selected from each product category (e.g., cornflakes) on the shelf, regardless of the brand. However, because two separate persons procured the food items, duplicate products from different geographic regions and lots were sometimes selected.

To supplement retail sampling and expand geographic coverage, a mail-order procurement strategy was also implemented to obtain commercially available food products for mycotoxin analysis. Products marketed for infant and toddler consumption, along with some basic cooking ingredients, were identified through major online retailers and manufacturers’ websites via Google search and purchased using a standardized selection approach. Within each product category, one item from every other available brand was selected to reduce selection bias.

Prior to laboratory submission, all products were inspected to confirm package integrity, the absence of visible damage (e.g., rips or tears), and the validity of expiration dates to ensure sample quality. Details for each product were also noted, including manufacturer lot numbers and storage facility numbers, when available. However, such details were not available for all the products procured.

### 2.2. Sample Shipment and Laboratory Analysis

After procurement, samples were sent via U.S. Postal Service Priority Mail to Trilogy Analytical Laboratory (Washington, MO, USA) for the analysis of 34 foodborne mycotoxins using LC-MS/MS procedures. Once received by the laboratory, samples were stored in their original, unopened packaging in a dehumidified refrigeration unit. This was carried out to minimize potential contamination or degradation prior to analysis. All samples were logged with the receipt date, product type, brand, lot number (if available), and country of origin, if disclosed on the label. Samples were stored for no more than two weeks in their original sealed packaging and were not opened before extraction and analysis. Prior to sampling, the products were inspected for damage and visible signs of mold and discarded if found damaged. Samples were tested in groups by matrix (e.g., packaged cereals). Each sample was ground and homogenized using a Retsch GM200 knife mill (Retch GMBH, Dusseldorf, Germany) with serrated blades for approximately one minute at 10,000 min^−1^. They were then weighed (25 g) into 250 mL Erlenmeyer flasks, and 100 mL of 80% acetonitrile/water was added. The flasks were placed on a platform shaker (Eberbach, Van Buren Charter Township, MI, USA) and shaken for 1.5 h. Any remaining samples were placed in Ziploc bags and stored under refrigeration.

### 2.3. Reagents and Standards

To test for 34 mycotoxins, reference standards for all targeted mycotoxins (≥98% purity) were manufactured by Trilogy Analytical Laboratory (Washington, MO, USA) or purchased from Romer Labs (Union, MO, USA). Stock solutions were prepared at a concentration of 1 mg/mL. These stock standards were then prepared at working standard concentrations (µg/mL) for use in the analysis in LC–MS-grade acetonitrile, methanol, and water. LC–MS-grade acetonitrile, methanol, and water were obtained from Honeywell. Formic acid (≥99% purity, LC–MS-grade) was purchased from Millipore Sigma (Burlington, MA, USA).

### 2.4. Sample Preparation

Diverse food subsets require varying methods of preparation. For products prone to heterogeneity (e.g., cereals and mixed snacks), incremental portions were taken from multiple locations within the homogenized matrix to ensure representativeness. Liquid products were mixed thoroughly prior to aliquoting.

Samples were then prepared for testing using the following methods: Samples were ground using a Retsch GM200 mill or homogenized using a high-speed blender (Osterizer, Sunbeam Products Inc., Atlanta, GA, USA) until a uniform particle size was achieved. Then, 25 g samples were weighed in an extraction flask, and 100 mL of acetonitrile–water (80:20, *v/v*) was added.

Extraction was performed on an orbital shaker (6000, Eberbach) for 90 min at room temperature (20–22 °C). The samples were then filtered using a Whatman No. 1 filter (Jade Scientific, Westland, MI, USA). The filtered extract was purified using QP1100 SPE (Trilogy Analytical Laboratory, Washington, MO, USA). The purified extracts were diluted with water–acetic acid (2% *v/v*) and injected into the LC-MS/MS system.

### 2.5. LC–MS/MS Instrumentation and Parameters

For LC-MS/MS analysis, the Shimadzu SIL-40 series system (Columbia, MD, USA) was configured with a near-zero refrigerated autosampler (temperature maintained at 8 °C), gradient pump, and column oven. This was combined with the SCIEX 7500 mass spectrometry system using an Electrospray Ionization Interface (ESI). The preinstalled 6-port valve on the MS-MS instrument was configured to direct the analyte flow to the waste. The SCIEX OS acquisition software (Version 4.0) was used to control all systems, and the SCIEX OS software was used to evaluate the data [67].

#### 2.5.1. Chromatographic Separation

Separation was performed using a Phenomenex Kinetex column (3 mm × 100 mm; 2.6 μm; 100 Å) (Torrance, CA, USA), which was then stored in a column oven. The temperature was set to 40 °C, and the injection volume was 5 μL.

The mobile phases for the analysis were A: water–0.1% Formic Acid–4 mM Ammonium Formate, and B: Methanol–0.1% Formic Acid–4 mM Ammonium Formate. The flow rate was 0.5 mL/min.

A gradient program was used for separation. The gradient profile started at 5% B, was held for 1.0 min, and then increased linearly to 50% B within 6.0 min. This was maintained for 12.0 min before being returned to 5% B in 4.0 min. The starting conditions were maintained for 4.0 min before the next injection.

#### 2.5.2. Mass Spectrometry Conditions

The parameters for MS/MS were as follows: ESI positive with an interface temperature of 350 °C, an ion spray voltage of 1700 V, curtain gas at 40 psi, collision gas at 9 psi, ion source gas 1 set at 40 psi, and gas 2 set at 60 psi. Mass spectrometry was performed using scheduled multiple reaction monitoring (sMRM), and the two most intensive transitions were measured when the relevant substance eluted to the MS/MS. The dwell times were optimized automatically by Sciex OS Software.

Analyses were performed in scheduled multiple reaction monitoring (sMRM) mode. For each analyte, the two most intense precursor–product ion transitions were monitored within a 1.2 min retention window. The dwell times were automatically optimized using Sciex OS software (4.0, Sciex) (Marlboro, MA, USA) [67] to ensure adequate data points across each peak. The window of one MSM signal was 1.2 min, and the total cycle time was 1.1 s [67].

The analytes required both positive and negative ionization polarities. The complete MS/MS operating settings are provided in Table 1 [65].

#### 2.5.3. Method Validation

The method’s performance was evaluated in accordance with FDA guidelines. Linearity was assessed using matrix-matched calibration curves prepared in triplicate across a range. The coefficients of determination (R^2^) exceeded 0.99 for all analytes (see the Supplementary Materials—supplemental verification data).

The limits of detection (LOD) and quantification (LOQ) were determined using signal-to-noise ratios of 3:1 and 10:1, respectively, in spiked blank matrices (samples previously analyzed and containing no mycotoxins). The recoveries were assessed by fortifying additional blank matrices. The mean recoveries ranged from 70% to 120%, with relative standard deviations (RSD) ≤15%. Matrix effects were evaluated by comparing the slopes of calibration curves prepared in solvent versus matrix extracts. If mycotoxins were detected but were below the typical LOQ, the data were considered non-quantifiable and discarded from the overall statistical dataset. The obtained LOQs were below the regulatory limits for mycotoxins in cereal-based products, as set in Commission Regulation 2023/915, whereas the more stringent limits for grain-based baby food were in the range of, or slightly below, the respective LOQs for aflatoxins [68].

### 2.6. Statistical Analysis and Comparison to Established Tolerable Intake Levels

For each sample, data were entered into a Microsoft Excel (Version 2601 Build 16.0.19628.20132) spreadsheet and evaluated using descriptive statistics [69]. The data were then converted to micrograms per kilogram (µg/kg) for ease of clinical interpretation and comparison with the TDI and current regulatory guidance levels. The mean number of samples in which at least one mycotoxin was detected was calculated for each tested category. R statistical software (version 4.5.2) running under RStudio (version 2026.06.0 build 242) [70] and add-on packages were used to further evaluate the data [71,72,73,74]. A simulated Pearson’s chi-squared test was performed to determine the distribution of mycotoxins across all groups.

The median and maximum detection levels were computed for mycotoxin contamination levels, and the mean rates were calculated for the number of positive samples. The median detection levels, rather than mean values, for mycotoxin contamination were chosen because the median more accurately represents the central tendency when contamination data are highly variable, skewed, and influenced by extreme values. These are typical characteristics of mycotoxin datasets, especially when many analytes are measured simultaneously. The median is robust to outliers and therefore provides a more stable, representative estimate of the “typical” contamination level in the food supply.

The maximum and median detection levels were then evaluated against body weight (bw) to assess pediatric risk using pediatric portion sizes and with current TDI recommendations using an algorithm [75]. This equation was developed by the Italian National Institute of Health, Department of Innovation in Biology, at the University of Tuscia, Italy, and an Italian food company to produce processed wheat-based products with child-safe DON levels [75]. This equation provides a method for comparing the median and maximum mycotoxin levels in food samples with established TDIs.

Exposure ng/kg bw/day = Mycotoxin Contamination level (µg/kg) × Consumption data (g)/bw (kg)

For consistency, the mean toddler weights-for-age for a 24-month-old were obtained from the Centers for Disease Control and Prevention (CDC) growth charts. The 50th percentile for girls and boys combined (12.5 kg) was input into the equation [76].

Representative serving sizes for toddlers were derived from the nationally representative dietary intake data reported in the Feeding Infants and Toddlers Study (FITS) 2016 and the What We Eat in America/National Health and Nutrition Examination Survey (WWEIA/NHANES). USDA Food Pattern recommendations were used to verify the age-appropriate portion sizes. Serving sizes were selected to reflect typical consumption by children approximately 24 months of age [77,78].

Estimated mycotoxin intakes were initially calculated in nanograms and subsequently converted to micrograms by dividing by 1000. Final exposure estimates are expressed as µg/kg bw/day and compared with the corresponding tolerable daily intake (TDI) values for each mycotoxin. TDI limits have not been established for all the mycotoxins tested. When TDI values were unavailable, the Threshold of Toxicological Concern (TTC) was used to evaluate whether food levels may represent hazard flags warranting further investigation. Given the uncertainty in TTC values, this was done only for screening purposes.

Microsoft Excel was used to evaluate whether toddlers exceeded the TDI and TTC exposure limits using the following equation [69]:

Toddler Exposure = TDI (µg/kg bw/day)/Actual intake (µg/kg bw/day)

This method allows for the easy comparison of mycotoxin exposure levels in food with established TDI limits. Pediatric exposure estimates exceeding established TDI values were considered potential hazard flags, indicating the need for further risk evaluation, particularly given the increased dietary intake per unit of body weight among young children. Quantifiable maximum and median levels were also compared with the FDA, Chinese, and European regulatory levels for small children. See Appendix B for the regulatory guidance levels.

## 3. Results

One hundred and eighteen (118) processed foods and ingredients were analyzed. A broad range of foods was sourced for testing, including snack foods, first foods and formulas, packaged cereals, and pasta (*n* = 72 samples), as well as raw ingredients used in food preparation (*n* = 46 samples). Contamination rates differed across commodities. The categories of food samples tested are shown in Table 2.

Among the 118 samples of food and basic ingredients analyzed, 112 samples (95%) reached the limit of detection (LOD) for at least one mycotoxin, and 103 samples reached the limit of quantification (LOQ). In the quantifiable sample set, 87% (95% CI = (81.1, 92.8)) of the samples contained at least one mycotoxin. The median number of quantifiable mycotoxins was 4.0, with a mean of 3.7 and a maximum of 13. Overall, a total of 4012 analyte evaluations were conducted (118 samples × 34 toxins), 706 (17.6%) reached the LOD for at least one mycotoxin, and 428 (10.7%) reached the LOQ.

Of the 34 mycotoxins examined, 32 were detected, with 25 quantifiable at varying concentrations across the sample set. The following mycotoxins were detected in trace amounts but not quantifiable beyond a detection signal: Roquefortine C, Griseofulvin, Aflatoxin G2, Neosolaniol, Citrinin, α-Zearalenol, and β-Zearalenol. Additional information on detected but non-quantifiable mycotoxins is provided in Appendix A. Diacetoxyscirpenol and Fusarenon-X were not detected in any product. Mycotoxins that could not be quantified were treated as dichotomous variables and excluded from further statistical evaluation owing to the uncertainty and imprecision associated with their numerical values.

**Figure 3:**
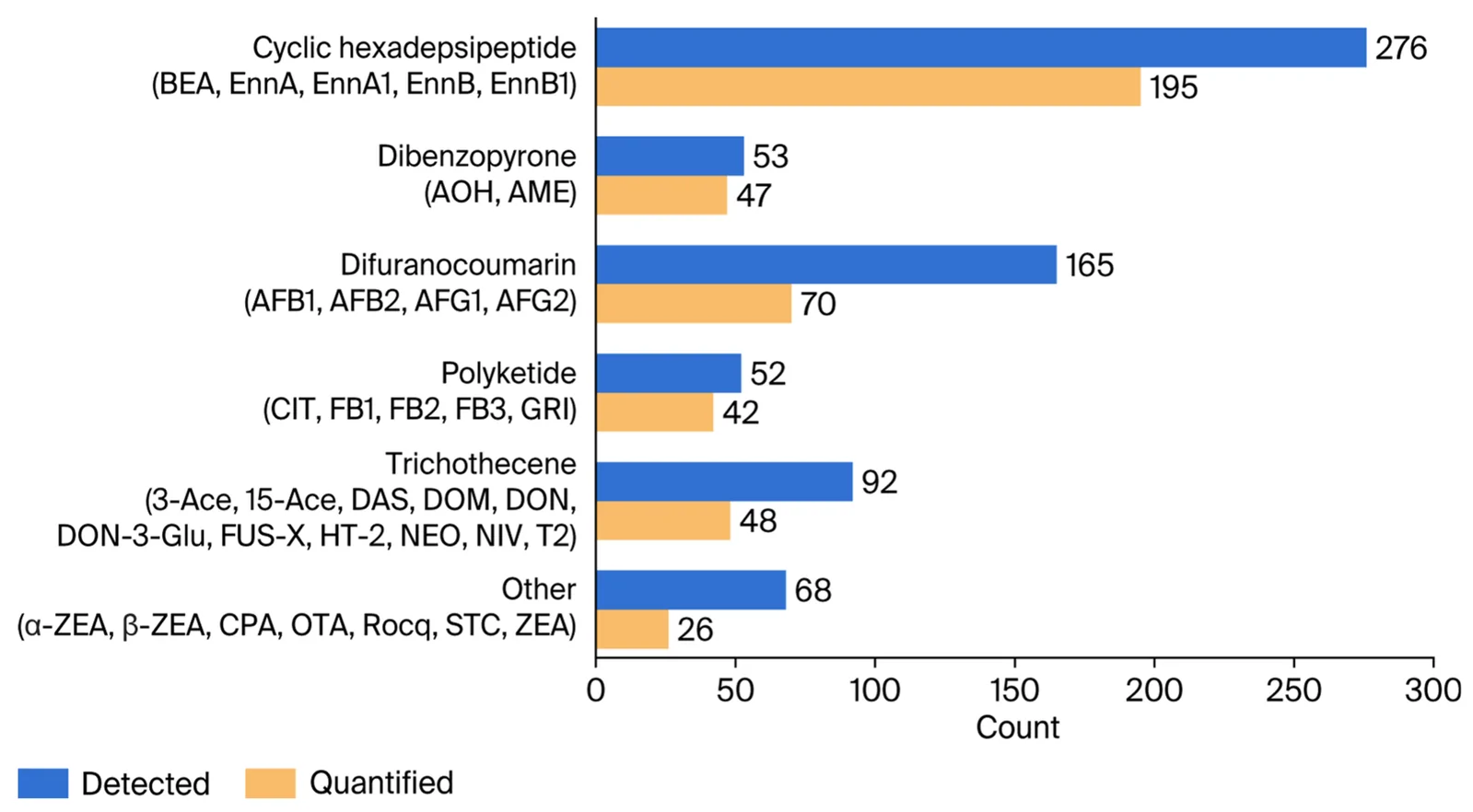
Numbers of detected and quantified mycotoxins by chemical structure. Notes: Cyclic hexadepsipeptides include beauvericin (BEA), enniatin A (EnnA), enniatin A1 (EnnA1), enniatin B (EnnB), and enniatin B1 (EnnB1). Dibenzopyrones include alternariol (AOH) and alternariol monomethyl ether (AME). Difuranocoumarins include aflatoxin B1 (AFB1), aflatoxin B2 (AFB2), aflatoxin G1 (AFG1), and aflatoxin G2 (AFG2). Polyketides include citrinin (CIT), fumonisin B1 (FB1), fumonisin B2 (FB2), fumonisin B3 (FB3), and griseofulvin (GRI). Trichothecenes include the following: Type A: HT-2 toxin (HT-2), T-2 toxin (T2), diacetoxyscirpenol (DAS), and neosolaniol (NEO); Type B: Deoxynivalenol (DON), 3-acetyl deoxynivalenol (Ace-3), 15-acetyl deoxynivalenol (Ace-15), deoxynivalenol-3-glucoside (DON-3-Glu), deepoxy-deoxynivalenol (DOM), nivalenol (NIV), and fusarenon-x (FUS-X); Other includes zearalenone (ZEA); α-zearalenone (α-ZEA) and β-zearalenone (β-ZEA); sterigmatocystin (STC); Rocquefortine C (Rocq); cyclopiazonic acid (CPA); and ochratoxin A (OTA).

A simulated Pearson’s chi-squared test indicated that the proportions of products with at least one quantifiable mycotoxin were approximately equal across all food types (*p* = 0.66). However, the types of mycotoxins found differed by commodity. Figure 3 presents the distributions of detected and quantified mycotoxins in foods, categorized by chemical structure.

Table 3 presents the number of samples contaminated within each mycotoxin category, the LOD and LOQ in micrograms per kilogram (µg/kg), the highest and median amounts found for each mycotoxin, and the type of food in which the highest amount was detected.

Table 4 shows the distribution of mycotoxins by chemical structure across food types. Cyclic hexadepsipeptides (e.g., ENNs and BEA) were the most abundant across all food categories, followed by difuranocoumarins (e.g., aflatoxins) and trichothecene mycotoxins (e.g., DON, T-2, and HT-2). Because the levels and types of mycotoxins detected varied by commodity, the results are broken down into food category subgroups for ease of interpretation.

**Figure 4:**
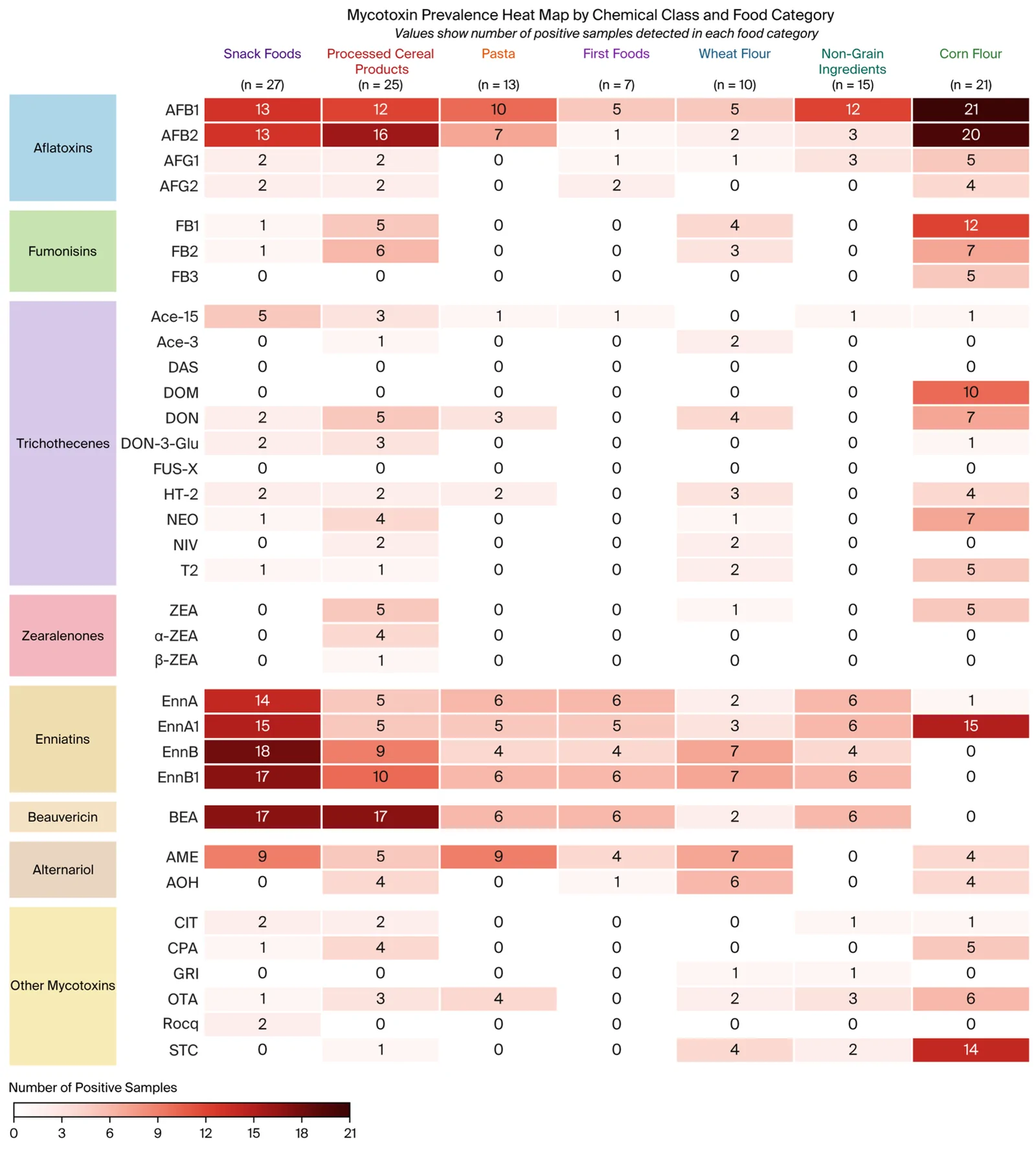
Heat map of detected mycotoxins by food category. Note: The values represent the number of samples in which each mycotoxin was detected within each food category. *n* = total number of samples analyzed for each food category. Sample sizes: Snack Foods (*n* = 27), Processed Cereal Products (*n* = 25), Pasta (*n* = 13), First Foods (*n* = 7), Wheat Flour (*n* = 10), Non-Grain Ingredients (*n* = 15), Corn Flour (*n* = 21).

Figure 4 presents a heat map illustrating the most frequently detected mycotoxins across various food categories. Notably, only aflatoxins, DON, and fumonisins are subject to regulation in the U.S.

### 3.1. Snack Foods (n = 27)

Eighty-five percent of the snack foods, which were all grain-based, were contaminated with at least one mycotoxin. The mean number of mycotoxins per sample was 4, with a maximum of 6. Figure 4 illustrates that the most prevalent co-occurrences of mycotoxins were ENNs, BEA, AME, and AFB. Among the quantifiable samples, 59% were contaminated with at least one ENN mycotoxin, 56% with BEA, 33% with AME, and 26% with AFB1. Furthermore, five of the 27 snack samples were contaminated with either type A or type B trichothecene mycotoxins. The trichothecene mycotoxins identified in these samples comprised 15-Ace, DON, T-2, and HT-2 toxins. Lastly, FB1, FB2, and CPA were detected together in a single corn-based vegetable stick snack.

### 3.2. Toddler First Foods (n = 7)

The toddler’s first food category consisted of three soy-based formulas, legumes, fruit puree, and fruit juice. Six samples contained at least two mycotoxins (86%), with a maximum of 8. Similar to snack foods, AFB1, the ENNs, BEA, and AME most frequently co-occurred in these samples. Apple juice marketed for infants and toddlers contained the highest levels of the emerging mycotoxins EnnA1, EnnA, and EnnB1 recorded in this study (100.7 ppb, 95.6 ppb, and 82.5 ppb, respectively). Eighty-six percent contained at least one ENN, 71% had detectable levels of AFB1, and 57% contained the emerging mycotoxin AME. One fruit puree sample contained both AOH and AME.

### 3.3. Processed Cereal Products (n = 25)

At least one mycotoxin was present and quantified in 84% of processed, packaged cereals. Moreover, packaged cereals were often contaminated with multiple mycotoxins from various mold species, with a mean of 3 and a maximum of 7. Co-occurrence was frequently observed among AFB1, AFB2, ENNs, BEA, and trichothecene mycotoxins (e.g., 21 detections). ZEA, DON, CPA, and BEA were detected in a single cereal sample.

In this category, 28% of the samples were contaminated with at least one quantifiable ENN, while 60% contained BEA. Aflatoxins were also detected in cereals, albeit at very low concentrations, significantly below the FDA limit of 20 μg/kg. CPA was identified in 16% of the samples, all of which were derived from corn. Additionally, 33% of the samples exhibited contamination with trichothecene mycotoxins. Notably, HT-2 toxin, a type A trichothecene, was detected at a concentration of 45.44 μg/kg in one of the packaged cereals. Additionally, the highest levels of the type B trichothecene DON and its two acetylated derivatives, 3-acetyl and 15-acetyl DON, were found in packaged cereal, totaling 3199 μg/kg, which exceeded the FDA pediatric limits and was 7.7 times the TDI.

Only two packaged cereals contained either AOH or AME, with AOH at 6.0 μg/kg and AME at 3.6 μg/kg. AOH, AME, ENNs, BEA, and CPA are not currently monitored in the U.S., China, or Europe.

### 3.4. Pasta Samples (n = 13)

The pastas tested were wheat-based, with 92% of the samples containing at least one mycotoxin. In the analyzed samples, the emerging mycotoxins ENNs, BEA, and AME were the most commonly co-occurring compounds. Specifically, 72% of the samples contained at least one of the ENNs; AME was quantified in 62% of samples, and BEA was present in 38%. Additionally, HT-2 toxin and DON were identified in two separate samples, and AFB1 was found in one sample. Lastly, OTA was quantified in three samples, representing 23% of the total.

### 3.5. Raw Ingredients (n = 46)

Raw ingredients are often considered minimally processed because they typically undergo fewer food-processing steps than ultra-processed foods. A total of 46 ingredients were tested in this study. The tested ingredients included wheat flour, corn flour, almond flour, coconut flour, arrowroot, tapioca starch, and chocolate powder.

#### 3.5.1. Grain-Based Ingredients

Wheat flour (*n* = 10) was contaminated with two or more mycotoxins in 90% of the samples, with a maximum of 13 quantifiable mycotoxins in one sample. Furthermore, 30% of the samples were contaminated with one or more trichothecene mycotoxins, including DON and HT-2 toxin, which co-occurred in two of the three samples. Fifty percent of the samples contained low levels of aflatoxins, and 70% contained at least one ENN and one or more *Alternaria* species mold toxins. EnnB, EnnB1, AOH, and AME were the most frequently co-occurring compounds in these samples. BEA was detected in only one sample. Additionally, fumonisins were detected at low levels in 40% of the samples, and OTA and ZEA were detected in one sample each.

Corn flour was found to be more contaminated than wheat flour. Corn flour samples (*n* = 21) were 100% contaminated with three or more mycotoxins. In addition, 100% of the samples were contaminated with AFB1, 71% with AFB2, and 19% with AFG1 at quantifiable levels, with 43% exceeding the FDA guidance levels, raising concerns about hepatotoxicity. The highest detected concentration of combined AFB1, AFB2, and AFG1 in corn flour was 273.8 μg/kg, whereas the median concentrations among the samples were 11.9, 1.7, and 2.2 μg/kg, respectively.

The aflatoxin precursor, STC, was detected in 21 samples but was quantifiable only in one corn flour sample. CPA, a mycotoxin that frequently co-occurs with aflatoxin, was detected in 24% of the samples, with the highest concentration being 3804 μg/kg. Additionally, 57% of the samples contained at least one type A or type B trichothecene mycotoxin, or both. One sample contained the trichothecene mycotoxins DON, T-2, and HT-2 toxins (at 900, 71.2, and 49.6 μg/kg, respectively), along with aflatoxins, AME, AOH, and EnnA1. Fumonisins were quantified in 57% of the samples, EnnA1 in 71%, and AOH and AME in 19% of the samples. The most common co-occurrences were between EnnA1, AFB1, AFB2, fumonisins, and DON and its masked derivatives. All but EnnA1 have been studied for their relationship with growth stunting in children [79,80].

Corn-based flour emerged as the commodity with the most significant contamination. This study identified corn flour as containing the highest concentrations of AFB1, AFB2, T-2, HT-2, ZEA, fumonisins, CPA, DOM, and DON-3-glucoside. Notably, the AFB1 and AFB2 levels occasionally exceeded the regulatory limits set by the FDA and European authorities. Furthermore, all corn flour samples exceeded the regulatory limits established for pediatric use in China.

#### 3.5.2. Non-Grain Ingredients

All almond flour samples (*n* = 4) exhibited trace amounts of aflatoxins, which were significantly lower than the levels detected in corn flour and remained well below the FDA regulatory thresholds. One sample contained all four ENNs, BEA, and AFB1. In the analysis of coconut flour (*n* = 3), one sample was devoid of mycotoxins, while the remaining two samples exhibited contamination with all four ENNs and BEA.

Arrowroot and tapioca starch (*n* = 4) were contaminated in 50% of the samples. One tapioca sample was contaminated with all four ENNs, BEA, and AFB1, and arrowroot was contaminated with low levels of 15-ace (100 µg/kg). Two samples were found to be free of mold toxins.

Chocolate powder (*n* = 4) was also tested because of its popularity as an ingredient. Four samples were tested, and all of them (100%) contained trace amounts of various contaminants. All samples contained at least one quantifiable mycotoxin, typically trace amounts of AFB1 or AFG1, but were within FDA and European limits. Only one sample conformed to the Chinese guidance levels for AFB1 in children. Another sample contained six mycotoxins, encompassing all four ENNs, BEA, and AFB1.

### 3.6. Comparative Mycotoxin Detection Across Heterogeneous Sample Matrices

In general, ENNs were present in only 27% of the non-grain-based samples, and BEA was present in 33% of the non-grain-based samples. Trace amounts of aflatoxins were detected in 60% of non-grain ingredient samples, but all levels were well below the FDA and European safety guidance levels. However, all non-grain ingredients contaminated with AFB1 exceeded the Chinese limit of 0.5 μg/kg for foods marketed for pediatric consumption. The most common co-occurrences in non-grain-based flours were AFB1, ENNs, and BEA. However, non-grain flours and other ingredients were not significantly contaminated with trichothecene mycotoxins compared to grain-based foods. Only one sample contained 100 µg/kg of 15-ace, which is below the FDA, Chinese, and European pediatric guidance levels, and no other trichothecene mycotoxins, fumonisins, or ZEA were detected in the non-grain ingredients. Finally, *Alternaria* spp. mold toxins AOH and AME were not found in any of the non-grain ingredients. They were only detected in processed grain-based ingredients and ultra-processed foods.

In total, corn-based products had the highest contamination rates for trichothecene mycotoxins, ZEA, fumonisins, aflatoxins, and CPA. OTA was most frequently detected in wheat-based foods, with only one chocolate sample containing trace amounts. ENNs and BEA were detected across all categories but were most prevalent in processed cereals, snack foods, and apple juice. AOH and AME were most frequently found in wheat flour, pasta, and toddler foods (i.e., with prevalences of 70%, 62%, and 57%, respectively), relative to sample size, and are currently not regulated in the US, China, or Europe. Table 5 illustrates the extent to which regulated mycotoxins surpassed the European TDI limits at both the highest and median quantifiable levels in a single toddler-sized serving. Values below the LOQ were excluded from the quantitative calculations in Table 5 because their concentrations could not be measured with sufficient accuracy and precision, resulting in substantial uncertainty. Additionally, the calculated exposure estimates may underestimate potential health risks because they do not account for synergistic toxicity from co-occurring mycotoxins in the same sample, which may amplify adverse biological effects.

## 4. Discussion

Based on the findings of this survey, pediatric exposure to mycotoxins through processed foods in the U.S. appears to be common rather than incidental. Through the routine consumption of processed foods, toddlers are likely to be exposed to a broad spectrum of toxins. Notably, both regulated and emerging mycotoxins frequently co-occurred in the same food products, which raises concerns about the potential for simultaneous exposure to increase overall toxicity and health risks, particularly in young children [13,87,88]. Many mycotoxins share biological targets and mechanisms of toxicity, including mitochondrial dysfunction, inhibition of protein synthesis, oxidative stress, immune dysregulation, disruption of intestinal barrier function, and activation of apoptotic and ferroptotic pathways [36,41,89,90,91]. This raises concerns that co-exposure could lead to additive or synergistic toxic effects.

Recent surveys of cereal-based foods intended for infants and children from other countries have also reported the frequent detection of *Fusarium*-produced mycotoxins, including DON, fumonisins, ZEA, ENNs, and BEA, often occurring simultaneously in the same products [80]. In a 2025 Portuguese survey of 148 cereal-based foods for infants and children, 43% of samples contained at least one mycotoxin, with co-occurrence most commonly involving fumonisins, BEA, and ENNs. Hazard index calculations suggested that combined exposures may pose a greater concern than individual toxins considered separately [80]. Similarly, biomonitoring studies in UK children have demonstrated frequent dietary exposure to multiple mycotoxins (e.g., DON, NIV, T-2/HT-2 toxins, ZEA, OTA, and aflatoxins), with co-exposure to three or more mycotoxins observed in 66% of urine samples and frequent exceedances of health-based TDI guidance values for several *Fusarium* toxins [92].

Surveys of naturally contaminated foods worldwide further indicate that co-occurrence is the rule rather than the exception in cereals and cereal-derived products [93]. Recent cohort studies have documented widespread simultaneous exposure to aflatoxins, fumonisins, DON, and OTA in young children and have suggested that these contaminants may independently contribute to growth impairment and environmental enteric dysfunction [47,79,94]. Collectively, these studies support the present findings and suggest that dietary exposure assessments focusing on individual mycotoxins may underestimate the complexity of real-world exposure among young children.

While major mycotoxins are routinely monitored in the U.S, China, and Europe, many regulatory limits and health-based guidance values are generally derived from studies evaluating individual compounds and do not routinely account for multi-mycotoxin exposure. Furthermore, the unique vulnerabilities of toddlers, whose dietary intake relative to body weight is substantially greater than that of older children and adults, are not always considered within regulatory guidelines (see Appendix B). Thus, while few data are available on the combined effects of co-occurring mycotoxins, multi-mycotoxin effects may be of paramount importance to child health. Considering this, while the major mycotoxins are discussed here within the context of TDI limits, the overlapping effects of multiple mycotoxins will be addressed where possible.

### 4.1. Aflatoxins and Sterigmatocystin (STC)

Aflatoxins and STC are structurally related difuranocoumarin mycotoxins produced by *Aspergillus* species (spp.), with STC being aflatoxin’s xanthone precursor. Aflatoxins can contaminate most major crop plants and are well-established group 1 human hepatocellular carcinogens [7,31,95]. However, interindividual susceptibility to aflatoxin-induced carcinogenesis is believed to be influenced by host factors, including genetic polymorphisms in DNA repair pathways and co-existing hepatitis infections, which are difficult to ascertain in risk assessments [32,33,81]. Aflatoxins may also contribute to growth stunting in children by inhibiting protein synthesis, among other mechanisms [48].

Increasing evidence also indicates that aflatoxins, particularly AFB_1_ and aflatoxin M_1_ (AFM_1_), adversely affect intestinal and immune health. Animal and human cell line studies have demonstrated that aflatoxins disrupt intestinal epithelial integrity by promoting inflammation, compromising tight junctions, and increasing intestinal permeability [96,97,98,99]. When aflatoxins co-occur with other mycotoxins, such as trichothecenes, their deleterious effects on gut barrier function may be amplified, potentially contributing to immune suppression, abdominal pain, vomiting, and GI distress [88,98,100].

Mechanistically, aflatoxins have also been shown to induce ferroptosis, a regulated form of cell death distinct from apoptosis, necrosis, and autophagy [89]. Ferroptosis is driven by iron-dependent lipid peroxidation and requires intracellular ferrous iron (Fe^2+^), which catalyzes the formation of ROS via Fenton chemistry [89]. This process leads to the peroxidation of polyunsaturated fatty acids in cell membranes, cellular and mitochondrial injury, and depletion of intracellular glutathione reserves [89]. Dietary exposure to AFB_1_ may therefore promote early cellular death, hepatic injury, and disruption of iron homeostasis at sufficiently high exposure levels. Notably, DON, ENNs, and OTA, among other mycotoxins, have been shown to induce ferroptosis in mammalian cells, suggesting a shared mechanism of toxicity that potentially affects iron homeostasis among these co-occurring contaminants [89,101]. All were well represented in these samples.

Regulatory limits for aflatoxins have been established in the U.S., China, and Europe. In the U.S., the FDA permits up to 20 μg/kg total aflatoxins in foods, while Europe permits 2–12 μg/kg AFB1 and 4–15 μg/kg total [102,103]. China monitors foods for AFB1 only and has stringent guidance of 0.5 μg/kg in foods for infants and children [104]. The Joint FAO/WHO Expert Committee on Food Additives (JECFA) has evaluated aflatoxins on multiple occasions (e.g., 1997, 2001, 2017) and concluded that a TDI or provisional maximum tolerable daily intake (PMTDI) cannot be established for aflatoxins due to their genotoxic and carcinogenic properties [81]. Nonetheless, a proposed intake range of 0.017–0.082 μg/kg bw/day for total aflatoxins has been suggested based on two earlier animal studies in 1987 and 1989 that evaluated immune system effects in mice and observed significant reductions in white blood cell counts at these levels [32,81]. For risk characterization, the lower bound of 0.017 μg/kg bw/day was considered a more conservative and protective estimate for young children and was applied in the present TDI analysis.

Using the maximum observed contamination level of 273.8 μg/kg combined aflatoxins, a toddler consuming just a 1-ounce (30 g) sample of the affected product could ingest 0.66 μg/kg bw/day—nearly 38.7-fold higher than the proposed limit value of 0.017 μg/kg bw/day. At median combined contamination levels of AFB1, AFB2, and AFG1 (e.g., 5.1 μg/kg), which are significantly below the FDA regulatory thresholds, exposure constituted approximately 72% of the proposed limit for a small child. These exposures may accumulate over the course of a day. Notably, the median levels exceeded the Chinese guidance levels for infants and small children. These findings underscore the need for improved surveillance, more robust exposure data, and stronger regulatory oversight to protect young children from potentially harmful aflatoxin exposure.

STC and aflatoxins share a common xanthone–difuran core structure. STC is a biosynthetic precursor of aflatoxins and often coexists with them in samples, potentially increasing the toxicity of both. The International Agency for Research on Cancer has classified STC as a possible human carcinogen (Group 2B) and has recognized its immunotoxic properties [105]. Experimental studies have shown that STC induces hepatic and renal toxicity and causes chromosomal damage in both in vitro and in vivo models [86,105]. However, due to limited occurrence and exposure data in humans and animals, the EFSA CONTAM Panel has been unable to fully characterize the associated human health risks [86]. In this survey, STC was detected in 21 samples but was quantifiable in only 1 corn flour sample at 5.7 μg/kg (LOQ 0.5 μg/kg). There is a need for larger and more representative datasets to support meaningful exposure assessments and risk characterization.

### 4.2. Trichothecene Mycotoxins

Foodborne trichothecenes are primarily produced by *Fusarium* spp. fungi. Trichothecene mycotoxins possess a tricyclic 12,13-epoxytrichothec-9-ene (EPT) nucleus, which is the principal determinant of toxicity [31]. Multiple trichothecene mycotoxins were detected in the samples analyzed. Globally, trichothecenes most commonly infect wheat, corn, barley, rye, oats, millet, triticale, rice, sorghum, grain-derived alcoholic beverages, soy, tea, and dried spices [106,107].

Trichothecene mycotoxins are well known for their adverse effects in animals and humans, including gastroenteritis, nausea, vomiting, anorexia, impaired immune function, fever, headache, and growth retardation [9,11,12,13,14,16,19,20,21,49,52,53]. Chronic exposure to DON, a highly prevalent mycotoxin in food, has been demonstrated to compromise gut barrier integrity, disrupt intestinal mucosal immunity, alter gut microbiota homeostasis, impair mitochondrial function, and promote inflammation through an increase in reactive oxygen species (ROS) and oxidative stress [6,89,108]. Owing to their harmful effects on intestinal structures and immune regulation, trichothecenes are believed to play a role in IBD and related GI disorders [6,16,31,88,103,109,110].

Although trichothecenes, such as DON and its acetylated derivatives, were detected less frequently in this study than in previous surveys, their presence was notable and often occurred alongside other mycotoxins [6]. The highest concentration of DON, a type B trichothecene, was 2499 μg/kg in a processed cereal. This is five times the FDA limit, approximately 12 times the Chinese limit, and 12 times the European limit for infants and children. Several DON-related metabolites and modified forms, including 15-Ace, 3-Ace, DON-3-Glu, and DOM, were detected in varying amounts. Most of these have effects similar to those of the parent compound DON, except for DOM, which may be less toxic [111,112].

To better conceptualize the risk of toxicity, a toddler consuming a 1-ounce (30 g) serving of a cereal product contaminated with 2499 μg/kg DON would exceed the TDI by 6-fold [83,113]. At a median DON level of 300 μg/kg in a single 1-ounce serving, the intake would be approximately 72% of the TDI, with exposure potentially increasing throughout the day. The occurrence of 3- and 15-acetylated and 3-glucoside derivatives of DON in the same packaged cereal may intensify the situation. These derivatives are expected to produce inflammatory effects on gastrointestinal and immune system tissues, similar to those of DON [17,114,115,116,117,118].

Potentially exacerbating this, the type B trichothecene NIV was detected in four samples, none of which reached the LOQ. NIV’s mode of action is similar to that of DON’s; however, the FDA has not set limits for this toxin. While NIV is not routinely regulated in the U.S. or China, the European Union has established a TDI of 1.2 μg/kg bw/day [119]. In a recent study on multi-mycotoxin exposure in UK children, although NIV exposure from food was below the LOQ, 4.39% of urine samples exceeded the TDI [92]. The occurrence of NIV alongside other group B and A trichothecenes raises concerns due to their potential additive or synergistic effects on the human gastrointestinal tract, as well as the immune and nervous systems. Although exposure studies have confirmed the presence of multiple trichothecenes in children, there is a paucity of research examining their additive, synergistic, or antagonistic interactions with multiple mycotoxins in children, beyond the context of growth stunting [47,79,92].

DON and other trichothecene mycotoxins, including T-2 and HT-2 toxins, have also been observed to induce ferroptosis in both intestinal and other organ tissues in animals and in vitro. This process potentially disrupts redox reactions and iron metabolism [89,120]. Such disruptions could be particularly harmful to developing children when these mycotoxins occur together as a “mycotoxin cocktail”.

Furthermore, type A trichothecenes, T-2 and HT-2 toxins, are considered more potent than many type B trichothecenes and were well represented in samples. Both have been found to target the GI, epidermal, immune, and hematopoietic systems [84]. Neosolaniol (NEO), another type A trichothecene, was also detected in 13 samples, although the concentrations were below the LOQ, further indicating the presence of multiple type A trichothecenes in toddler foods and ingredients. These toxins are readily absorbed by the GI tract, skin, and lungs [56]. At high exposure levels, T-2 and HT-2 toxins have been shown to be lethal. This was evidenced during outbreaks of alimentary toxic aleukia (ATA) in the Orenburg region of the former Soviet Union during World War II, where the mortality rates among children under 10 years of age consuming contaminated grains approached 50% [5].

T-2 toxin is rapidly metabolized to HT-2 toxin following ingestion; therefore, these two compounds are considered together in risk assessments [84]. Considering the toxicity of these compounds, the European Food Safety Authority (EFSA) has established a TDI of 0.02 μg/kg bw/day for the combined intake of T-2 and HT-2 toxins [84]. HT-2 toxin was detected in 13 samples, while T-2 toxin was present in 9 samples. The maximum concentration of T-2 toxin, measured at 49.6 μg/kg, was observed in corn flour. Similarly, the highest HT-2 toxin level, 71.2 μg/kg, was detected in corn flour, which contained at least three mycotoxins per sample. The median concentrations of T-2 and HT-2 toxins were 34.7 μg/kg and 9.5 μg/kg, respectively, and these were identified in various processed foods.

Based on a 1-ounce (30 g) serving size, the estimated exposure for a toddler would exceed the TDI by approximately 6-fold and 8.5-fold at the highest detection levels for T-2 and HT-2 toxins, respectively, and by 4-fold and 1.1-fold at the median detection levels. T-2 and HT-2 toxins co-occurred in a single corn flour sample, exceeding the TDI by 14.5-fold. They were detected alongside other mycotoxins in wheat flour, snack, and cereal samples. Kimanya et al. [94] also reported that children in Tanzania were exposed to multiple mycotoxins through the consumption of corn.

Despite their documented toxicity, NIV, T-2, and HT-2 toxins, as well as acetylated derivatives and masked forms of DON, are not currently regulated or routinely monitored in foods in the U.S. or China, in contrast to European regulatory frameworks, raising serious concerns regarding child safety [83,104,113].

### 4.3. Fumonisins (FB1, FB2, and FB3)

Fumonisins are a family of water-soluble mycotoxins primarily produced by *Fusarium* fungi and are characterized by a long-chain amino polyol structure (i.e., polyketide). Fumonisins are typically detected alongside other *Fusarium*-produced mycotoxins in cereal grains, as observed in this study [45].

The International Agency for Research on Cancer has identified FB1 as a potential carcinogen [9]. Studies have shown that FB1, one of the three fumonisins believed to affect human health, significantly increases the risk of esophageal cancer in humans [45]. It has also been found to interfere with folic acid and sphingolipid metabolism, potentially increasing the risk of neural tube defects in infants [30].

Eating foods with high concentrations has also been demonstrated to affect intestinal health, leading to abdominal pain, borborygmi, and diarrhea in humans, and to increase the number of lymphocytes and monocytes in the ileum and cecum of the intestines [45]. Fumonisins have also been found to increase intestinal permeability (i.e., leaky gut) and the growth of harmful bacteria, such as pathogenic *E. coli*, in various parts of the intestines (e.g., ileum, cecum, and colon) [46]. Furthermore, Ayeni et al. [121] found that fumonisins, along with aflatoxins, AME, and CIT, have been implicated in disrupting the infant microbiome, potentially increasing the abundance of *Klebsiella* and *Clostridium difficile* taxa.

Twenty-one samples contained quantifiable levels of FB1, with the highest at 2000 μg/kg and a median of 500 μg/kg in corn flour for FB1, FB2, and FB3 combined. FB1 was the most prevalent of the three. In 2018, the EFSA established a tolerable TDI for fumonisins of 1.0 µg/kg bw/day [82]. Using a 30 g serving to calculate risk showed that a toddler would consume approximately 4.7 times the TDI at the maximum and 1.2 times the median amount, demonstrating that exposure can add up quickly in a small child, even when levels are within federal regulatory limits.

Fumonisins are regulated in the U.S., China, and Europe, but were frequently detected alongside other mycotoxins produced by *Fusarium* spp., such as DON, T-2, and HT-2 toxins, as well as toxins from *Aspergillus* molds, including aflatoxins and CPA, in this study. This co-occurrence raises concerns about the combined and additive effects of these toxins on various organ systems, including the intestinal, immune, and nervous systems, as well as the microbiota [45,46,121].

To date, most research has concentrated on pediatric growth stunting and enteric function within this context. For instance, the Sanitation Hygiene Infant Nutrition Efficacy (SHINE) trial is examining the effects of fumonisins, DON, and aflatoxins on enteric dysfunction and growth stunting in infants and children [47]. Similarly, in the AflaCohort study in Nepal, Andrews-Trevino et al. [79] observed that aflatoxin exposure negatively impacted linear growth, with serum aflatoxin and fumonisin levels correlating with underweight, and urinary DON levels associating with reduced head circumference in toddlers, independent of enteric dysfunction. Given these findings, examining the combined impact of these and other mycotoxins on child health is crucial.

### 4.4. Ochratoxin A (OTA)

OTA is a chlorinated, low-molecular-weight mycotoxin with a distinctive isocoumarin–amino acid hybrid structure produced by *Aspergillus* and *Penicillium* fungal species [30]. Chronic exposure to OTA has been shown to damage renal tubular cells and impair kidney function, and has been historically implicated in Balkan endemic nephropathy, a fatal chronic kidney disease affecting populations in Bulgaria and surrounding regions [9,30]. Subsequent studies have linked OTA exposure to testicular cancer, urinary tract tumors, and potential neurotoxicity, including a proposed contributory role in ASD [8,9,23,24,25,122]. De Santis et al. [23] studied differences in the metabolism of DON, aflatoxin M1, FB1, and OTA between children with ASD and non-ASD controls; they found differences in the detoxification and metabolism of these mycotoxins [23]. More research on the influence of combined mycotoxin exposure on neurodegenerative diseases is needed, as such co-exposure may be common.

OTA was detected in six samples, with a maximum concentration of 2.1 μg/kg in wheat flour and a median concentration of 1.2 μg/kg in wheat-based pasta. The EFSA has established a tolerable weekly intake (TWI) of 0.120 μg/kg bw/week, corresponding to an average daily intake of approximately 0.017 μg/kg bw/day when averaged over a week. Based on this benchmark, the estimated exposure derived from the maximum quantifiable OTA sample (wheat pasta), a single 70 g serving, was approximately 0.0118 μg/kg bw/day, or 69% of the daily-equivalent limit, and 39% of the median level. This suggests that intake from individual food items may fall below current safety thresholds [85].

However, these risk estimates do not account for the cumulative exposure from multiple foods consumed over the course of a day or week. Nor do they consider OTAs’ limited metabolic clearance in humans. OTA is frequently detected in human biomonitoring studies, a finding likely attributable to its prolonged biological half-life and resistance to enzymatic degradation [123]. Compared with the structurally related ochratoxin B, OTA persists longer in the body, in part due to the presence of a chlorine atom at the 5-position of the isocoumarin ring, which increases its chemical stability and reduces its metabolic breakdown, possibly increasing the risk to child health [123].

Despite its persistence, nephrotoxicity, carcinogenicity, and possible neurotoxicity, OTA is not regulated in U.S. foods, highlighting a critical gap in food safety oversight for this widely encountered mycotoxin. OTA is regulated in China and the European Union.

### 4.5. Zearalenone (ZEA)

ZEA is a fungal resorcyclic acid lactone produced by *Fusarium* fungi whose chemical structure closely mimics endogenous estrogens. ZEA is a potent mycoestrogen with greater estrogenic activity than many other foodborne endocrine disruptors, including genistein, bisphenol A, and phthalates [44]. ZEA has been extensively studied for its adverse reproductive effects in humans and wildlife and has historically been used in pharmaceutical applications due to its estrogenic properties [2]. Kincade et al. [44] conducted a U.S. pregnancy cohort study pertaining to socioeconomic and dietary predictors and found that women who ate more processed diets had higher placental levels of hormone-disrupting ZEA.

In addition to its endocrine-disrupting effects, ZEA has been shown to affect GI health. At low doses, it increases the intestinal expression of inflammatory cytokines and may suppress tumor suppressor gene activity [42]. In line with this, Abassi et al. [124] found that ZEA promoted the growth of human colon cancer cells in vitro. Furthermore, its metabolites, α- and β-zearalenol, have been reported to increase intestinal permeability, potentially enhancing susceptibility to infection [43]. Both α- and β-zearalenol were detected in 4 and 1 samples, respectively, but at such low levels that they could not be quantified in this study. While such low exposures may reduce their potential to cause adverse effects in humans, this has not been definitively proven, particularly when mixed with other mycotoxins. ZEA was also detected alongside other *Fusarium* mycotoxins (e.g., DON) known for their adverse effects on the GI tract, as well as aflatoxins and CPA.

Although ZEA has potential adverse effects on humans, it is not routinely regulated in U.S. foods. This omission constitutes a considerable oversight regarding this biologically active mycotoxin, which may have implications for children’s health. However, ZEA is regulated in Europe and China, and a European TDI of 0.25 μg/kg bw/day has been established [44].

ZEA was quantified in 9 samples, with the highest concentration observed in corn flour at 126.6 μg/kg. It was also detected in processed cereal products and wheat flour, with a median level of 32.2 µg/kg. Using the tolerable daily intake (TDI) as a reference, a toddler ingesting a 30 g portion of corn flour contaminated at the highest recorded level would surpass the TDI by approximately 1.2 times. The same portion at the median contamination level in processed cereal would constitute about 31% of the TDI in a single serving. This exposure can add up rapidly throughout the day, highlighting a substantial oversight gap.

### 4.6. Emerging Mycotoxins

Emerging mycotoxins were among the most frequently detected toxins in this study. These fungal secondary metabolites are increasingly being detected in food and feed but are not yet routinely regulated or comprehensively risk-assessed, despite evidence of biological activity and potential health concerns. Consequently, they lack established maximum limits or guidance values in most countries. Furthermore, data on chronic toxicity, particularly in vulnerable populations such as children, remain sparse. TDIs for AOH, AME, BEA, the ENNs, and CPA mycotoxins detected here have not been established.

In the absence of compound-specific TDIs, the Threshold of Toxicological Concern (TTC) approach was employed here as a screening tool. Nevertheless, the uncertainty associated with these estimates can be substantial. TTC estimates are based on generalized chemical structure classes rather than specific toxicity data for individual compounds, which may not adequately consider developmental susceptibility or the combined effects of co-exposure to multiple mycotoxins. Consequently, they may either underestimate or overestimate a compound’s toxicity and should be interpreted with this consideration in mind [125,126].

#### 4.6.1. Alternariol (AOH) and Alternariol Mono-Ethyl Ether (AME)

AOH and AME are structurally related dibenzopyranone mycotoxins—AME being the O-methylated, more lipophilic form of AOH—produced mainly by *Alternaria* spp. [127]. Among *Alternaria* spp. mycotoxins, AOH, AME, and tenuazonic acid are the most extensively studied due to their cytotoxic and genotoxic properties, with AOH and AME considered among the most toxic [41,127].

Mechanistic studies have shown that both toxins exhibit endocrine-disrupting effects; are genotoxic and cytotoxic in vitro; and can induce liver, kidney, intestinal, and splenic toxicity, as well as immune dysfunction at repeated low µg/kg bw/day exposures [37,40,41]. These effects are partly initiated by DNA strand breaks, oxidative stress from increased ROS generation, and the inhibition of topoisomerases I and II, enzymes critical for DNA replication and repair [41].

In addition, co-exposure to AOH and AME, frequently observed in this study, has been shown to potentiate cytotoxic effects in human intestinal and hepatic cell lines compared to exposure to either compound alone [37]. Both have also been found to increase pro-inflammatory signaling by influencing the NF-κB signaling pathway and to accumulate in the gut, possibly disrupting the gut pH balance and microbiota diversity [41]. Evidence also suggests that these toxins exhibit greater cytotoxic, genotoxic, and estrogenic effects when they occur together or with other mycotoxins, such as DON and ZEA [41].

Occurrence data for AOH and AME in the U.S. are limited. Most analytical surveys have been conducted in Europe, Asia, and Africa, but only limited occurrence data are available from North America, primarily Canada [41,127]. However, based on the findings of this survey, chronic low-level dietary exposure to AOH and AME may be a concern in the U.S., given their potential toxic effects. Worldwide, AOH and AME have been detected in fruits, vegetables, cereals, oilseeds, and processed products such as juices and tomato-based foods, with the highest prevalence reported in cereal grains and tomato products [41]. In this study, AOH and AME were among the most prevalent contaminants detected in grain-based snacks, pasta, and processed wheat flour, but were not detected in non-grain flours or chocolate powder.

The EFSA has identified significant data gaps regarding the long-term health effects of AOH and AME and noted that, despite their frequent co-occurrence with other mycotoxins, these compounds remain unregulated worldwide [127]. Of particular concern is the potential exposure among infants and young children, for whom the EFSA has proposed a conservative TTC of 0.0025 μg/kg bw/day [127]. Given a 30 g serving of a wheat-based snack food at the maximum and median AOH concentrations of 28.4 μg/kg and 6.4 μg/kg, respectively, a toddler would exceed the TTC by 27.3- and 6.1-fold, respectively. Similarly, given the maximum and median AME concentrations of 36.4 μg/kg and 3.8 μg/kg, a toddler would exceed the proposed TTC by factors of 34.9 and 3.6, respectively. Collectively, these findings underscore the need for expanded toxicological research, improved exposure surveillance, and enhanced regulatory oversight by U.S. food safety authorities to better characterize risk and protect vulnerable pediatric populations. Although no legally binding maximum levels have been established for *Alternaria* toxins in the European Union, Commission Recommendation (EU) 2022/553 recommends monitoring AOH and AME and allowing no more than 2 μg/kg in cereal-based foods intended for infants and young children [128].

#### 4.6.2. Cyclopiazonic Acid (CPA)

CPA is an indole-tetramic acid mycotoxin produced by several *Aspergillus* and *Penicillium* species, including *Aspergillus* flavus, a common foodborne fungus capable of simultaneously producing aflatoxins and CPA [26,27]. Cereal grains, nuts, oil seeds, dried figs, milk, cheese, and processed meats are the foods found to be most contaminated with CPA [26].

Although CPA has been extensively studied in animal models—including rats, pigs, guinea pigs, poultry, and dogs—human toxicological data remain limited. Experimental animal studies have indicated that even low-dose exposure can induce inflammation of the GI tract, liver, and kidneys, as well as neurological disturbances [27,28]. Reported toxic effects include severe GI necrosis similar to necrotizing enterocolitis, tremors, and other neurological manifestations [27]. In addition, CPA has been shown to impair immune function in human cell lines and has been implicated as a possible causative agent in Kodo (Kodua) poisoning outbreaks associated with mold-contaminated millet in northern India [26].

Mechanistically, CPA is of particular concern because it acts as a potent and specific inhibitor of endoplasmic reticulum Ca^2+^-ATPase and exhibits strong metal-chelating activity, thereby disrupting calcium homeostasis and potentially impairing mineral absorption and sufficiency in children [27].

Burdock and Flamm [29] and DeWaal [28] discussed establishing a safe CPA TDI limit for humans at the turn of this century. DeWaal proposed that a safe TDI limit would be 0.1 μg/kg bw/day [27,28,29]. However, due to the scarcity of in vitro and animal data on chronic toxicity, a TDI has never been established. Regardless, using the proposed number may provide general information about the level of toxicity that a toddler may experience.

In the present survey, CPA was detected in contaminated corn flour and corn-based products at high and median concentrations of 3804 µg/kg and 134.8 µg/kg, respectively. Using DeWaal’s proposed conservative intake level, a toddler consuming a 30 g serving would exceed the suggested safety threshold by 91-fold at high levels and 3.2-fold at the median level. Given the documented toxic effects of CPA in animal models, including GI injury and, in some cases, necrotizing enterocolitis-like pathology, this level of exposure is concerning [27,28,29].

In addition, other mycotoxins, including fumonisins, ENNs, BEA, AFB1, DON, and ZEA, were detected in corn flour, packaged cereals, and snack foods. The toxicological implications of these combined exposures are yet to be determined. A study conducted in Portugal, which assessed 20 mycotoxins in 148 cereal-based foods for infants and children, also identified frequent co-occurrence of these toxins, albeit at a lower frequency than observed in the present study [80]. These findings highlight the pressing need for further research to more accurately characterize the human health risks associated with CPA and the physiological effects of multi-mycotoxin exposure, particularly in infants and young children. Such research is essential to inform evidence-based worldwide regulatory guidance.

#### 4.6.3. Enniatins A, A1, B, and B1 (ENNs) and Beauvercin (BEA)

ENNs and BEA are emerging cyclic hexadepsipeptide mycotoxins primarily produced by *Fusarium* spp. and *Beauveria bassiana* [129]. These toxins are more commonly synthesized in milder climates by species such as *Fusarium avenaceum* and can proliferate during post-harvest transport and under suboptimal storage conditions [130]. ENNs and BEA are lipophilic ionophores that readily integrate into cellular membranes and form cation-selective pores, thereby disrupting ionic homeostasis [34,36,129,131]. By facilitating the transmembrane movement of mono- and divalent cations, these toxins induce mitochondrial membrane depolarization and dysfunction—effects demonstrated in vitro in human cell lines and in vivo in animal models [34,36,132].

Toxicological studies have indicated that ENNs and BEA exhibit a broad range of adverse biological effects, including cytotoxicity, mitochondrial damage, antimicrobial activity, intestinal toxicity, and hepatotoxicity [34,35,63,90,101,108,129,133]. Proteomic analyses of acute EnnB and BEA exposure in the rat liver have demonstrated hepatotoxic potential, with altered expression of proteins involved in metabolic regulation, oxidative stress reactions, mitochondrial function, and acetylation pathways, leading to metabolic disturbances [34]. Complementing these findings, Wang et al. [131] reported that exposure to ENNs and BEA in 3D HepaRG human hepatic cell models disrupted glycerophospholipid and sphingolipid metabolism, promoting hepatic lipid accumulation, oxidative stress, and chronic inflammatory signaling. Similar metabolic derangements have been described in the development of liver diseases, such as non-alcoholic fatty liver disease (NAFLD), steatohepatitis, and potentially hepatocellular carcinoma [131,134].

Experimental evidence further suggests that ENNs and BEA may induce ferroptosis as a mechanism of liver injury [36,89,131]. Söderdam et al. [101] demonstrated that EnnB and BEA were cytotoxic to salmon hepatocytes at low concentrations and disrupted glutathione metabolism while enriching ferroptosis-associated pathways. Although human exposure data remain limited, these findings warrant further investigation into the potential links between ENNs and BEA exposure, iron dysregulation, liver disease, and anemia, particularly in pediatric populations. Furthermore, both toxin groups have been linked to disruptions in the microbiome and immunomodulatory effects. Given the frequent co-occurrence of ENNs and BEA, their combined exposure may result in synergistic toxic effects, potentially increasing health risks in young children [55,108,129,133].

In this study, ENNs and BEA were frequently detected and quantified, occurring in 42% and 44% of samples, respectively. Worldwide, ENNs have been reported in grain surveys, with occurrences ranging from 12% to 100% [129]. BEA has been similarly reported in cereal grains, including wheat, rye, oats, barley, and rice, at prevalence rates ranging from 40% to 90% globally [55,129,132].

At present, insufficient toxicological data are available to establish substance-specific TDIs for the ENNs or BEA. Consequently, the EFSA has applied a TTC of 1.5 μg/kg bw/day for ENNs, while cautioning that tolerable exposure levels for BEA may be substantially lower due to its genotoxic potential, and has suggested a TTC of 0.0025 μg/kg bw/day [135]. Notably, the highest levels of ENNs and BEA in this survey were 308.1 μg/kg of combined ENNs in baby apple juice and 190.3 μg/kg of BEA in processed cereals. A 70 g serving of juice at this level of ENNs would correspond to approximately a 1.2-fold exceedance of the TTC, and a 1-ounce (30 g) serving of cereal would correspond to a 182.7-fold exceedance for BEA. The median concentrations across all samples were 41.6 μg/kg for ENNs and 8.3 μg/kg for BEA, corresponding to approximately 16% of the TTC for ENNs and an 8-fold exceedance for BEA. ENNs and BEA often occurred alongside DON, fumonisins, ZEA, AFB, and CPA in this study, creating a mycotoxin mix in foods with uncertain effects. Overall, the concentrations and detection frequencies observed here underscore the need for expanded surveillance, refined exposure assessments, and targeted toxicological research in the U.S., particularly to evaluate the risks to infants and toddlers [136].

### 4.7. Public Health and Policy Implications

Mycotoxins have contaminated human food supplies for millennia, and mycotoxin-associated illnesses have been documented across diverse geographic regions [5,9,30,56,57]. In modern globalized food systems characterized by large-scale sourcing, prolonged storage, extensive processing, and long-distance transport, this longstanding problem is increasingly transboundary—particularly in settings with inadequate Hazard Analysis and Critical Control Point (HACCP) controls [41]. Climate change further amplifies these risks, as temperature shifts, drought, and excessive rainfall create conditions that favor fungal infection and mycotoxin production in crops, which may then be distributed across regional and international markets [137,138,139]. Even crops that appear unaffected at harvest may become contaminated during transport, storage, or processing if hygiene and climate controls are insufficient, increasing the likelihood of mycotoxin entry into the food supply [137,140,141].

Many foodborne contaminants—including several emerging mycotoxins such as AOH, AME, CPA, ENNs, and BEA—are not routinely monitored or regulated in many regions (including the U.S.), largely due to limited human toxicological and epidemiological data. Consequently, these compounds remain understudied, despite growing evidence of their widespread occurrence in commonly consumed foods and their frequent detection in this study.

Furthermore, contamination may arise from multiple ingredients in a single product, including primary components such as cereal flours and minor additives such as spices and flavorings. This complexity allows multiple fungal species to contaminate the same food matrix, resulting in co-occurring mycotoxins with potentially additive, synergistic, or even antagonistic effects with unknown consequences. The growing recognition of these cumulative exposures and the need for more research have prompted the Food and Agriculture Organization of the United Nations and the World Health Organization to re-examine global agricultural and food-processing practices related to mycotoxin contamination [11].

Several interrelated agricultural factors are believed to contribute to increasing fungal infections in crops, including climate change, suboptimal crop rotation practices, the heavy use of agrochemicals that disrupt soil ecosystems, and insect damage, among other agronomic variables [110,140,142,143,144,145,146,147,148]. Although good agricultural practices remain crucial for prevention, evidence suggests that they may become increasingly inadequate as climatic conditions evolve. In a 15-year longitudinal study in Croatia and Serbia, Kos et al. [139] demonstrated that shifts in temperature, precipitation, and humidity were associated with increased DON contamination across multiple cereal commodities.

Amidst climate-driven and agronomic pressures that promote fungal growth and mycotoxin formation, chemical control methods have shown limited success. Pesticides and fungicides have not reliably suppressed mycotoxin production and may contribute to human toxicity, promote azole-resistant fungal species, and increase CO_2_ emissions by disrupting soil ecosystems, thereby exacerbating climate-related pressures on fungal growth [110,149,150,151,152,153]. Moreover, metal-chelating herbicides, such as glyphosate, may paradoxically promote the growth of fungal pathogens (e.g., *Fusarium* spp.) in human food crops by killing off competing microbes and weakening plant immune defenses by making various chelated metals, such as manganese, unavailable for mounting an immune response [144,154,155]. Kremer and Means [146] demonstrated that the rates of infection by three mycotoxin-producing *Fusarium* spp. were increased by two- to five-fold in glyphosate-treated soybeans at recommended application rates. Potentially exacerbating this problem, certain fungicides may paradoxically enhance mycotoxin biosynthesis in pathogenic fungi. For instance, strobilurin fungicides have been shown to increase DON concentrations in harvested grains by approximately 6–18% following application [148,156,157]. In contrast, biological approaches, including mycotoxin-degrading microorganisms and the restoration of soil microbial ecosystems, represent promising alternatives for long-term mitigation [100,158,159,160,161,162]. Adsorbent materials, such as clay-based products, activated charcoal, and seaweed-derived biopolymers, have also shown efficacy in protecting animals fed with contaminated feed [163].

In addition to crop management, post-harvest handling further influences the risk of mycotoxin contamination. Global trade facilitates the transboundary movement of fungal spores, with the country of origin strongly influencing both fungal species distribution and contamination levels [150,151]. Fungal spores can also persist on agricultural and food-processing equipment and within storage facilities despite routine cleaning, and their germination is strongly influenced by temperature and humidity [110,141]. In large-scale storage systems with limited climate control, maintaining conditions unfavorable to mold growth is often impractical, particularly for fungi that require humidity levels below 20% for suppression [164]. Extended storage periods prior to processing, driven by food and food processing needs across regions, further increase the likelihood of contaminated commodities entering the global food supply [110].

Once present in food, many mycotoxins are difficult, if not impossible, to eliminate due to their heat stability and resistance to pH extremes, making prevention the most effective mitigation strategy [106,165,166]. Furthermore, the industrial use of preservatives to prevent or mitigate toxigenic fungi and mycotoxins may be less effective than previously thought. Although many fungi can be killed by chemical preservatives, their use may induce stress responses that trigger mycotoxin release. Zhelifonova et al. [167] found that a 0.015% sodium nitrate solution did not impair CPA production. They also found that a 0.015% sodium benzoate and 0.02% potassium sorbate solution caused a 1.5-fold increase in CIT, CPA, and mycophenolic acids, and a 1.7-fold and 2.6-fold increase in CPA and mycophenolic acids, respectively [167]. Lee et al. [168] found that the use of preservatives as food additives may pose additional health risks, as they may disrupt the human microbiome and have been linked to the development of IBD in pediatric populations. Accordingly, robust agricultural practices must be complemented by effective post-harvest controls, including storage and transport management and HACCP-based food safety systems that protect the public [41].

While some argue that completely preventing mycotoxin exposure from processed foods may be impossible, especially for vulnerable groups such as children, significant reductions in exposure can still be achieved [110,164]. Indeed, promoting increased consumption of fresh, whole, locally harvested, organically farmed foods rather than processed foods may reduce the likelihood that children will eat foods contaminated during transit, production, and storage. These practices could help keep such infections contained within a region [8].

Protecting vulnerable pediatric populations will likely require more protective regulatory thresholds, the inclusion of emerging mycotoxins in monitoring frameworks, and strengthened oversight of food manufacturing and processing practices. Enhanced agricultural surveillance, including oversight of crop rotation, pest management strategies, harvest timing, and on-farm storage, may enable earlier detection and prevent highly contaminated commodities from entering food-processing streams.

Finally, research on foodborne mycotoxins has historically remained largely siloed away from clinical and public health practice, limiting the translation of scientific findings into prevention and risk-reduction strategies. Consequently, healthcare professionals often receive little formal education on dietary mycotoxin exposure or its potential relevance to pediatric health issues. This gap is increasingly concerning in light of the rising rates of chronic pediatric conditions—including IBD, IBS, ASD, and certain liver diseases, among others—whose etiologies remain incompletely understood and may overlap with the biological effects observed in chronic mycotoxin exposure. To address these issues, it is necessary to establish coordinated cross-sector collaborations among healthcare providers, researchers, agricultural scientists, food producers, and policymakers. This collaboration should focus on developing and implementing comprehensive, long-term strategies to reduce exposure and safeguard child health.

### 4.8. Limitations and Future Research Implications

This study identified multiple regulated, unregulated, and emerging mycotoxins in foods commonly consumed by U.S. children, suggesting the potential for chronic, low- to high-dose dietary exposure during a sensitive developmental period. The frequent co-occurrence of mycotoxins suggests that toddlers may experience daily combined exposures, with cumulative intake increasing throughout the day. However, the interpretation of these findings must account for several limitations within the established EFSA risk assessment frameworks [135,169].

First, this cross-sectional survey of commercially available processed and ultra-processed foods was not representative of the broader U.S. food supply. Regional, seasonal, and supply chain variability, recognized by the EFSA as critical determinants of exposure, were not captured, underscoring the need for larger, randomized, and nationally representative studies to improve the generalizability and accuracy of risk assessments [169]. This initial screening assessment indicates the need for a significantly larger study.

Second, although 34 mycotoxins, including masked or modified forms, were quantified using LC-MS/MS, the analytical panel did not cover the full range of fungal secondary metabolites, and additional compounds may have contributed to additive exposure.

Third, the toxicological significance of several emerging mycotoxins detected, including AOH, AME, ENNs, BEA, and CPA, remains uncertain because tolerable daily intake values have not been established due to data gaps that should be addressed in future studies [1,127,135,169]. Furthermore, the additive and interactive effects of these and other mycotoxins detected in this study may be relevant in the context of chronic, possibly synergistic, exposure. However, evaluating mixture toxicity was beyond the scope of this study given the current limitations.

Fourth, estimated intakes were compared with available TDIs using a mean toddler body weight derived from U.S. growth charts to improve population relevance. However, this approach does not capture inter-individual variability arising from the cumulative effects of culturally specific dietary patterns, body weight differences, or differences in detoxification and metabolic systems. Therefore, it represents a conservative screening-level assessment rather than a comprehensive probabilistic exposure assessment, as defined by the EFSA [169].

Future research in the U.S. should focus on examining contamination rates in foods frequently consumed by children using more nationally representative surveillance, broader analytical coverage, and multistage stratified random sampling methods to ensure that the results can be generalized to U.S. children at large. Packaged cereals, pasta, pizza, dairy products, and even drinking water should be assessed to gauge the prevalence of mycotoxins in these commodities and to inform the development of a mitigation plan to protect vulnerable youth. Additionally, future research should compare infection rates between sustainably and organically grown foods and industrially farmed foods to better inform future mitigation efforts. Emerging interventions, such as the use of food- and human-safe microorganisms capable of degrading mycotoxins in agricultural and processing environments, warrant further investigation [162].

Finally, because dietary exposure typically involves mixtures, in vitro studies using human cell lines and relevant mycotoxin combinations may help clarify the synergistic, additive, and antagonistic effects of mycotoxins [36]. Direct exposure studies involving children are ethically impermissible. Nevertheless, mechanistic and dietary intervention studies aimed at reducing mycotoxin intake are viable. These studies would involve monitoring clinical and developmental outcomes, validated urinary and blood mycotoxin markers, and microbiome-related variables. Investigating these factors may also facilitate the evaluation of individual differences that more accurately represent real-world conditions and exposures, thereby elucidating their relationship with the development of chronic diseases. Collectively, these efforts are crucial for informing science-based risk assessments and regulatory decision-making aimed at protecting infants and young children from dietary mycotoxin exposure.

## 5. Conclusions

Although the global documentation of mycotoxin contamination in the food supply has increased, significant gaps persist in understanding dietary exposure and its potential public health implications in the U.S. This study identified a broad spectrum of both regulated and emerging mycotoxins in foods commonly consumed by children during a critical developmental period, such as grains, snack foods, pasta, first foods, and processed ingredients. Furthermore, some children may routinely exceed safe intake levels for multiple mycotoxins concurrently, potentially leading to adverse health effects.

We found that 95% of the foods tested contained at least one detected mycotoxin, and 87% contained at least one quantifiable mycotoxin. These findings highlight critical gaps in surveillance, toxicological characterization, and risk assessment in the pediatric population. Improved mitigation strategies across agricultural production, storage, and food processing are needed to reduce contamination at the source and throughout the supply chain. Expanded monitoring of foods marketed to infants and young children, refinement of regulatory frameworks, and greater integration of emerging mycotoxins into risk assessment processes are warranted. Addressing these challenges requires coordinated efforts among healthcare providers, scientists, public health professionals, farmers, food manufacturers, and policymakers to develop evidence-based strategies that more effectively protect infants and children from dietary mycotoxin exposure.

## Figures and Tables

**Table 1:** Set-up of the LC-MS/MS method and MS/MS parameters.

ESI Parameters	Value
Ion source	Turbo spray
Ionization mode	Positive and Negative
Acquisition mode	sMRM
Ion spray voltage	1700 V
Interface temperature	350 °C
Time	27 min
Curtain gas (CUR)	40 psi
Collision gas (CAD)	9 psi
Ion source gas 1 (GS 1)	40 psi
Ion source gas 2 (GS 2)	60 psi
Pause between mass ranges	5 ms

**Table 2:** Categories of foods sampled (*n* = 118).

Foods Tested	Number of Samples
Snack puffs, crackers, and biscuits (wheat, corn, oats, rice, and barley)	27
Processed cereal products (wheat, corn, oats, rice, and barley)	25
Pasta (wheat-based)	13
Misc. foods (plant-based formulas, beans, juice, fruit puree)	7
Wheat flour	10
Non-grain ingredients (almond flour, tapioca starch, arrowroot, coconut flour, chocolate powder)	15
Corn flour	21

**Table 3:** Numbers of samples contaminated with each mycotoxin.

Chemical Class/Structure	Analyte and Abbreviation	LOD (µg/kg)	Number of Detections	LOQ (µg/kg)	Number of Quantifications	Highest Amount (µg/kg)	Food Type with the Highest Amount	Median Amount (µg/kg)
Cyclic hexadepsipeptide	Beauvericin (BEA)	0.6	60	2	44	190.3	Breakfast cereals	8.2
	Enniatin A (EnnA)	0.6	45	2	23	95.6	Baby apple juice	14.9
	Enniatin A1 (EnnA1)	0.6	61	2	42	100.7	Baby apple juice	8.8
	Enniatin B (EnnB)	0.6	52	2	45	85.6	Pasta	10.4
	Enniatin B1 (EnnB1)	0.6	58	2	41	82.5	Baby apple juice	9.4
Dibenzopyrone	Alternariol (AOH)	0.6	15	2	13	28.4	Wheat flour	6.4
	Alternariol-Monomethyl Ether (AME)	0.6	38	2	35	36.4	Corn flour	3.8
Difuranocoumarin	Aflatoxin B1 (AFB1)	0.15	79	0.5	49	246.0	Corn flour	1.3
	Aflatoxin B2 (AFB2)	0.15	62	0.5	16	25.2	Corn flour	1.8
	Aflatoxin G1 (AFG1)	0.15	14	0.5	5	2.6	Corn flour	2.0
	Aflatoxin G2 (AFG2)	0.15	10	0.5	0	N/A	N/A	N/A
Difuranocoumarin xanthone precursor to aflatoxin	Sterigmatocystin (STC)	0.15	21	5	1	5.7	Corn flour	5.7
Indole alkaloid	Rocquefortine C (Rocq)	0.3	2	2	0	N/A	N/A	N/A
Indole-tetramic acid	Cyclopiazonic Acid (CPA)	0.15	10	2	10	3804.0	Corn flour	134.8
Isocoumarin + phenylalanine	Ochratoxin A (OTA)	0.3	19	1	6	2.5	Corn flour	1.2
Polyketide	Citrinin (CIT)	15	6	50	0	N/A	N/A	N/A
	Fumonisin B1 (FB1)	20	22	100	21	1300.0	Corn flour	300.0
	Fumonisin B2 (FB2)	10	17	100	15	500.0	Corn flour	100.0
	Fumonisin B3 (FB3)	10	5	100	5	200.0	Corn flour	100.0
	Griseofulvin (GRI)	0.6	2	2	0	N/A	Non-grain	N/A
Resorcyclic acid	Zearalenone (ZEA)	2	11	12.5	9	126.6	Corn flour	32.2
Resorcyclic acid reduced	a-Zearalenone (a-ZEA)	15	4	50	0	N/A	N/A	N/A
	b-Zearalenone (b-ZEA)	5	1	50	0	N/A	N/A	N/A
Trichothecene Type A	Diacetoxyscirpenol (DAS)	2	0	100	0	N/A	N/A	N/A
	HT-2 Toxin (HT-2)	0.8	13	5	8	71.2	Corn flour	9.5
	Neosolaniol (NEO)	0.1	13	20	0	N/A	N/A	N/A
	T-2 Toxin (T2)	0.1	9	5	4	49.6	Corn flour	34.7
Trichothecene Type B	15-Acetyl Deoxynivalenol (Ace-15)	2	13	100	5	700.0	Breakfast cereals	100.0
	3-Acetyl Deoxynivalenol (Ace-3)	3	3	100	1	100.0	Breakfast cereals	100.0
	Deepoxy-Deoxynivalenol (DOM)	4	10	20	10	93.4	Corn flour	48.8
	Deoxynivalenol (DON)	10	21	100	17	2500.0	Breakfast cereals	300.0
	Deoxynivalenol-3-Glucoside (DON-3-Glu)	5	6	20	3	585.3	Corn flour	24.6
	Fusarenon-x (FUS-X)	125	0	500	0	N/A	N/A	N/A
	Nivalenol (NIV)	150	4	500	0	N/A	Corn flour	N/A

Note: N/A = No Data. The limits of detection (LOD) could not be validated due to insufficient analytical precision at concentrations below the LOQ. Based on the conventional LC-MS/MS performance characteristics, the estimated LODs are expected to range between approximately one-third and one-fifth of the LOQ. The LOQ is below the guidance levels for detection. Although the LOD indicates that a toxin has been detected, the amount detected is unquantifiable. Therefore, it cannot be used in statistical risk assessments with any accuracy and was excluded from this type of evaluation.

**Table 4:** Number of mycotoxins quantified per food type by chemical structure.

Toxin/Food Category	Processed Cereals	Corn Flour	Non-Grain	Pasta	Snack Foods	Wheat Flour	First Foods	Total
Number of products tested	25	21	15	13	27	10	7	118
Products with one or more mycotoxins quantified	21	21	12	11	23	9	6	103
Cyclic hexadepsipeptide	35	16	21	25	66	19	13	195
Dibenzopyrone	7	6	0	8	8	13	5	47
Difuranocoumarin	5	40	10	1	7	5	2	70
Polyketide	8	24	0	0	2	8	0	42
Trichothecene	11	22	1	2	6	6	0	48
Other	9	11	0	3	1	2	0	26
Total	75	119	32	39	90	53	20	428

Number of mycotoxins quantified per food type by chemical structure across 118 tested products. Mycotoxins are grouped as follows: cyclic hexadepsipeptides (beauvericin [BEA], enniatin A [EnnA], enniatin A1 [EnnA1], enniatin B [EnnB], enniatin B1 [EnnB1]); dibenzopyrones (alternariol [AOH], alternariol monomethyl ether [AME]); difuranocoumarins (aflatoxin B1 [AFB1], aflatoxin B2 [AFB2], aflatoxin G1 [AFG1], aflatoxin G2 [AFG2]); polyketides (citrinin [CIT], fumonisin B1 [FB1], fumonisin B2 [FB2], fumonisin B3 [FB3], griseofulvin [GRI]); trichothecenes (type A: HT-2 toxin [HT-2], T-2 toxin [T2], diacetoxyscirpenol [DAS], neosolaniol [NEO]; type B: deoxynivalenol [DON], 3-acetyl deoxynivalenol [Ace-3], 15-acetyl deoxynivalenol [Ace-15], deoxynivalenol-3-glucoside [DON-3-Glu], deepoxy-deoxynivalenol [DOM], nivalenol [NIV], fusarenon-x [FUS-X]); and other (zearalenone [ZEA], α-zearalenone [α-ZEA], β-zearalenone [β-ZEA], sterigmatocystin [STC], Rocquefortine C [Rocq], cyclopiazonic acid [CPA], ochratoxin A [OTA]).

**Table 5:** Tolerable daily intake (TDI) toddler assessment by mycotoxin detected.

Mycotoxin	TDI	Highest Level and Fold Exceedance of TDI	Median Level and Fold Exceedance of TDI
* Aflatoxins (total AFB1, AFB2, AFG1, AFG2)	0.017 μg/kg bw/day	273.8 μg/kg = 38.7-fold higher	5.1 μg/kg = 0.72 of the TDI
Deoxynivalenol plus acetylated derivatives and masked forms	1 μg/kg bw/day	3199 μg/kg = 7.7-fold higher	300 μg/kg = 0.72 of the TDI
Nivalenol	1.2 μg/kg bw/day	N/A	N/A
Fumonisins (total FB1, FB2, and FB3)	1 μg/kg bw/day	1966 μg/kg = 4.7-fold higher	500 μg/kg = 1.2-fold higher
Ochratoxin A	0.120 μg/kg bw/week or 0.017/day	2.5 μg/kg = 0.69 of the TDI	1.2 μg/kg = 0.39 of the TDI
** T-2 Toxin and HT-2 Toxin combined	0.02 μg/kg bw/day	121.2 μg/kg = 14.5-fold higher	NA ***
T-2 Toxin	0.02 μg/kg bw/day	49.6 μg/kg = 6-fold higher	13.7 μg/kg = 1.6-fold higher
HT-2 Toxin	0.02 μg/kg bw/day	71.2 μg/kg = 8.5-fold higher	9.5 μg/kg = 1.1-fold higher
Zearalenone	0.25 μg/kg bw/day	126.6 μg/kg = 1.2-fold higher	32.2 μg/kg = 0.31 of the TDI

Note: Emerging mycotoxins without regulatory guidance and mycotoxin detections that were not quantifiable were not included in the table. Serving sizes for calculations were derived from the Feeding Infants and Toddlers Study (FITS) 2016 and the What We Eat in America/National Health and Nutrition Examination Survey (WWEIA/NHANES), with USDA Food Pattern recommendations. Calculations were based on using a 30 g serving size for all categories except OTA, which utilized a 70 g sample of wheat pasta; 12.5 kg body weight for toddler assessment using the following equations: Exposure ng/kg bw/day = Mycotoxin Contamination level (µg/kg) × Consumption data (g)/bw (kg) divided by 1000 for μg/kg bw/day, and Toddler Exposure = TDI (µg/kg bw day)/Actual intake (µg/kg bw day). * There is no established TDI for Aflatoxins, only a proposed value. ** T-2 and HT-2 Toxins are usually considered together in risk assessments but are separated here because they did not always occur together in samples. *** There was no median data available for T-2 and HT-2 toxins combined. Citations: [32,44,81,82,83,84,85,86].

## Data Availability

The majority of data obtained for this study are included in tables within the body of the paper. Raw data and supplemental data supporting the validation of the LC-MS/MS methods are available at: https://github.com/mlaviolet/mycotoxin (accessed date 10 July 2026).

## Supplementary Materials

The following supporting information can be downloaded at: https://www.mdpi.com/article/10.3390/ijerph23080949/s1, File S1: Mycotoxin LCMSMS Method Validation, File S2: LC-MSMS method uncertainty budget, File S3: Toddler foods PT data.

## Abbreviations

The following abbreviations are used in this manuscript:

AFB1	Aflatoxin B1
AFB2	Aflatoxin B2
AFG1	Aflatoxin G1
AFG2	Aflatoxin G2
AOH	Alternariol
AME	Alternariol monomethyl ether
ASD	Autism spectrum disorder
BEA	Beauvericin
bw	Body weight
CDC	Centers for Disease Control and Prevention
CPA	Cyclopiazonic acid
CIT	Citrinin
DAS	Diacetoxyscirpenol
DON	Deoxynivalenol
DON-3-Glu	Deoxynivalenol-3-glucoside
DOM	Deepoxy-deoxynivalenol
ENN	Enniatin
EnnA	Enniatin A
EnnA1	Enniatin A1
EnnB	Enniatin B
EnnB1	Enniatin B1
ESI	Electrospray ionization
FDA	Food and Drug Administration
FB1	Fumonisin B1
FB2	Fumonisin B2
FB3	Fumonisin B3
FUS-X	Fusarenon-X
GI	Gastrointestinal
GRI	Griseofulvin
HT-2	HT-2 toxin
IBD	Inflammatory bowel disease
LC–MS/MS	Liquid chromatography–tandem mass spectrometry
LOD	Limit of detection
LOQ	Limit of quantification
MRM	Multiple reaction monitoring
NEO	Neosolaniol
NIV	Nivalenol
OTA	Ochratoxin A
ppm	Parts per million
ROS	Reactive oxygen species
RSD	Relative standard deviation
sMRM	Scheduled multiple reaction monitoring
STC	Sterigmatocystin
T2	T-2 toxin
TDI	Tolerable daily intake
TTC	Threshold of Toxicological Concern
TWI	Tolerable weekly intake
ZEA	Zearalenone
α-ZEA	Alpha-zearalenol
β-ZEA	Beta-zearalenol

## Appendix A

**Table A1:** Foodborne mycotoxins tested.

Mycotoxin	Chemical Class/Structure	Foods Detected In and Producing Fungi	Known Human Effects and US FDA Monitoring in µg/kg	References
15-Acetyl Deoxynivalenol	Type B trichothecene (acetylated DON)	Cereals (wheat, barley, maize, oats); *Fusarium* spp.	GI upset and toxicity, nausea, vomiting, immunotoxicity, microbiome shifts, and possibly neurotoxicity. No US guidance or direct monitoring.	[13,15,17,114,115,116,117,170,171]
3-Acetyl Deoxynivalenol	Type B trichothecene	Cereals; *Fusarium* spp.	Similar to DON but lower toxicity; GI toxicity and gut barrier effects, microbiome shifts, and possibly neurotoxicity. No US guidance or direct monitoring.	[13,15,17,114,115,116,117,170,171]
Deoxynivalenol-3-Glucoside	Masked trichothecene (DON conjugate)	Processed cereals; *Fusarium* spp.	Hydrolyzed in the gut → DON exposure; GI toxicity, microbiome shifts. No US guidance or direct monitoring.	[13,15,17,114,115,116,117,170,171]
Deepoxy-Deoxynivalenol	Reduced trichothecene metabolite	Formed in ruminants and microbiota; cereals with *Fusarium* spp.	Less toxic metabolite of DON; lower GI toxicity. No US guidance or direct monitoring.	[172]
Deoxynivalenol (Vomitoxin)	Type B trichothecene	Wheat, maize, barley, oats; *Fusarium* spp.	Targets actively dividing cells, such as those lining the GI tract, skin, lymphoid, and erythroid cells. Causes immune system dysregulation. Categorized as a “ribotoxin” and protein synthesis inhibitor. Symptoms include anorexia, vomiting, abdominal pain, intestinal bleeding, fever, headache, immune modulation, microbiome shifts, and possibly neurotoxicity. Monitored in the US: 1000 µg/kg for adults in finished wheat products like flour, bran, and germ for adults, and 500 µg/kg in cereal-based foods for infants and children.	[13,15,17,106,113,114,115,116,117,170,171]
Nivalenol	Type B trichothecene	Barley, maize, wheat, rice; *Fusarium* spp.	GI toxicity, hematotoxicity, immunosuppression. No US guidance or direct monitoring.	[20,173,174,175,176]
T-2 Toxin	Type A trichothecene	Cereals, oats, maize; *Fusarium* spp.	Severe cytotoxicity. Targets actively dividing cells, such as those lining the GI tract, skin, lymphoid, and erythroid cells. Causes immune system dysregulation. Categorized as a “ribotoxin” and protein synthesis inhibitor. Symptoms include anorexia, vomiting, abdominal pain, intestinal bleeding, fever, headache, oral ulcers, petechia, hepatotoxicity, hematotoxicity, and bleeding. No US guidance or direct monitoring.	[14,20,56,82,103,176,177,178,179,180]
HT-2 Toxin	Type A trichothecene (T-2 metabolite)	Oats, wheat, maize; *Fusarium* spp.	Similar to T-2, GI toxicity, immunotoxicity, and hematotoxicity. No US guidance or direct monitoring.	[14,20,56,107,174,175,176,177,178,179]
Diacetoxyscirpenol	Type A trichothecene	Maize, barley; *Fusarium* spp.	GI toxicity, leukopenia, immunosuppression. No US guidance or direct monitoring.	[91]
Fusarenon-X	Type B trichothecene	Wheat, barley, maize; *Fusarium* spp.	GI effects, immunotoxicity, cytotoxicity. No US guidance or direct monitoring.	[91]
Neosolaniol	Type A trichothecene	Wheat, maize, barley; *Fusarium* spp.	Less toxic than T-2, GI toxicity, and immunotoxicity. No US guidance or direct monitoring.	[91]
Zearalenone	Resorcyclic acid lactone	Maize, wheat, barley; *Fusarium* spp.	An estrogenic hormone disruptor in some animals and possibly humans. Linked to hyperestrogenism, reproductive disorders, infertility, and perhaps early puberty in animals. No US guidance or direct monitoring.	[7,8,42,124,141,179,180]
α-Zearalenol	Reduced metabolite of ZEA	Formed in animals; cereals with *Fusarium* spp.	Similar to zearalenone, but with stronger estrogenic activity. No US guidance or direct monitoring.	[7,8,42,124,180]
β-Zearalenol	Reduced metabolite of ZEA	Same as above	Weaker estrogenic activity than the α-isomer. No US guidance or direct monitoring.	[7,8,42,124,180]
Fumonisin B1	Polyketide	Maize and other cereals, sorghum; *Fusarium* spp.	Disrupts sphingolipid metabolism in cell membranes and may be a causal factor in esophageal cancer and neural tube defects. Monitored in the US: 2000–4000 for FB1, FB2, and FB3 combined in foods consumed by humans.	[2,9,30,45,46,82,179]
Fumonisin B2	Polyketide	Maize and other cereals, sorghum; *Fusarium* spp.	Similar to FB1, possibly hepatotoxic. Monitored in the US: 2000–4000 for FB1, FB2, and FB3 combined in foods consumed by humans.	[2,9,30,45,46,82,179]
Fumonisin B3	Polyketide	Maize and other cereals; *Fusarium* spp.	Less potent than FB1, but with the same toxic profile. Monitored in the US: 2000–4000 for FB1, FB2, and FB3 combined in foods consumed by humans.	[2,9,30,45,46,82,179]
Enniatin A	Cyclic hexadepsipeptide	Grains, stored commodities, and dairy products; *Fusarium* spp.	Ionophoric, cytotoxic, adverse mitochondrial effects, and possibly hepatotoxic. No US guidance or direct monitoring.	[34,35,55,101,129,132]
Enniatin A1	Cyclic hexadepsipeptide	Grains, stored commodities, and dairy products; *Fusarium* spp.	Similar to Enniatin A and Enniatin B; it induces apoptosis, has mitochondrial effects, and may also exhibit hepatotoxicity. No US guidance or direct monitoring.	[34,35,63,90,101,129,133]
Enniatin B	Cyclic hexadepsipeptide	Grains, stored commodities, and dairy products; *Fusarium* spp.	Most abundant; cytotoxic, antimicrobial, mitochondrial effects, possibly hepatotoxic. No US guidance or direct monitoring.	[34,35,63,90,101,129,133]
Enniatin B1	Cyclic hexadepsipeptide	Grains, stored commodities, and dairy products; *Fusarium* spp.	Similar to Enniatin B; cytotoxic, antimicrobial, mitochondrial effects, possibly hepatotoxic. No US guidance or direct monitoring.	[34,35,63,90,101,129,133]
Beauvericin	Cyclic hexadepsipeptide	Cereals, maize, rice, stored commodities; *Fusarium* spp., *Bassiana beauveria*	Ionophoric, induces apoptosis, cardiotoxic in vitro, antimicrobial, and has mitochondrial effects. No US guidance or direct monitoring.	[35,63,90,101,129,132,133]
Ochratoxin A	Isocoumarin + phenylalanine	Cereals, coffee, dried fruit, cacao, wine; *Aspergillus* spp., *Penicillium* spp.	Nephrotoxic, immunotoxic, neurotoxic, and possibly carcinogenic. No US guidance or direct monitoring.	[9,25,30,122,179,181]
Citrinin	Polyketide	Cereals, rice, cheese, cacao; *Penicillium* spp., *Aspergillus* spp.	Nephrotoxic, mitochondrial dysfunction. No US guidance or direct monitoring.	[2,182]
Aflatoxin B1	Difuranocoumarin	Cereals, maize, peanuts, tree nuts; *Aspergillus* spp.	Hepatotoxic and hepatocarcinogenic (Group 1), immunosuppressive, and GI toxicity. Monitored in the US: 20 for total (AB1, AB2, AG1, and AG2 combined).	[2,31,32,33,81,96,97,98,99,141,183]
Aflatoxin B2	Difuranocoumarin	Same foods; *Aspergillus* spp.	Similar to B1, less potent. Monitored in the US: 20 for total (AB1, AB2, AG1, and AG2 combined).	[2,31,32,33,81,96,97,98,99,141,183]
Aflatoxin G1	Difuranocoumarin	Maize, nuts; *Aspergillus* spp.	Carcinogenic and hepatotoxic. Monitored in the US: 20 for total (AB1, AB2, AG1, and AG2 combined).	[2,31,32,33,81,96,97,98,99,141,183]
Aflatoxin G2	Difuranocoumarin	Same foods; *Aspergillus* spp.	Lower toxicity than G1, carcinogenic, and hepatotoxic. Monitored in the US: 20 for total (AB1, AB2, AG1, and AG2 combined).	[2,31,32,33,81,96,97,98,99,141,183]
Sterigmatocystin	Xanthone precursor of aflatoxin	Cereals, cheese; *Aspergillus* spp.	Hepatotoxic, probable carcinogen 2B. No US guidance or direct monitoring.	[105]
Cyclopiazonic Acid	Indole-tetramic acid	Maize, peanuts, milk, cheese, meat products, and eggs; *Aspergillus* spp., *Penicillium* spp.	Muscle tremors, neurotoxin, and GI toxicity. Possibly responsible for Kodua poisoning in humans. No US guidance or direct monitoring.	[26,27]
Alternariol	Dibenzopyrone	Tomatoes, citrus, cereals; *Alternaria* spp.	Carcinogenic, nephrotoxic, hepatotoxic, and immunotoxic in animals at low µg/kg/day in repeated doses. Has demonstrated antibiotic/antibacterial activity (e.g., against Staphylococcus aureus and Candida albicans), cholinesterase inhibition, and some antioxidant activity in specific assays. No US guidance or direct monitoring.	[37,40,41]
Alternariol-Monomethyl Ether	Dibenzopyrone	Tomatoes, cereals; *Alternaria* spp.	Genotoxic in vitro and shows toxicity to liver, kidney, spleen, and immune function in animals at low µg/kg/day in repeated doses. No US guidance or direct monitoring.	[37,40,41]
Roquefortine C	Indole alkaloid	Cheese, cereals; *Penicillium* spp.	Neurotoxic, with increased generation of reactive oxygen species (ROS), and convulsant activity in animals. No US guidance or direct monitoring.	[184]
Griseofulvin	Polyketide secondary metabolite	Cereals, chocolate; *Penicillium* spp.	Hepatocarcinogen, GI disturbances, and allergic reactions. Used in medicine as an antifungal medication. No US guidance or direct monitoring.	[185,186]

## Appendix B

**Table A2:** Exposure limits set in Europe, China, and the U.S. FDA for Mycotoxins.

Mycotoxin	Food Commodity	U.S. FDA (µg/kg)	Europe (µg/kg)	China (µg/kg)
Aflatoxin B1, B2, G1, G2	Maize (corn), wheat, rice, peanut, sorghum, pistachio, almond, ground nuts, tree nuts, figs, dried fruit, cottonseed, spices, cocoa	20 for total	2–12 for B1; 4–15 for total	5–20 for B1; 0.5 in foods intended for infants
Aflatoxin M1	Milk, milk products, cheeses	0.5 in milk	0.05 in milk; 0.025 in infant formula and infant milk	0.2–0.5 in milk; 0.5 in infant formula
Ochratoxin A	Cereals, dried vine fruit, wine, grapes, coffee, cocoa, cheese	Not set; monitored by FDA	2–10; 0.5 in products for infants	5–10
Citrinin	Food supplements based on rice fermented with Monascus purpureus (red yeast rice)	Not set	100	Not set
Fumonisins B1, B2, and B3	Maize and maize products, cereal grains, sorghum, and asparagus	2000–4000	200–1000	1000–4000 for adults; 200 for infants and small children
Zearalenone	Cereals, cereal products, maize, wheat, barley, milk, corn oil	Not set; monitored by FDA.	20–100	60
Deoxynivalenol	Cereals, cereal products, maize, wheat, barley, rye, buckwheat, oats, millet, triticale, rice, sorghum, alcoholic beverages from cereal grains	1000 in finished wheat products like flour, bran, and germ; 500 in cereal-based foods for infants and children	200–500 in processed grains; 200 in products for infants and young children	750–2000 in processed grains and adult foods; 200 in products for infants and young children
Nivalenol	Cereals, cereal products, maize, wheat, barley, rye, buckwheat, oats, millet, triticale, rice, sorghum, alcoholic beverages from cereal grains	Not set	No harmonized EU maximum level is established. Monitoring and risk assessment continue	Not set
T-2 and HT-2 Toxin	Cereals, cereal products, bakery products, maize, wheat, barley, rye, buckwheat, oats, millet, triticale, rice	Not set; monitored by FDA	Adults 20–100; infants and children 10	Not set
Patulin	Apples (rotten), apple juice, and concentrate (can also occur in other fruits, dried fruits, and juices)	50	10 for infants; up to 50 for adults	50

References: Regulatory limits were compiled from U.S. Food and Drug Administration (FDA) action levels, advisory levels, and guidance documents, Commission Regulation (EU) 2023/915 and related European Commission contaminant legislation, and the Chinese National Food Safety Standard GB 2761-2017 and associated government notifications [68,104,128,187,188,189].
