# Supplementary material for: Occurrence and Co-Occurrence of Regulated and Emerging Mycotoxins in Foods Marketed to U.S. Toddlers
Source: Int J Environ Res Public Health. 2026 Jul 23;23(8):949. doi: 10.3390/ijerph23080949 (PMC13512329; doi:10.3390/ijerph23080949)

Mycotoxin LCMSMS Method Validation

Accuracy	Corn/3 Acetyl DON	Corn/15 Acetyl DON	Corn/DON	Corn/T2	Corn/HT2	Corn/Total Fumonisin
	MTC-9999E	MTC-9999E	MTC-9999E	MTC-9999E	MTC-9999E	F435
1	0.13	0.09	2.13	231.1	355.9	3.8
2	0.10	0.10	2.12	254.5	345.1	3.86
3	0.12	0.09	2.11	230.2	336	3.79
4	0.12	0.09	2.06	250.6	341.5	3.62
5	0.12	0.11	2.16	228	305.7	3.8
6	0.13	0.09	2.27	250.2	288.8	3.91
7	0.10	0.08	2.24	241.2	298.5	3.53
Mean	0.12	0.09	2.16	240.8	324.5	3.76

Detection Limit	Corn/3 Acetyl DON	Corn/15 Acetyl DON	Corn/DON	Corn/T2	Corn/HT2	Corn/Fumonisin B1
Actual	0.003	0.002	0.01 ppm	0.13 ppb	0.8 ppb	0.02 ppm
Limit of Quantification						
Actual	0.01 ppm	0.006 ppm	0.03 ppm	0.40 ppb	2.5 ppb	0.06 ppm
Reported	0.1 ppm	0.1 ppm	0.1 ppm	5 ppb	5 ppb	0.1 ppm

% RSD	9.26	10.24	3.46	4.62	8.09	3.59
% Accuracy	101.1	103.2	82.9	91.3	91.3	94.2
Selectivity	pass	pass	pass	pass	pass	pass

Linearity	R^2	Range
3-Acetyl DON	0.9999	0.01ppm - 3.0ppm
15-Acetyl DON	0.9923	0.1ppm - 5.0ppm
DON	0.9997	0.03ppm - 10.0ppm
T2	0.9988	5ppb - 2000ppb
HT2	0.9998	5ppb - 2000ppb
Fumonisin B1	0.9994	0.03ppm - 10.0ppm
Fumonisin B2	0.9996	0.05ppm - 5.0ppm
Fumonisin B3	0.9997	0.01ppm - 2.5ppm

Acceptability

**RSD**	< 15%
**%Accuracy**	70%-110%
**Linearity**	$R^2 > 0.99$
**Detection Limit**	< reported detection limit

Mycotoxin LCMSMS Method Validation

Accuracy	Corn/Ochratoxin	Corn/Zearalenone	Corn/Total Aflatoxin
	MTC-9999E	MTC-9999E	MTC-9999E
1	6.00	378.3	25.48
2	5.69	317.9	25.38
3	5.53	324.8	26.38
4	5.11	335.1	25.55
5	5.71	349.9	26.65
6	5.31	367.9	28.06
7	5.59	329.2	28.89
Mean	5.56	343.3	26.6

Detection Limit	Corn/Ochratoxin	Corn/Zearalenone	Corn/Aflatoxin B1
Actual	0.33 ppb	2.65 ppb	0.18 ppb
Limit of Quantification			
Actual	0.99 ppb	7.96 ppb	0.54 ppb
Reported	1 ppb	10 ppb	1 ppb

% RSD	5.19	6.65	5.15
% Accuracy	97.6	97.5	90.3
Selectivity	pass	pass	pass

Linearity	R^2	Range
Ochratoxin	0.9986	0.125ppb - 50ppb
Zearalenone	0.9978	12.5ppb-5000ppb
Aflatoxin B1	0.9992	0.25ppb - 100ppb
Aflatoxin B2	0.9980	0.0625ppb - 25ppb
Aflatoxin G1	0.9999	0.25ppb - 100ppb
Aflatoxin G2	0.9994	0.25ppb - 25ppb

Acceptability

**RSD**	< 15%
**%Accuracy**	70%-110%
**Linearity**	$R^2 > 0.99$
**Detection Limit**	< reported detection limit

LC-MS/MS Method Validation

Different Mid Level Spikes

Accuracy	AFB1	AFB2	AFG1	AFG2	DON	HT2	T2	ZONE
**Spike Concentration**	**35.25**	**9.015**	**34.5**	**9.015**	**3.795**	**724.5**	**721.5**	**729**
**1**	34.86	7.6	32.1	7.70	3.92	743.1	697.29	794.56
**2**	35.32	8.18	32.3	8.04	3.82	641.47	666.63	792.39
**3**	35.45	8.16	32.18	8.03	4.02	727.23	678.39	779.52
**4**	35.12	7.84	31.52	7.82	3.75	669.53	659.07	759.59
**5**	35.1	7.69	31.25	7.43	3.82	776.04	669.47	738.16
**6**	34.86	8.12	32.63	7.77	3.96	675.54	675.21	733.62
**7**	35.28	8.05	31.78	7.96	3.82	691.59	669.77	780.36
**Mean**	35.1	7.9	32.0	7.8	3.9	703.5	673.7	768.3
**% RSD**	0.64	2.99	1.49	2.77	2.47	6.70	1.80	3.24
**% Accuracy**	99.69	88.17	92.65	86.76	102.05	97.10	93.37	105.39

Same Mid Level Spikes

Accuracy	AFB1	AFB2	AFG1	AFG2	DON	HT2	T2	ZONE
**Spike Concentration**	**35.25**	**9.015**	**34.5**	**9.015**	**3.795**	**724.5**	**721.5**	**729**
**1**	35	8.05	32.57	7.94	3.84	611.88	633.72	732.02
**2**	33.92	7.94	30.09	7.49	3.74	609.85	635.5	764.9
**3**	33.67	7.76	30.47	7.84	3.60	616.43	634.5	752.51
**4**	34.96	8.16	32.38	7.91	3.78	649.81	681.12	742.65
**5**	34.38	8.22	31.29	7.72	3.95	657.29	653.96	760.36
**6**	34.49	7.88	31.49	7.57	3.87	636.07	657.55	720.36
**7**	34.02	8.48	32.95	7.64	3.66	645.46	646.31	719.1
**Mean**	34.3	8.1	31.6	7.7	3.8	632.4	649.0	741.7
**% RSD**	1.49	2.99	3.43	2.24	3.22	3.09	2.64	2.50
**% Accuracy**	97.44	89.52	91.61	85.75	99.53	87.29	89.94	101.74

Low Level Spikes

Accuracy	AFB1	AFB2	AFG1	AFG2	DON	HT2	T2	ZONE
**Spike Concentration**	**2.35**	**0.601**	**2.3**	**0.562**	**0.253**	**48.3**	**48.1**	**48.6**
**1**	2.37	0.59	2.13	0.51	0.26	45.30	46.43	54.93
**2**	2.33	0.53	2.39	0.33	0.28	44.96	47.18	52.25
**3**	2.34	0.52	2.31	0.41	0.25	42.67	45.80	51.26
**4**	2.26	0.54	2.18	0.60	0.26	44.65	44.87	49.41
**5**	2.34	0.56	2.06	0.66	0.28	46.67	47.07	57.09
**6**	2.28	0.54	2.15	0.42	0.27	46.27	45.69	57.86
**7**	2.31	0.62	2.22	0.48	0.23	44.52	47.01	52.15
**Mean**	2.3	0.6	2.2	0.5	0.3	45.0	46.3	53.6
**% RSD**	1.64	6.45	5.09	23.46	6.78	2.91	1.88	5.86
**% Accuracy**	98.66	92.70	95.90	86.68	103.33	93.18	96.24	110.21

Different Low Level Spikes

Accuracy	AFB1	AFB2	AFG1	AFG2	DON	HT2	T2	ZONE
**Spike Concentration**	**2.35**	**0.601**	**2.3**	**0.562**	**0.253**	**48.3**	**48.1**	**48.6**
**1**	2.35	0.57	2.26	0.43	0.28	48.28	46.61	55.4
**2**	2.18	0.54	2.08	0.42	0.24	42.59	45.39	53
**3**	2.2	0.55	2.10	0.42	0.22	37.14	43.68	49.6
**4**	2.25	0.58	2.12	0.55	0.26	45.69	44.94	51.02
**5**	2.22	0.56	2.01	0.49	0.26	49.22	45.33	50.88
**6**	2.21	0.55	2.26	0.49	0.25	43.07	45.32	50.93
**7**	2.32	0.55	2.21	0.38	0.24	43.49	44.40	50.41
**Mean**	2.2	0.6	2.1	0.5	0.3	44.2	45.1	51.6
**% RSD**	2.86	2.48	4.48	12.76	7.66	9.15	2.02	3.80
**% Accuracy**	95.62	92.70	93.42	80.83	98.81	91.54	93.75	106.18

	AFB1	AFB2	AFG1	AFG2	DON	HT2	T2	ZONE
**Detection Limit**	0.25ppb	0.25ppb	0.9ppb	0.25ppb	0.1ppm	5ppb	5ppb	5ppb
**Reported Level**	1ppb	1ppb	1ppb	1ppb	0.1ppm	5ppb	5ppb	12.5ppb
**Selectivity (Pass/Fail)**	Pass	Pass	Pass	Pass	Pass	Pass	Pass	Pass

Linearity	R or R^2 Value	Range / ppm
AFB1	0.99971	0.25-100ppb
AFB2	0.99906	0.25-25ppb
AFG1	0.99929	0.9-100ppb
AFG2	0.99975	0.25-25ppb
DON	0.99965	0.1-10ppm
HT2	0.99972	5-2000ppb
T2	0.99989	5-2000ppb
ZONE	0.99977	5-2000ppb

Acceptability

**RSD**	< 20%
**%Accuracy**	80%-120%
**Linearity**	HPLC: $R^2 > 0.99$
	LCMSMS: $R > 0.99$
**Detection Limit**	< reported detection limit

LC-MS/MS Method Validation

Different Mid Level Spikes

Accuracy	FB1	FB2	FB3	OTA
**Spike Concentration**	**3.57**	**1.638**	**0.791**	**30**
**1**	3.95	1.8553	0.8216	29.56
**2**	3.86	1.8448	0.8149	31.12
**3**	4.00	1.8618	0.8551	31.12
**4**	3.95	1.8236	0.8372	31.57
**5**	3.95	1.8393	0.841	31.01
**6**	3.96	1.827	0.8332	31.74
**7**	3.95	1.7936	0.8434	30.16
**Mean**	3.9	1.8	0.8	30.9
**% RSD**	1.01	1.25	1.62	2.51
**% Accuracy**	110.48	112.03	105.59	102.99

Same Mid Level Spikes

Accuracy	FB1	FB2	FB3	OTA
**Spike Concentration**	**3.57**	**1.638**	**0.791**	**30**
**1**	3.9053	1.7638	0.8195	30.06
**2**	3.9566	1.8288	0.8235	30.44
**3**	3.9389	1.8776	0.846	31.28
**4**	4.0225	1.856	0.848	32.25
**5**	3.9273	1.8744	0.8303	33.03
**6**	4.0311	1.8568	0.8584	31.42
**7**	4.0239	1.8746	0.8548	30.51
**Mean**	4.0	1.8	0.8	31.3
**% RSD**	1.32	2.20	1.85	3.41
**% Accuracy**	111.27	112.79	106.20	104.29

Low Level Spikes

Accuracy	FB1	FB2	FB3	OTA
**Spike Concentration**	**0.51**	**0.234**	**0.113**	**5**
**1**	0.60	0.28	0.11	4.76
**2**	0.61	0.28	0.11	4.08
**3**	0.58	0.27	0.10	5.92
**4**	0.55	0.27	0.10	5.42
**5**	0.57	0.27	0.10	5.62
**6**	0.58	0.28	0.11	4.37
**7**	0.58	0.28	0.10	5.36
**Mean**	0.6	0.3	0.1	5.1
**% RSD**	3.56	2.06	4.37	13.45
**% Accuracy**	113.85	117.55	93.44	101.50

Different Low Level Spikes

Accuracy	FB1	FB2	FB3	OTA
**Spike Concentration**	**0.51**	**0.234**	**0.113**	**5**
**1**	0.62	0.288	0.117	4.47
**2**	0.61	0.288	0.111	4.96
**3**	0.62	0.280	0.116	5.15
**4**	0.61	0.284	0.109	4.82
**5**	0.61	0.289	0.113	5.76
**6**	0.61	0.288	0.114	5.33
**7**	0.62	0.286	0.115	4.87
**Mean**	0.6	0.3	0.1	5.1
**% RSD**	0.66	1.11	2.47	8.15
**% Accuracy**	120.57	122.28	100.43	101.01

	FB1	FB2	FB3	OTA
**Detection Limit**	0.25ppb	0.25ppb	0.9ppb	0.25ppb
**Reported Level**	1ppb	1ppb	1ppb	1ppb
**Selectivity (Pass/Fail)**	Pass	Pass	Pass	Pass

Linearity	R or R^2 Value	Range / ppm
FB1	0.99954	0.025-10ppm
FB2	0.99949	0.0125-5ppm
FB3	0.99985	0.00625-2.5ppm
OTA	0.99951	0.5-50ppb

Acceptability

**RSD**	< 15
**%Accuracy**	80%-120%
**Linearity**	HPLC: $R^2 > 0.99$
	LCMSMS: $R > 0.99$
**Detection Limit**	eported detection limit

MultiQuant - [[MQ4] Results Table (Untitled)]

File Edit Process Window Help

Mycotoxins\2017

Components & Groups IS

All Components

3 Acetyl DON Group
AFB1 Group
AFB2 Group
AFG1 Group
AFG2 Group
Citrinin Group
DAS Group
DON Group
FB1 Group
FB2 Group
FX Group
HT2 Group
NEOS Group
NIV Group
OTA Group
Sterig Group
ZONE Group
FB3 Group
15-AC Group
T2 Group
CIT-neg Group
DON-neg Group
NIV-neg Group
ZON-neg Group
3AC-neg Group

3 Acetyl DON.1
3 Acetyl DON.2
AFB1.1
AFB1.2
AFB2.1
AFB2.2
AFG1.1
AFG1.2
AFG2.1
AFG2.2
Citrinin.1
Citrinin.2
DAS.1
DAS.2
DON.1
DON.2
FB1.1
FB1.2
FB2.1
FB2.2
FX.1
FX.2
HT2.1
HT2.2
NEOS.1
NEOS.2
NIV.1
NIV.2
OTA.1
OTA.2
Sterig.1
Sterig.2
ZONE.1
ZONE.2
FB3.1
FB3.3
15-AC.4
15-AC.5
T2.1
T2.2
CIT-neg.1
CIT-neg.2
DON-neg.1

All Sample Types

Index	Sample Name	Sample Type	Component Name	Component Group Name	Outlier Reasons	Actual Concentration	Area	Height	Retention Time	Width at 50%	Signal / Noise	Used	Calculated Concentration	Accuracy	Ion Ratio	Expected Ion Ratio
33	Neat Standard_1	Standard	ZONE.1	ZONE	Ion Ratio	0.0125	345460.019	8.661e4	9.97	0.06	221.2	☐	0.011	88.83	0.0063	0.5103
85	Neat Standard_2	Standard	ZONE.1	ZONE		0.0250	660435.098	1.682e5	9.95	0.06	366.2	☑	0.023	92.03	0.4824	0.5103
137	Neat Standard_3	Standard	ZONE.1	ZONE		0.0500	1305850.0...	3.435e5	9.96	0.06	578.0	☑	0.047	94.79	0.4869	0.5103
189	Neat Standard_4	Standard	ZONE.1	ZONE		0.1250	3419123.0...	8.938e5	9.96	0.06	1049.4	☑	0.127	101.80	0.4942	0.5103
241	Neat Standard_5	Standard	ZONE.1	ZONE		0.2500	6746262.1...	1.780e6	9.97	0.06	1050.9	☑	0.253	101.19	0.4944	0.5103
293	Neat Standard_6	Standard	ZONE.1	ZONE		0.5000	13395960...	3.531e6	9.96	0.06	866.5	☑	0.504	100.85	0.4933	0.5103
345	Neat Standard_7	Standard	ZONE.1	ZONE		1.2500	36283240...	9.729e6	9.98	0.06	1034.8	☑	1.369	109.53	0.4990	0.5103
397	Neat Standard_8	Standard	ZONE.1	ZONE		2.5000	69246924...	1.707e7	9.98	0.06	976.2	☑	2.615	104.59	0.5014	0.5103
449	Neat Standard_9	Standard	ZONE.1	ZONE		5.0000	12605307...	3.107e7	9.98	0.06	1017.5	☑	4.761	95.23	0.5304	0.5103
501	Neat Standard_10	Standard	ZONE.1	ZONE		12.5000	26629553...	5.750e7	9.97	0.07	737.2	☐	10.061	80.49	0.5666	0.5103
553	Neat Standard_11	Standard	ZONE.1	ZONE	Accurac...	25.0000	37568538...	7.256e7	9.97	0.08	621.7	☐	14.194	56.78	0.6397	0.5103
605	Neat Standard_12	Standard	ZONE.1	ZONE	Accurac...	50.0000	48834183...	7.811e7	9.97	0.10	444.2	☐	18.451	36.90	0.7204	0.5103

Regression

Calibration for ZONE.1: y = 2.64637e7 x + 5.15992e4 (r = 0.99840) (weighting: 1 / x)

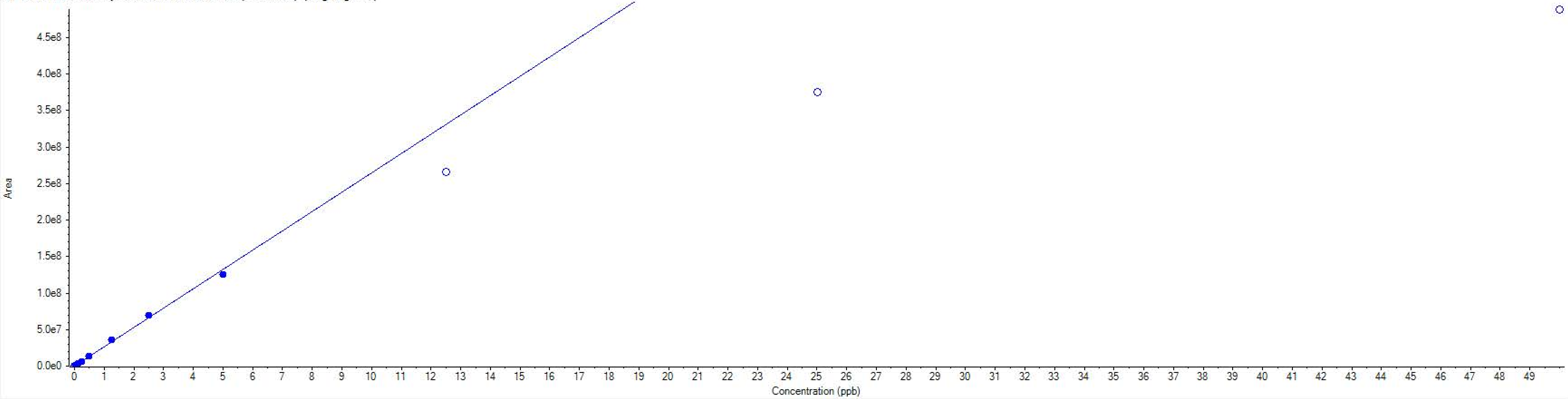

MultiQuant - [[MQ4] Results Table (Untitled)]

File Edit Process Window Help

Mycotoxins\2017

Components & Groups IS

Citrinin Group
DAS Group
DON Group
FB1 Group
FB2 Group
FX Group
HT2 Group
NEOS Group
NIV Group
OTA Group
Sterig Group
ZONE Group
FB3 Group
15-AC Group
T2 Group
CIT-neg Group
DON-neg Group
NIV-neg Group
ZON-neg Group
3AC-neg Group

3 Acetyl DON.1
3 Acetyl DON.2
AFB1.1
AFB1.2
AFB2.1
AFB2.2
AFG1.1
AFG1.2
AFG2.1
AFG2.2
Citrinin.1
Citrinin.2
DAS.1
DAS.2
DON.1
DON.2
FB1.1
FB1.2
FB2.1
FB2.2
FX.1
FX.2
HT2.1
HT2.2
NEOS.1
NEOS.2
NIV.1
NIV.2
OTA.1
OTA.2
Sterig.1
Sterig.2
ZONE.1
ZONE.2
FB3.1
FB3.3
15-AC.4
15-AC.5
T2.1
T2.2
CIT-neg.1
CIT-neg.2
DON-neg.1
DON-neg.2
NIV-neg.1
NIV-neg.2
NIV-neg.3
NIV-neg.4
ZON-neg.1
ZON-neg.2

All Sample Types

Index	Sample Name	Sample Type	Component Name	Component Group Name	Outlier Reasons	Actual Concentration	Area	Height	Retention Time	Width at 50%	Signal / Noise	Used	Calculated Concentration	Accuracy	Ion Ratio	Expected Ion Ratio
45	Neat Standard_1	Standard	NIV-neg.1	NIV-neg	Accurac...	0.0125	1581.490	5.744e2	4.01	0.04	15.5	☐	0.126	1011.42	1.4382	0.6930
97	Neat Standard_2	Standard	NIV-neg.1	NIV-neg	Accurac...	0.0250	5451.165	1.679e3	4.00	0.05	41.2	☐	0.139	557.04	0.8255	0.6930
149	Neat Standard_3	Standard	NIV-neg.1	NIV-neg	Accurac...	0.0500	7351.409	2.322e3	4.00	0.05	50.2	☐	0.146	291.12	1.3416	0.6930
201	Neat Standard_4	Standard	NIV-neg.1	NIV-neg	Accuracy	0.1250	24394.018	8.433e3	4.01	0.04	117.1	☑	0.202	161.66	0.6253	0.6930
253	Neat Standard_5	Standard	NIV-neg.1	NIV-neg		0.2500	44273.455	1.433e4	4.00	0.05	131.0	☑	0.268	107.20	0.6719	0.6930
305	Neat Standard_6	Standard	NIV-neg.1	NIV-neg		0.5000	91327.846	2.909e4	4.00	0.05	191.3	☑	0.424	84.81	0.6863	0.6930
357	Neat Standard_7	Standard	NIV-neg.1	NIV-neg	Accuracy	1.2500	238074.253	7.890e4	4.02	0.05	210.6	☑	0.911	72.85	0.7213	0.6930
409	Neat Standard_8	Standard	NIV-neg.1	NIV-neg	Accuracy	2.5000	551128.599	1.874e5	4.02	0.04	237.3	☑	1.949	77.95	0.6650	0.6930
461	Neat Standard_9	Standard	NIV-neg.1	NIV-neg		5.0000	1253617.0...	4.242e5	4.04	0.04	200.3	☑	4.278	85.57	0.6927	0.6930
513	Neat Standard_10	Standard	NIV-neg.1	NIV-neg		12.5000	4003161.8...	1.322e6	4.01	0.05	242.8	☑	13.396	107.17	0.6872	0.6930
565	Neat Standard_11	Standard	NIV-neg.1	NIV-neg		25.0000	7712484.6...	2.377e6	4.01	0.05	229.3	☑	25.697	102.79	0.7089	0.6930
617	Neat Standard_12	Standard	NIV-neg.1	NIV-neg		50.0000	14479324...	4.277e6	4.02	0.05	195.4	☐	48.137	96.27	0.7230	0.6930

Regression

Calibration for NIV-neg.1: y = 3.01556e5 x + -3.65436e4 (r = 0.99467) (weighting: 1 / x)

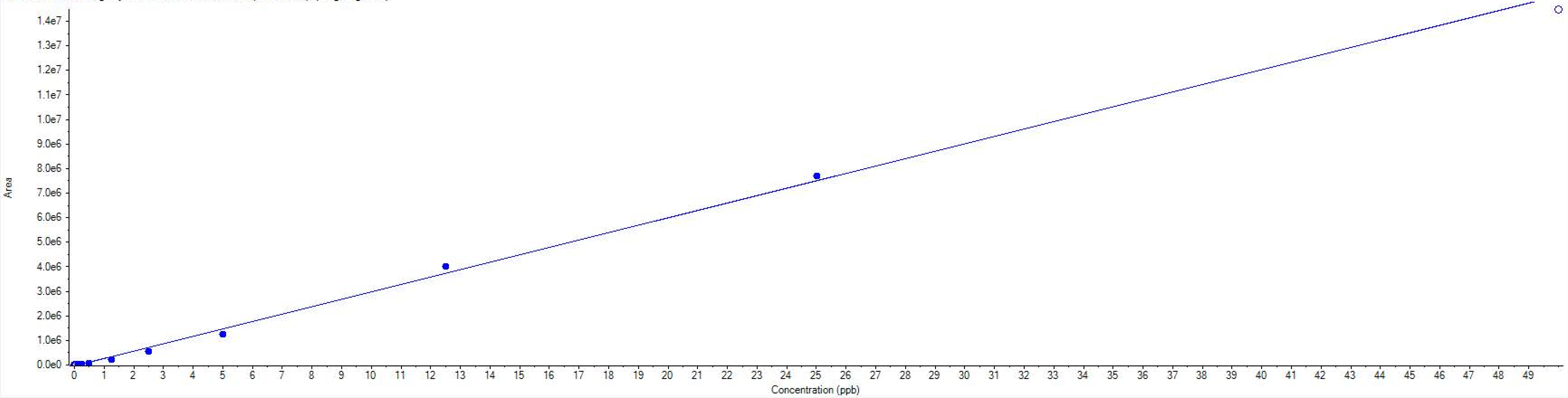

MultiQuant - [[MQ4] Results Table (Untitled)]

File Edit Process Window Help

Mycotoxins\2017

Components & Groups IS

All Components

3 Acetyl DON Group
AFB1 Group
AFB2 Group
AFG1 Group
AFG2 Group
Citrinin Group
DAS Group
DON Group
FB1 Group
FB2 Group
FX Group
HT2 Group
NEOS Group
NIV Group
OTA Group
Sterig Group
ZONE Group
FB3 Group
15-AC Group
T2 Group
CIT-neg Group
DON-neg Group
NIV-neg Group
ZON-neg Group
3AC-neg Group

3 Acetyl DON.1
3 Acetyl DON.2
AFB1.1
AFB1.2
AFB2.1
AFB2.2
AFG1.1
AFG1.2
AFG2.1
AFG2.2
Citrinin.1
Citrinin.2
DAS.1
DAS.2
DON.1
DON.2
FB1.1
FB1.2
FB2.1
FB2.2
FX.1
FX.2
HT2.1
HT2.2
NEOS.1
NEOS.2
NIV.1
NIV.2
OTA.1
OTA.2
Sterig.1
Sterig.2
ZONE.1
ZONE.2
FB3.1
FB3.3
15-AC.4
15-AC.5
T2.1
T2.2
CIT-neg.1
CIT-neg.2
DON-neg.1
[illegible]

All Sample Types

Index	Sample Name	Sample Type	Component Name	Component Group Name	Outlier Reasons	Actual Concentration	Area	Height	Retention Time	Width at 50%	Signal / Noise	Used	Calculated Concentration	Accuracy	Ion Ratio	Expected Ion Ratio
29	Neat Standard_1	Standard	OTA.1	OTA	Accurac...	0.0063	1036.259	5.232e2	9.82	0.03	4.8	☐	< 0	N/A	N/A	0.1997
81	Neat Standard_2	Standard	OTA.1	OTA	Accurac...	0.0125	4313.878	1.335e3	10.01	0.04	12.7	☐	< 0	N/A	N/A	0.1997
133	Neat Standard_3	Standard	OTA.1	OTA	Accurac...	0.0250	15939.579	3.121e3	10.02	0.06	35.0	☐	< 0	N/A	0.0816	0.1997
185	Neat Standard_4	Standard	OTA.1	OTA	Accuracy	0.0625	36456.889	7.050e3	10.03	0.06	70.2	☑	0.129	205.83	0.1668	0.1997
237	Neat Standard_5	Standard	OTA.1	OTA	Accurac...	1.2500	72795.077	1.308e4	10.03	0.07	110.8	☑	0.640	51.21	0.0790	0.1997
289	Neat Standard_6	Standard	OTA.1	OTA	Accuracy	2.5000	145020.516	2.539e4	10.02	0.07	153.0	☑	1.657	66.27	0.1891	0.1997
341	Neat Standard_7	Standard	OTA.1	OTA		6.2500	408386.210	7.465e4	10.04	0.06	278.6	☑	5.364	85.83	0.1795	0.1997
393	Neat Standard_8	Standard	OTA.1	OTA		12.5000	822397.210	1.409e5	10.04	0.07	324.2	☑	11.192	89.53	0.2136	0.1997
445	Neat Standard_9	Standard	OTA.1	OTA		25.0000	1672864.7...	2.864e5	10.04	0.07	398.3	☑	23.163	92.65	0.2060	0.1997
497	Neat Standard_10	Standard	OTA.1	OTA		62.5000	4852253.2...	8.294e5	10.03	0.07	415.0	☑	67.918	108.67	0.2078	0.1997
549	Neat Standard_11	Standard	OTA.1	OTA		125.0000	10375637....	1.787e6	10.03	0.07	384.6	☐	145.667	116.53	0.2072	0.1997
601	Neat Standard_12	Standard	OTA.1	OTA	Accuracy	250.0000	22055287....	3.907e6	10.03	0.07	378.8	☐	310.074	124.03	0.2022	0.1997

Regression

Calibration for OTA.1: y = 7.10410e4 x + 27317.77179 (r = 0.99299) (weighting: 1 / x)

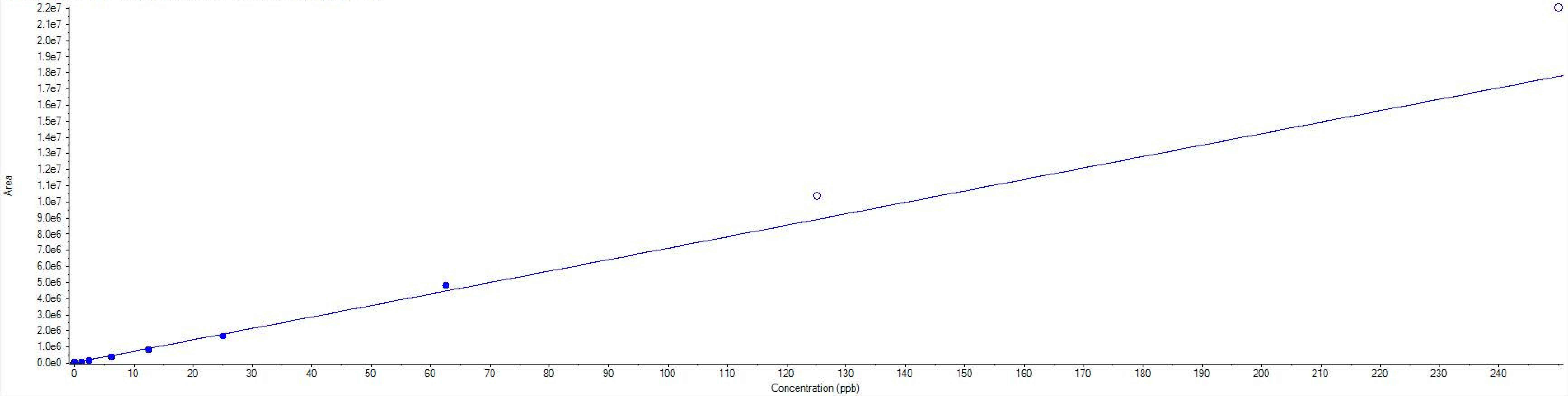

MultiQuant - [[MQ4] Results Table (Untitled)]

File Edit Process Window Help

Mycotoxins\2017

Components & Groups IS

All Components

3 Acetyl DON Group
AFB1 Group
AFB2 Group
AFG1 Group
AFG2 Group
Citrinin Group
DAS Group
DON Group
FB1 Group
FB2 Group
FX Group
HT2 Group
NEOS Group
NIV Group
OTA Group
Sterig Group
ZONE Group
FB3 Group
15-AC Group
T2 Group
CIT-neg Group
DON-neg Group
NIV-neg Group
ZON-neg Group
3AC-neg Group

3 Acetyl DON.1
3 Acetyl DON.2
AFB1.1
AFB1.2
AFB2.1
AFB2.2
AFG1.1
AFG1.2
AFG2.1
AFG2.2
Citrinin.1
Citrinin.2
DAS.1
DAS.2
DON.1
DON.2
FB1.1
FB1.2
FB2.1
FB2.2
FX.1
FX.2
HT2.1
HT2.2
NEOS.1
NEOS.2
NIV.1
NIV.2
OTA.1
OTA.2
Sterig.1
Sterig.2
ZONE.1
ZONE.2
FB3.1
FB3.3
15-AC.4
15-AC.5
T2.1
T2.2
CIT-neg.1
CIT-neg.2
DON-neg.1

All Sample Types

Index	Sample Name	Sample Type	Component Name	Component Group Name	Outlier Reasons	Actual Concentration	Area	Height	Retention Time	Width at 50%	Signal / Noise	Used	Calculated Concentration	Accuracy	Ion Ratio	Expected Ion Ratio
23	Neat Standard_1	Standard	HT2.1	HT2	Accurac...	0.0063	33207.300	8.178e3	8.34	0.06	171.5	☐	0.040	636.08	0.7868	0.0088
75	Neat Standard_2	Standard	HT2.1	HT2	Accurac...	0.0125	58437.603	1.469e4	8.32	0.06	262.8	☐	0.044	351.89	0.8448	0.0088
127	Neat Standard_3	Standard	HT2.1	HT2	Accurac...	0.0250	117203.557	2.739e4	8.33	0.07	510.2	☐	0.053	212.40	0.7718	0.0088
179	Neat Standard_4	Standard	HT2.1	HT2	Accurac...	0.0625	299540.609	7.151e4	8.34	0.06	1027.7	☑	0.081	130.21	0.7942	0.0088
231	Neat Standard_5	Standard	HT2.1	HT2	Ion Ratio	0.1250	618120.909	1.520e5	8.34	0.06	1706.7	☑	0.131	104.63	0.7946	0.0088
283	Neat Standard_6	Standard	HT2.1	HT2		0.2500	1186510.374	2.901e5	8.33	0.06	1551.0	☑	0.219	87.58	0.0085	0.0088
335	Neat Standard_7	Standard	HT2.1	HT2		0.6250	3333594.8...	8.076e5	8.36	0.06	2367.1	☑	0.552	88.31	0.0094	0.0088
387	Neat Standard_8	Standard	HT2.1	HT2		1.2500	6786326.6...	1.681e6	8.36	0.06	1841.5	☑	1.087	87.00	0.0083	0.0088
439	Neat Standard_9	Standard	HT2.1	HT2	Ion Ratio	2.5000	14960356...	3.866e6	8.37	0.06	2764.9	☑	2.355	94.21	N/A	0.0088
491	Neat Standard_10	Standard	HT2.1	HT2	Ion Ratio	6.2500	44067885...	1.073e7	8.34	0.06	2241.6	☑	6.870	109.92	0.8005	0.0088
543	Neat Standard_11	Standard	HT2.1	HT2		12.5000	78864637...	1.850e7	8.34	0.07	2443.6	☑	12.267	98.13	0.0083	0.0088
595	Neat Standard_12	Standard	HT2.1	HT2		25.0000	13520915...	2.707e7	8.34	0.08	1945.3	☐	21.006	84.02	0.0103	0.0088

Regression

Calibration for HT2.1: y = 6.44746e6 x + -2.25162e5 (r = 0.99737) (weighting: 1 / x)

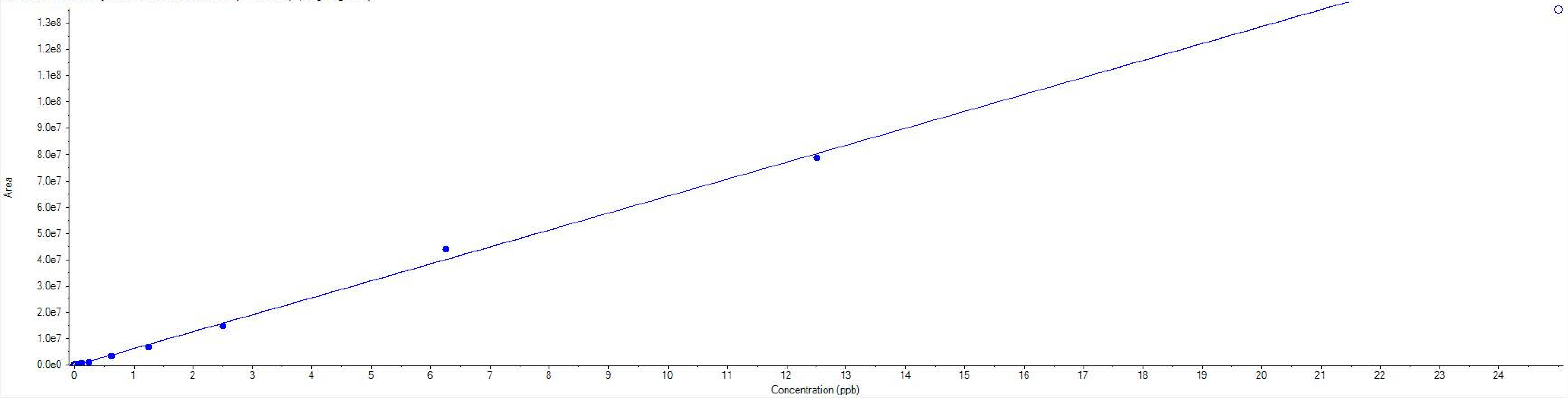

MultiQuant - [[MQ4] Results Table (Untitled)]

File Edit Process Window Help

Mycotoxins\2017

Components & Groups IS

All Components

3 Acetyl DON Group
AFB1 Group
AFB2 Group
AFG1 Group
AFG2 Group
Citrinin Group
DAS Group
DON Group
FB1 Group
FB2 Group
FX Group
HT2 Group
NEOS Group
NIV Group
OTA Group
Sterig Group
ZONE Group
FB3 Group
15-AC Group
T2 Group
CIT-neg Group
DON-neg Group
NIV-neg Group
ZON-neg Group
3AC-neg Group

3 Acetyl DON.1
3 Acetyl DON.2
AFB1.1
AFB1.2
AFB2.1
AFB2.2
AFG1.1
AFG1.2
AFG2.1
AFG2.2
Citrinin.1
Citrinin.2
DAS.1
DAS.2
DON.1
DON.2
FB1.1
FB1.2
FB2.1
FB2.2
FX.1
FX.2
HT2.1
HT2.2
NEOS.1
NEOS.2
NIV.1
NIV.2
OTA.1
OTA.2
Sterig.1
Sterig.2
ZONE.1
ZONE.2
FB3.1
FB3.3
15-AC.4
15-AC.5
T2.1
T2.2
CIT-neg.1
CIT-neg.2
DON-neg.1

All Sample Types

Index	Sample Name	Sample Type	Component Name	Component Group Name	Outlier Reasons	Actual Concentration	Area	Height	Retention Time	Width at 50%	Signal / Noise	Used	Calculated Concentration	Accuracy	Ion Ratio	Expected Ion Ratio
25	Neat Standard_1	Standard	NEOS.1	NEOS	Accuracy	0.0063	329922.293	1.143e5	4.54	0.04	720.7	☐	< 0	N/A	0.2573	0.2800
77	Neat Standard_2	Standard	NEOS.1	NEOS	Accuracy	0.0125	553337.891	1.901e5	4.52	0.04	1014.9	☐	< 0	N/A	0.2537	0.2800
129	Neat Standard_3	Standard	NEOS.1	NEOS	Accuracy	0.0250	1031928.6...	3.558e5	4.52	0.04	1219.2	☐	< 0	N/A	0.2722	0.2800
181	Neat Standard_4	Standard	NEOS.1	NEOS	Accuracy	0.0625	2669915.9...	9.209e5	4.53	0.04	1415.3	☑	0.044	70.22	0.2717	0.2800
233	Neat Standard_5	Standard	NEOS.1	NEOS		0.1250	5093738.5...	1.731e6	4.52	0.05	1919.2	☑	0.131	104.71	0.2634	0.2800
285	Neat Standard_6	Standard	NEOS.1	NEOS		0.2500	9266031.2...	3.180e6	4.52	0.04	1630.9	☑	0.281	112.25	0.2740	0.2800
337	Neat Standard_7	Standard	NEOS.1	NEOS		0.6250	21338747....	7.183e6	4.53	0.05	1907.6	☑	0.714	114.23	0.2705	0.2800
389	Neat Standard_8	Standard	NEOS.1	NEOS		1.2500	38272068....	1.241e7	4.53	0.05	1250.3	☑	1.322	105.73	0.2803	0.2800
441	Neat Standard_9	Standard	NEOS.1	NEOS		2.5000	66130157....	2.065e7	4.57	0.05	1990.1	☑	2.322	92.86	0.2891	0.2800
493	Neat Standard_10	Standard	NEOS.1	NEOS	Accuracy	6.2500	12268657...	3.600e7	4.54	0.05	1467.0	☐	4.351	69.62	0.3092	0.2800
545	Neat Standard_11	Standard	NEOS.1	NEOS	Accuracy	12.5000	17595491...	4.962e7	4.53	0.05	1008.5	☐	6.263	50.11	0.3127	0.2800
597	Neat Standard_12	Standard	NEOS.1	NEOS	Accurac...	25.0000	23238821...	6.028e7	4.54	0.06	726.2	☐	8.289	33.15	0.3370	0.2800

Regression

Calibration for NEOS.1: y = 2.78625e7 x + 1.44706e6 (r = 0.99468) (weighting: 1 / x)

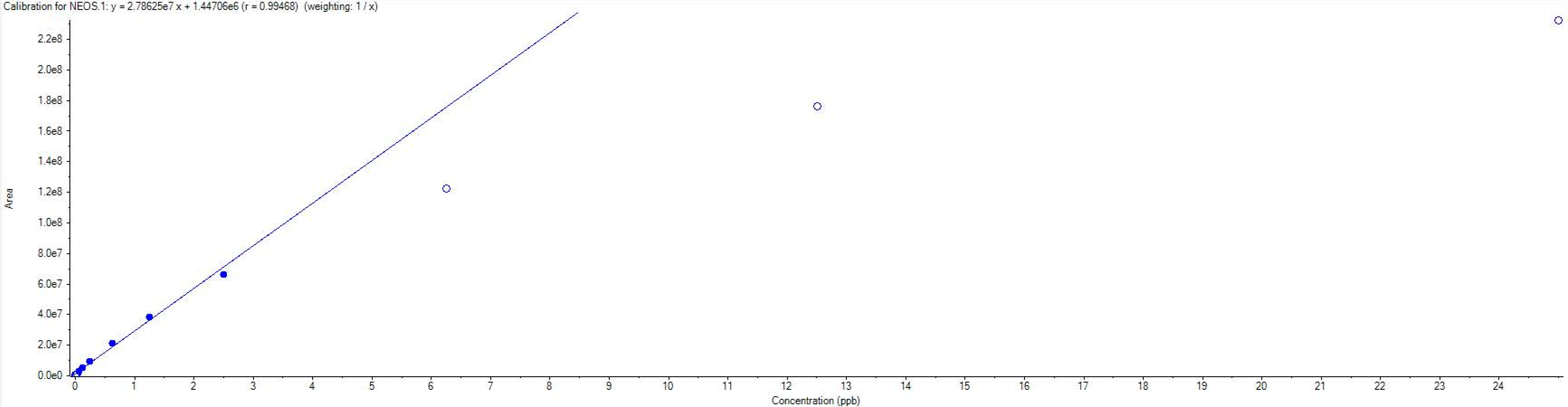

MultiQuant - [[MQ4] Results Table (Untitled)]

File Edit Process Window Help

Mycotoxins\2017

Components & Groups IS

All Components

3 Acetyl DON Group
AFB1 Group
AFB2 Group
AFG1 Group
AFG2 Group
Citrinin Group
DAS Group
DON Group
FB1 Group
FB2 Group
FX Group
HT2 Group
NEOS Group
NIV Group
OTA Group
Sterig Group
ZONE Group
FB3 Group
15-AC Group
T2 Group
CIT-neg Group
DON-neg Group
NIV-neg Group
ZON-neg Group
3AC-neg Group

3 Acetyl DON.1
3 Acetyl DON.2
AFB1.1
AFB1.2
AFB2.1
AFB2.2
AFG1.1
AFG1.2
AFG2.1
AFG2.2
Citrinin.1
Citrinin.2
DAS.1
DAS.2
DON.1
DON.2
FB1.1
FB1.2
FB2.1
FB2.2
FX.1
FX.2
HT2.1
HT2.2
NEOS.1
NEOS.2
NIV.1
NIV.2
OTA.1
OTA.2
Sterig.1
Sterig.2
ZONE.1
ZONE.2
FB3.1
FB3.3
15-AC.4
15-AC.5
T2.1
T2.2
CIT-neg.1
CIT-neg.2
DON-neg.1

All Sample Types

Index	Sample Name	Sample Type	Component Name	Component Group Name	Outlier Reasons	Actual Concentration	Area	Height	Retention Time	Width at 50%	Signal / Noise	Used	Calculated Concentration	Accuracy	Ion Ratio	Expected Ion Ratio
21	Neat Standard_1	Standard	FX.1	FX	Accurac...	0.0125	19169.561	6.515e3	4.50	0.05	32.6	☐	< 0	N/A	0.8368	0.4085
73	Neat Standard_2	Standard	FX.1	FX	Accurac...	0.0250	30115.759	1.043e4	4.48	0.04	57.0	☐	< 0	N/A	0.8600	0.4085
125	Neat Standard_3	Standard	FX.1	FX	Accuracy	0.0500	55408.898	1.843e4	4.48	0.04	111.8	☑	0.021	41.08	0.3945	0.4085
177	Neat Standard_4	Standard	FX.1	FX		0.1250	143401.570	5.252e4	4.48	0.04	215.6	☑	0.126	100.93	0.3855	0.4085
229	Neat Standard_5	Standard	FX.1	FX		0.2500	272159.437	9.553e4	4.48	0.04	343.4	☑	0.281	112.29	0.4256	0.4085
281	Neat Standard_6	Standard	FX.1	FX		0.5000	531398.516	1.922e5	4.47	0.04	576.7	☑	0.592	118.39	0.4355	0.4085
333	Neat Standard_7	Standard	FX.1	FX		1.2500	1247935.402	4.559e5	4.49	0.04	673.7	☑	1.452	116.17	0.4140	0.4085
385	Neat Standard_8	Standard	FX.1	FX		2.5000	2397950.4...	8.664e5	4.49	0.04	1316.2	☑	2.833	113.30	0.4107	0.4085
437	Neat Standard_9	Standard	FX.1	FX		5.0000	4402638.7...	1.531e6	4.52	0.04	1407.9	☑	5.239	104.78	0.4008	0.4085
489	Neat Standard_10	Standard	FX.1	FX		12.5000	9728000.9...	3.442e6	4.49	0.04	1640.6	☑	11.632	93.05	0.4085	0.4085
541	Neat Standard_11	Standard	FX.1	FX	Accuracy	25.0000	14726097....	5.109e6	4.49	0.04	1857.5	☐	17.632	70.53	0.4181	0.4085
593	Neat Standard_12	Standard	FX.1	FX	Accuracy	50.0000	21734163....	7.201e6	4.49	0.05	1991.6	☐	26.044	52.09	0.4265	0.4085

Regression

Calibration for FX.1: y = 8.33033e5 x + 3.82993e4 (r = 0.99545) (weighting: 1 / x)

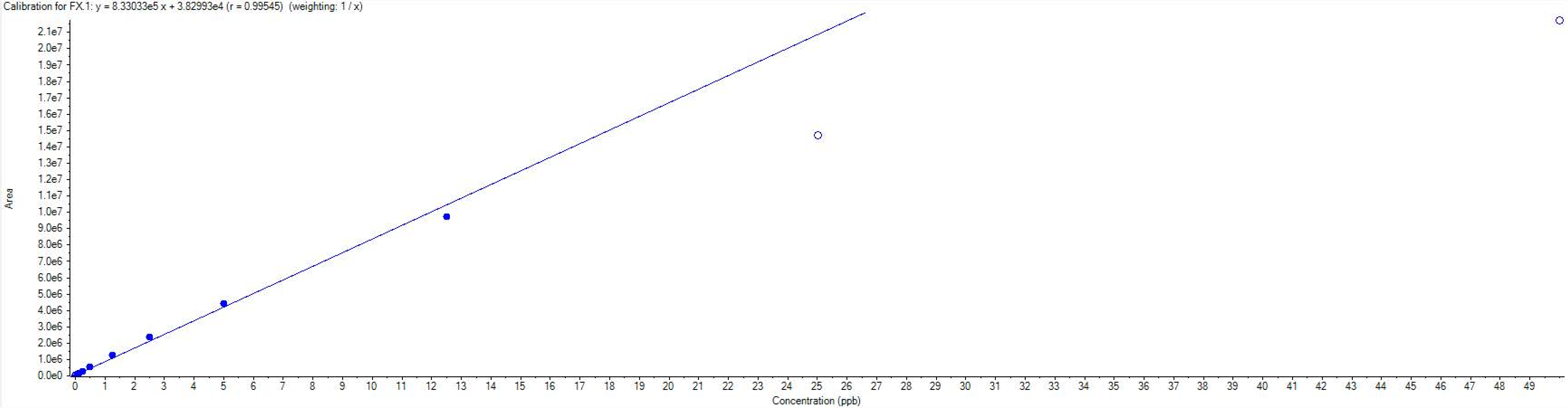

MultiQuant - [[MQ4] Results Table (Untitled)]

File Edit Process Window Help

Mycotoxins\2017

Components & Groups IS

All Components

3 Acetyl DON Group
AFB1 Group
AFB2 Group
AFG1 Group
AFG2 Group
Citrinin Group
DAS Group
DON Group
FB1 Group
FB2 Group
FX Group
HT2 Group
NEOS Group
NIV Group
OTA Group
Sterig Group
ZONE Group
FB3 Group
15-AC Group
T2 Group
CIT-neg Group
DON-neg Group
NIV-neg Group
ZON-neg Group
3AC-neg Group

3 Acetyl DON.1
3 Acetyl DON.2
AFB1.1
AFB1.2
AFB2.1
AFB2.2
AFG1.1
AFG1.2
AFG2.1
AFG2.2
Citrinin.1
Citrinin.2
DAS.1
DAS.2
DON.1
DON.2
FB1.1
FB1.2
FB2.1
FB2.2
FX.1
FX.2
HT2.1
HT2.2
NEOS.1
NEOS.2
NIV.1
NIV.2
OTA.1
OTA.2
Sterig.1
Sterig.2
ZONE.1
ZONE.2
FB3.1
FB3.3
15-AC.4
15-AC.5
T2.1
T2.2
CIT-neg.1
CIT-neg.2
DON-neg.1

All Sample Types

Index	Sample Name	Sample Type	Component Name	Component Group Name	Outlier Reasons	Actual Concentration	Area	Height	Retention Time	Width at 50%	Signal / Noise	Used	Calculated Concentration	Accuracy	Ion Ratio	Expected Ion Ratio
35	Neat Standard_1	Standard	FB3.1	FB3	Accurac...	0.0031	10659.295	2.825e3	9.72	0.05	40.8	☐	0.176	5685.01	0.5012	0.3908
87	Neat Standard_2	Standard	FB3.1	FB3	Accurac...	0.0063	19894.391	4.656e3	9.70	0.05	26.9	☐	0.178	2818.87	1.1964	0.3908
139	Neat Standard_3	Standard	FB3.1	FB3	Accurac...	0.0125	32090.662	8.473e3	9.71	0.06	39.1	☐	0.179	1435.01	1.3287	0.3908
191	Neat Standard_4	Standard	FB3.1	FB3	Accuracy	0.0312	89523.337	2.258e4	9.71	0.05	209.7	☐	0.188	601.90	0.3978	0.3908
243	Neat Standard_5	Standard	FB3.1	FB3	Accurac...	0.0625	207595.902	5.144e4	9.71	0.05	440.4	☑	0.205	328.15	1.0299	0.3908
295	Neat Standard_6	Standard	FB3.1	FB3	Accurac...	0.1250	434593.327	1.173e5	9.70	0.05	692.1	☑	0.238	190.69	1.0800	0.3908
347	Neat Standard_7	Standard	FB3.1	FB3		0.3120	1294581.1...	3.567e5	9.72	0.05	1067.3	☑	0.364	116.79	0.3969	0.3908
399	Neat Standard_8	Standard	FB3.1	FB3		0.6250	2903238.7...	7.310e5	9.72	0.05	1470.8	☑	0.600	96.02	0.3748	0.3908
451	Neat Standard_9	Standard	FB3.1	FB3		1.2500	6070950.1...	1.674e6	9.72	0.05	1352.4	☑	1.064	85.15	0.4008	0.3908
503	Neat Standard_10	Standard	FB3.1	FB3		3.1250	18342246....	5.385e6	9.71	0.05	2073.0	☑	2.863	91.61	0.3891	0.3908
555	Neat Standard_11	Standard	FB3.1	FB3		6.2500	42578390....	1.233e7	9.71	0.05	2077.1	☑	6.414	102.63	0.3970	0.3908
607	Neat Standard_12	Standard	FB3.1	FB3		12.5000	93157498....	2.700e7	9.72	0.05	2341.1	☐	13.827	110.61	0.4075	0.3908

Regression

Calibration for FB3.1: y = 6.82370e6 x + -1.19192e6 (r = 0.99733) (weighting: None)

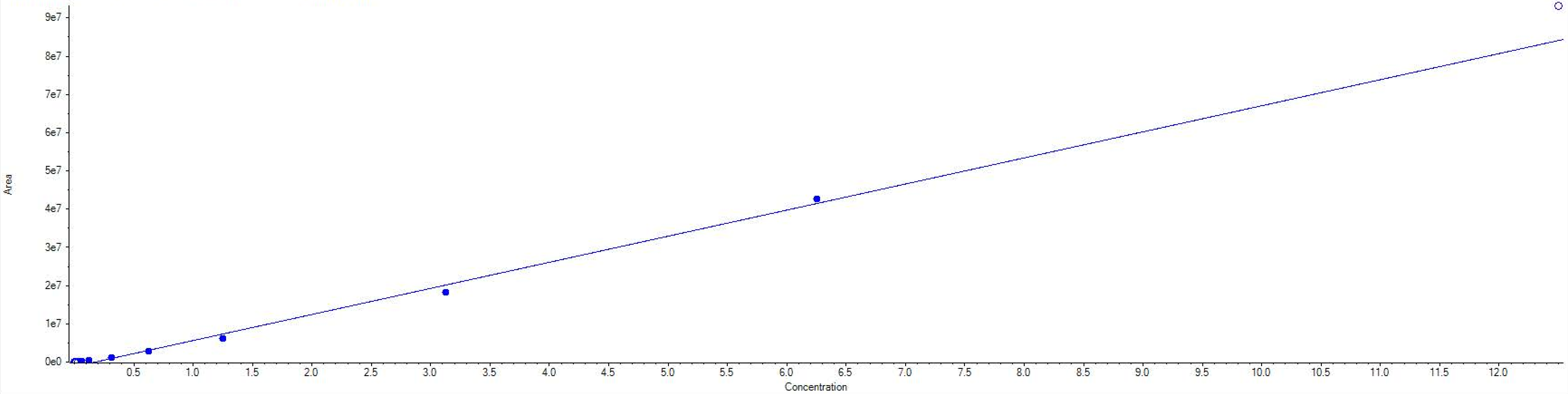

MultiQuant - [[MQ4] Results Table (Untitled)]

File Edit Process Window Help

Mycotoxins\2017

Components & Groups IS

All Components

3 Acetyl DON Group
AFB1 Group
AFB2 Group
AFG1 Group
AFG2 Group
Citrinin Group
DAS Group
DON Group
FB1 Group
FB2 Group
FX Group
HT2 Group
NEOS Group
NIV Group
OTA Group
Sterig Group
ZONE Group
FB3 Group
15-AC Group
T2 Group
CIT-neg Group
DON-neg Group
NIV-neg Group
ZON-neg Group
3AC-neg Group

3 Acetyl DON.1
3 Acetyl DON.2
AFB1.1
AFB1.2
AFB2.1
AFB2.2
AFG1.1
AFG1.2
AFG2.1
AFG2.2
Citrinin.1
Citrinin.2
DAS.1
DAS.2
DON.1
DON.2
FB1.1
FB1.2
FB2.1
FB2.2
FX.1
FX.2
HT2.1
HT2.2
NEOS.1
NEOS.2
NIV.1
NIV.2
OTA.1
OTA.2
Sterig.1
Sterig.2
ZONE.1
ZONE.2
FB3.1
FB3.3
15-AC.4
15-AC.5
T2.1
T2.2
CIT-neg.1
CIT-neg.2
DON-neg.1

All Sample Types

Index	Sample Name	Sample Type	Component Name	Component Group Name	Outlier Reasons	Actual Concentration	Area	Height	Retention Time	Width at 50%	Signal / Noise	Used	Calculated Concentration	Accuracy	Ion Ratio	Expected Ion Ratio
19	Neat Standard_1	Standard	FB2.1	FB2	Accuracy	0.0063	35176.366	8.155e3	10.20	0.06	116.1	☐	0.093	1470.34	0.2574	0.2157
71	Neat Standard_2	Standard	FB2.1	FB2	Accuracy	0.0125	60972.217	1.376e4	10.18	0.06	147.7	☐	0.095	762.18	0.2158	0.2157
123	Neat Standard_3	Standard	FB2.1	FB2	Accuracy	0.0250	92047.120	2.371e4	10.19	0.05	187.2	☐	0.098	393.81	0.2171	0.2157
175	Neat Standard_4	Standard	FB2.1	FB2	Accuracy	0.0625	248480.213	5.797e4	10.19	0.06	255.9	☑	0.114	183.15	0.2155	0.2157
227	Neat Standard_5	Standard	FB2.1	FB2		0.1250	488395.541	1.168e5	10.19	0.06	341.4	☑	0.139	111.22	0.2164	0.2157
279	Neat Standard_6	Standard	FB2.1	FB2		0.2500	1085202.683	2.815e5	10.18	0.06	429.0	☑	0.200	80.05	0.2134	0.2157
331	Neat Standard_7	Standard	FB2.1	FB2	Accuracy	0.6250	3310338.8...	9.338e5	10.20	0.05	385.0	☑	0.428	68.46	0.2200	0.2157
383	Neat Standard_8	Standard	FB2.1	FB2	Accuracy	1.2500	7821475.9...	1.961e6	10.20	0.06	456.1	☑	0.890	71.17	0.2151	0.2157
435	Neat Standard_9	Standard	FB2.1	FB2		2.5000	18681999...	5.106e6	10.20	0.05	500.1	☑	2.001	80.05	0.2121	0.2157
487	Neat Standard_10	Standard	FB2.1	FB2		6.2500	61605756...	1.707e7	10.19	0.05	567.2	☑	6.395	102.32	0.2207	0.2157
539	Neat Standard_11	Standard	FB2.1	FB2		12.5000	13663246...	3.636e7	10.19	0.06	609.8	☐	14.075	112.60	0.2405	0.2157
591	Neat Standard_12	Standard	FB2.1	FB2	Ion Ratio	25.0000	25210319...	5.912e7	10.20	0.06	466.4	☑	25.895	103.58	0.2660	0.2157

Regression

Calibration for FB2.1: y = 9.76923e6 x + -8.69762e5 (r = 0.99481) (weighting: 1 / x)

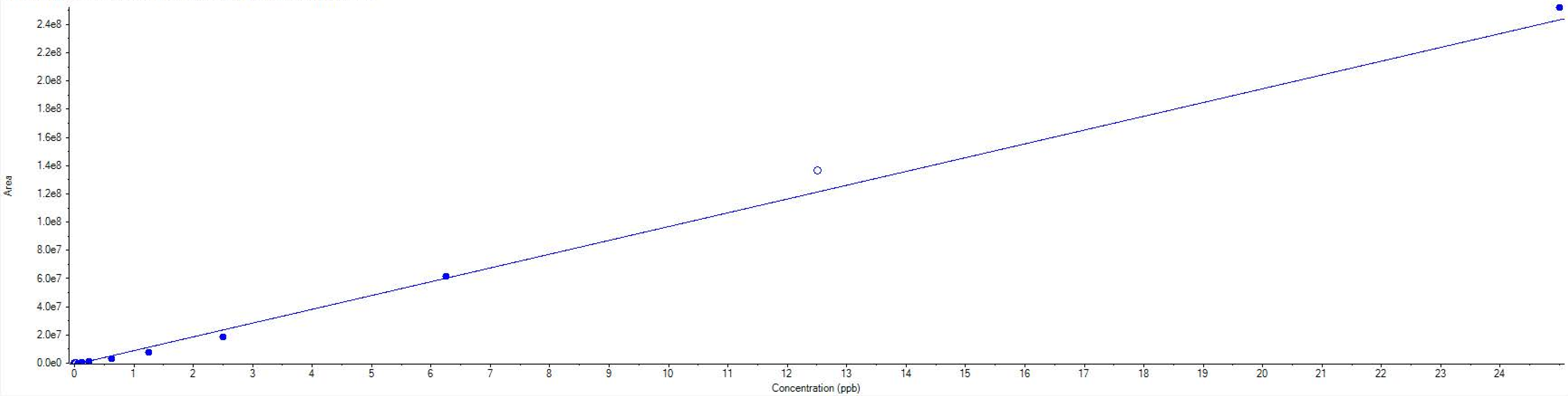

MultiQuant - [[MQ4] Results Table (Untitled)]

File Edit Process Window Help

Mycotoxins\2017

Components & Groups IS

All Components

3 Acetyl DON Group
AFB1 Group
AFB2 Group
AFG1 Group
AFG2 Group
Citrinin Group
DAS Group
DON Group
FB1 Group
FB2 Group
FX Group
HT2 Group
NEOS Group
NIV Group
OTA Group
Sterig Group
ZONE Group
FB3 Group
15-AC Group
T2 Group
CIT-neg Group
DON-neg Group
NIV-neg Group
ZON-neg Group
3AC-neg Group

3 Acetyl DON.1
3 Acetyl DON.2
AFB1.1
AFB1.2
AFB2.1
AFB2.2
AFG1.1
AFG1.2
AFG2.1
AFG2.2
Citrinin.1
Citrinin.2
DAS.1
DAS.2
DON.1
DON.2
FB1.1
FB1.2
FB2.1
FB2.2
FX.1
FX.2
HT2.1
HT2.2
NEOS.1
NEOS.2
NIV.1
NIV.2
OTA.1
OTA.2
Sterig.1
Sterig.2
ZONE.1
ZONE.2
FB3.1
FB3.3
15-AC.4
15-AC.5
T2.1
T2.2
CIT-neg.1
CIT-neg.2
DON-neg.1
[illegible]

All Sample Types

Index	Sample Name	Sample Type	Component Name	Component Group Name	Outlier Reasons	Actual Concentration	Area	Height	Retention Time	Width at 50%	Signal / Noise	Used	Calculated Concentration	Accuracy	Ion Ratio	Expected Ion Ratio
17	Neat Standard_1	Standard	FB1.1	FB1	Accurac...	0.0125	24508.957	8.155e3	9.00	0.05	75.6	☐	0.087	697.85	0.1216	0.0894
69	Neat Standard_2	Standard	FB1.1	FB1	Accuracy	0.0250	46802.362	1.316e4	8.99	0.06	97.5	☐	0.093	371.10	0.0896	0.0894
121	Neat Standard_3	Standard	FB1.1	FB1	Accurac...	0.0500	81310.662	2.325e4	8.99	0.06	129.2	☑	0.101	202.71	0.1229	0.0894
173	Neat Standard_4	Standard	FB1.1	FB1	Ion Ratio	0.1250	228860.607	6.352e4	9.00	0.05	180.8	☑	0.138	110.44	0.1092	0.0894
225	Neat Standard_5	Standard	FB1.1	FB1	Accuracy	0.2500	472867.329	1.315e5	9.00	0.05	204.1	☑	0.199	79.49	0.0946	0.0894
277	Neat Standard_6	Standard	FB1.1	FB1	Accurac...	0.5000	937773.478	2.720e5	9.00	0.05	232.8	☑	0.314	62.87	0.1234	0.0894
329	Neat Standard_7	Standard	FB1.1	FB1	Accuracy	1.2500	3470350.0...	8.932e5	9.01	0.05	416.2	☑	0.944	75.54	0.0898	0.0894
381	Neat Standard_8	Standard	FB1.1	FB1	Accuracy	2.5000	7546158.1...	1.850e6	9.01	0.05	530.0	☑	1.958	78.31	0.0896	0.0894
433	Neat Standard_9	Standard	FB1.1	FB1		5.0000	16146933....	4.254e6	9.02	0.05	625.9	☑	4.097	81.94	0.0887	0.0894
485	Neat Standard_10	Standard	FB1.1	FB1		12.5000	50719061....	1.329e7	9.00	0.05	564.4	☑	12.695	101.56	0.0923	0.0894
537	Neat Standard_11	Standard	FB1.1	FB1		25.0000	10759444...	2.777e7	9.00	0.05	690.6	☑	26.839	107.36	0.0971	0.0894
589	Neat Standard_12	Standard	FB1.1	FB1	Ion Ratio	50.0000	20028052...	4.714e7	9.00	0.06	551.7	☑	49.890	99.78	0.1087	0.0894

Regression

Calibration for FB1.1: y = 4.02100e6 x + -3.26248e5 (r = 0.99669) (weighting: 1 / x)

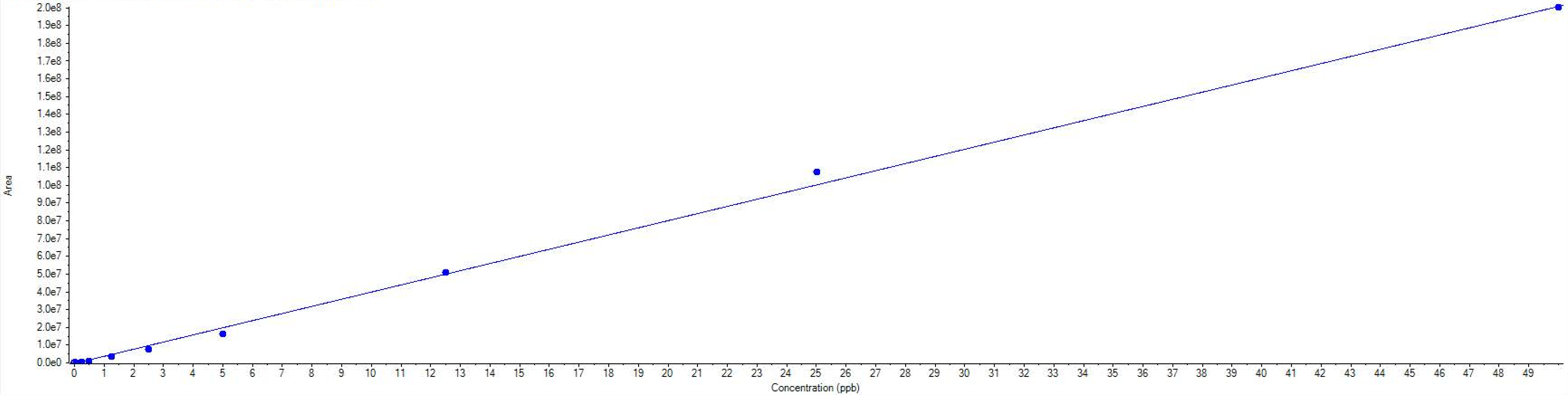

MultiQuant - [[MQ4] Results Table (Untitled)]

File Edit Process Window Help

Mycotoxins\2017

Components & Groups IS

All Components

3 Acetyl DON Group
AFB1 Group
AFB2 Group
AFG1 Group
AFG2 Group
Citrinin Group
DAS Group
DON Group
FB1 Group
FB2 Group
FX Group
HT2 Group
NEOS Group
NIV Group
OTA Group
Sterig Group
ZONE Group
FB3 Group
15-AC Group
T2 Group
CIT-neg Group
DON-neg Group
NIV-neg Group
ZON-neg Group
3AC-neg Group

3 Acetyl DON.1
3 Acetyl DON.2
AFB1.1
AFB1.2
AFB2.1
AFB2.2
AFG1.1
AFG1.2
AFG2.1
AFG2.2
Citrinin.1
Citrinin.2
DAS.1
DAS.2
DON.1
DON.2
FB1.1
FB1.2
FB2.1
FB2.2
FX.1
FX.2
HT2.1
HT2.2
NEOS.1
NEOS.2
NIV.1
NIV.2
OTA.1
OTA.2
Sterig.1
Sterig.2
ZONE.1
ZONE.2
FB3.1
FB3.3
15-AC.4
15-AC.5
T2.1
T2.2
CIT-neg.1
CIT-neg.2
DON-neg.1

All Sample Types

Index	Sample Name	Sample Type	Component Name	Component Group Name	Outlier Reasons	Actual Concentration	Area	Height	Retention Time	Width at 50%	Signal / Noise	Used	Calculated Concentration	Accuracy	Ion Ratio	Expected Ion Ratio
15	Neat Standard_1	Standard	DON.1	DON		0.0125	96975.314	3.548e4	4.30	0.04	134.9	☑	0.011	87.88	0.1994	0.2176
67	Neat Standard_2	Standard	DON.1	DON		0.0250	157343.726	5.748e4	4.28	0.04	162.2	☑	0.022	87.63	0.2413	0.2176
119	Neat Standard_3	Standard	DON.1	DON		0.0500	296283.812	1.076e5	4.28	0.04	249.8	☑	0.047	94.10	0.2385	0.2176
171	Neat Standard_4	Standard	DON.1	DON		0.1250	788673.524	2.943e5	4.29	0.04	598.1	☑	0.136	108.91	0.2217	0.2176
223	Neat Standard_5	Standard	DON.1	DON		0.2500	1529303.2...	5.532e5	4.28	0.04	891.4	☑	0.270	108.06	0.2090	0.2176
275	Neat Standard_6	Standard	DON.1	DON		0.5000	2886734.9...	1.077e6	4.28	0.04	961.3	☑	0.516	103.15	0.2188	0.2176
327	Neat Standard_7	Standard	DON.1	DON		1.2500	7346989.735	2.670e6	4.30	0.04	1465.3	☑	1.323	105.82	0.2132	0.2176
379	Neat Standard_8	Standard	DON.1	DON		2.5000	14344971...	5.113e6	4.29	0.04	1175.2	☑	2.589	103.56	0.2200	0.2176
431	Neat Standard_9	Standard	DON.1	DON		5.0000	28812078...	9.931e6	4.33	0.04	1818.3	☑	5.207	104.13	0.2197	0.2176
483	Neat Standard_10	Standard	DON.1	DON		12.5000	66864106...	2.324e7	4.30	0.04	1262.5	☑	12.092	96.74	0.2188	0.2176
535	Neat Standard_11	Standard	DON.1	DON	Accuracy	25.0000	10810046...	3.541e7	4.29	0.05	1104.9	☐	19.553	78.21	0.2384	0.2176
587	Neat Standard_12	Standard	DON.1	DON	Accuracy	50.0000	16235373...	4.952e7	4.30	0.05	947.2	☐	29.370	58.74	0.2540	0.2176

Regression

Calibration for DON.1: y = 5.52666e6 x + 3.62625e4 (r = 0.99923) (weighting: 1 / x)

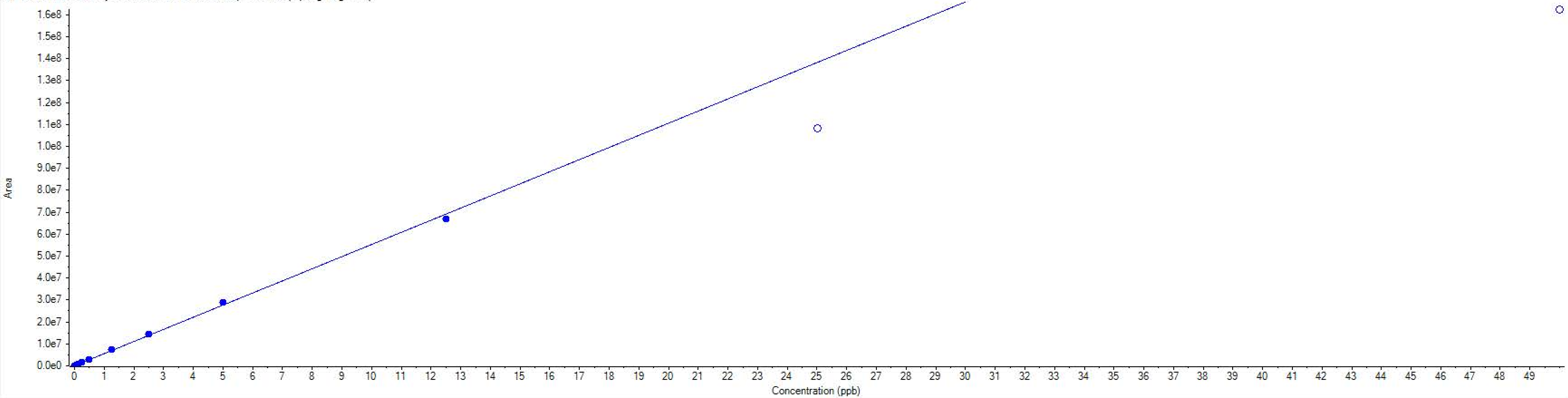

MultiQuant - [[MQ4] Results Table (Untitled)]

File Edit Process Window Help

Mycotoxins\2017

Components & Groups IS

All Components

3 Acetyl DON Group
AFB1 Group
AFB2 Group
AFG1 Group
AFG2 Group
Citrinin Group
DAS Group
DON Group
FB1 Group
FB2 Group
FX Group
HT2 Group
NEOS Group
NIV Group
OTA Group
Sterig Group
ZONE Group
FB3 Group
15-AC Group
T2 Group
CIT-neg Group
DON-neg Group
NIV-neg Group
ZON-neg Group
3AC-neg Group

3 Acetyl DON.1
3 Acetyl DON.2
AFB1.1
AFB1.2
AFB2.1
AFB2.2
AFG1.1
AFG1.2
AFG2.1
AFG2.2
Citrinin.1
Citrinin.2
DAS.1
DAS.2
DON.1
DON.2
FB1.1
FB1.2
FB2.1
FB2.2
FX.1
FX.2
HT2.1
HT2.2
NEOS.1
NEOS.2
NIV.1
NIV.2
OTA.1
OTA.2
Sterig.1
Sterig.2
ZONE.1
ZONE.2
FB3.1
FB3.3
15-AC.4
15-AC.5
T2.1
T2.2
CIT-neg.1
CIT-neg.2
DON-neg.1

All Sample Types

Index	Sample Name	Sample Type	Component Name	Component Group Name	Outlier Reasons	Actual Concentration	Area	Height	Retention Time	Width at 50%	Signal / Noise	Used	Calculated Concentration	Accuracy	Ion Ratio	Expected Ion Ratio
13	Neat Standard_1	Standard	DAS.1	DAS	Accuracy	0.0063	22012.729	5.305e3	6.19	0.06	55.1	☐	< 0	N/A	0.3994	0.3560
65	Neat Standard_2	Standard	DAS.1	DAS	Accuracy	0.0125	36628.775	8.010e3	6.18	0.07	87.5	☑	0.005	41.05	0.2953	0.3560
117	Neat Standard_3	Standard	DAS.1	DAS		0.0250	72542.323	1.596e4	6.18	0.07	135.9	☑	0.022	88.95	0.3091	0.3560
169	Neat Standard_4	Standard	DAS.1	DAS		0.0625	182655.841	3.929e4	6.19	0.07	325.0	☑	0.075	119.49	0.3369	0.3560
221	Neat Standard_5	Standard	DAS.1	DAS		0.1250	334191.993	7.215e4	6.19	0.07	448.0	☑	0.147	117.49	0.3853	0.3560
273	Neat Standard_6	Standard	DAS.1	DAS	Accuracy	0.2500	660644.016	1.430e5	6.19	0.07	710.8	☑	0.302	120.94	0.3471	0.3560
325	Neat Standard_7	Standard	DAS.1	DAS		0.6250	1573763.6...	3.268e5	6.21	0.07	956.7	☑	0.737	117.96	0.3582	0.3560
377	Neat Standard_8	Standard	DAS.1	DAS		1.2500	2737436.7...	5.580e5	6.21	0.08	875.5	☑	1.292	103.32	0.3543	0.3560
429	Neat Standard_9	Standard	DAS.1	DAS		2.5000	4791589.1...	9.542e5	6.24	0.08	878.3	☑	2.270	90.80	0.3556	0.3560
481	Neat Standard_10	Standard	DAS.1	DAS	Accuracy	6.2500	10239763...	1.922e6	6.20	0.08	874.5	☐	4.865	77.84	0.3534	0.3560
533	Neat Standard_11	Standard	DAS.1	DAS	Accuracy	12.5000	16622079...	3.109e6	6.20	0.08	876.4	☐	7.905	63.24	0.3501	0.3560
585	Neat Standard_12	Standard	DAS.1	DAS	Accuracy	25.0000	25869060...	4.468e6	6.20	0.09	717.9	☐	12.309	49.24	0.3516	0.3560

Regression

Calibration for DAS.1: y = 2.09951e6 x + 25856.53671 (r = 0.99279) (weighting: 1 / x)

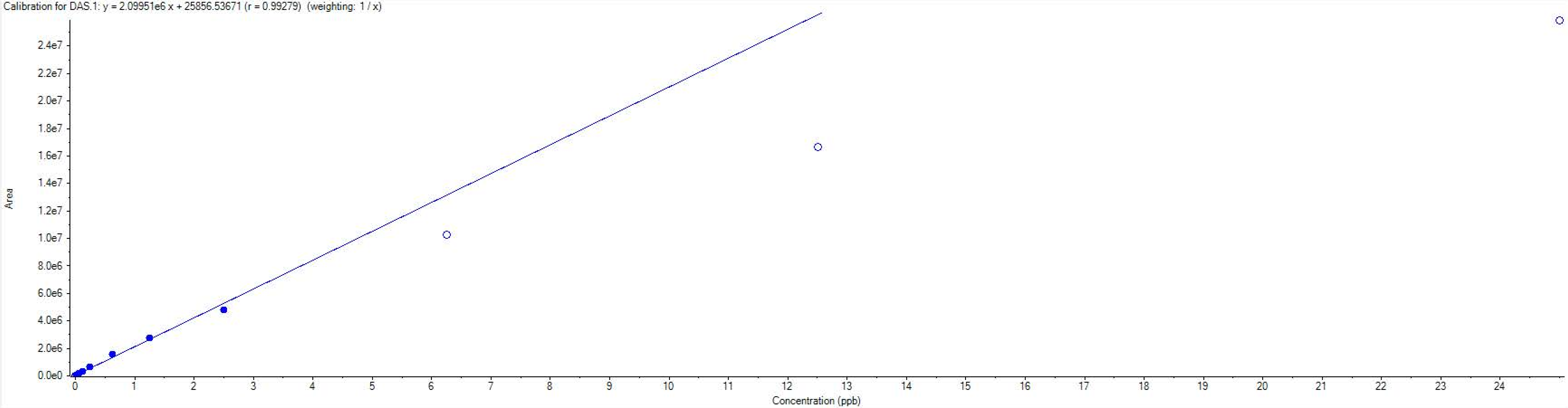

MultiQuant - [[MQ4] Results Table (Untitled)]

File Edit Process Window Help

Mycotoxins\2017

Components & Groups IS

All Sample Types

All Components

3 Acetyl DON Group
AFB1 Group
AFB2 Group
AFG1 Group
AFG2 Group
Citrinin Group
DAS Group
DON Group
FB1 Group
FB2 Group
FX Group
HT2 Group
NEOS Group
NIV Group
OTA Group
Sterig Group
ZONE Group
FB3 Group
15-AC Group
T2 Group
CIT-neg Group
DON-neg Group
NIV-neg Group
ZON-neg Group
3AC-neg Group

3 Acetyl DON.1
3 Acetyl DON.2
AFB1.1
AFB1.2
AFB2.1
AFB2.2
AFG1.1
AFG1.2
AFG2.1
AFG2.2
Citrinin.1
Citrinin.2
DAS.1
DAS.2
DON.1
DON.2
FB1.1
FB1.2
FB2.1
FB2.2
FX.1
FX.2
HT2.1
HT2.2
NEOS.1
NEOS.2
NIV.1
NIV.2
OTA.1
OTA.2
Sterig.1
Sterig.2
ZONE.1
ZONE.2
FB3.1
FB3.3
15-AC.4
15-AC.5
T2.1
T2.2
CIT-neg.1
CIT-neg.2
DON-neg.1
[illegible]

Index	Sample Name	Sample Type	Component Name	Component Group Name	Outlier Reasons	Actual Concentration	Area	Height	Retention Time	Width at 50%	Signal / Noise	Used	Calculated Concentration	Accuracy	Ion Ratio	Expected Ion Ratio
9	Neat Standard_1	Standard	AFG2.1	AFG2	Accuracy	0.0078	35998.686	1.036e4	5.06	0.05	51.4	☐	< 0	N/A	0.5864	0.5653
61	Neat Standard_2	Standard	AFG2.1	AFG2	Accuracy	0.0156	63873.998	1.702e4	5.05	0.06	55.5	☐	< 0	N/A	0.5770	0.5653
113	Neat Standard_3	Standard	AFG2.1	AFG2	Accuracy	0.0312	130101.157	3.457e4	5.06	0.06	111.2	☐	< 0	N/A	0.5002	0.5653
165	Neat Standard_4	Standard	AFG2.1	AFG2	Accurac...	0.0780	271465.398	8.681e4	5.06	0.05	163.5	☑	0.125	160.65	0.6802	0.5653
217	Neat Standard_5	Standard	AFG2.1	AFG2	Accuracy	1.5600	677466.093	1.860e5	5.05	0.05	251.6	☑	1.055	67.60	0.5388	0.5653
269	Neat Standard_6	Standard	AFG2.1	AFG2	Accuracy	3.1200	1282181.5...	3.609e5	5.05	0.05	298.1	☑	2.439	78.17	0.5414	0.5653
321	Neat Standard_7	Standard	AFG2.1	AFG2		7.8000	3385863.2...	9.890e5	5.07	0.05	309.9	☑	7.254	93.00	0.5474	0.5653
373	Neat Standard_8	Standard	AFG2.1	AFG2		15.6000	6898714.8...	1.944e6	5.07	0.05	263.5	☑	15.295	98.04	0.5437	0.5653
425	Neat Standard_9	Standard	AFG2.1	AFG2		31.2000	13667040...	3.786e6	5.12	0.05	265.3	☑	30.787	98.68	0.5403	0.5653
477	Neat Standard_10	Standard	AFG2.1	AFG2		78.0000	35873456...	9.779e6	5.06	0.05	313.6	☑	81.617	104.64	0.5501	0.5653
529	Neat Standard_11	Standard	AFG2.1	AFG2		156.0000	67839024...	1.830e7	5.06	0.05	278.9	☑	154.786	99.22	0.5547	0.5653
581	Neat Standard_12	Standard	AFG2.1	AFG2		312.0000	12373421...	3.190e7	5.06	0.06	293.3	☐	282.728	90.62	0.5573	0.5653

Regression

Calibration for AFG2.1: y = 4.36877e5 x + 2.16723e5 (r = 0.99902) (weighting: 1 / x)

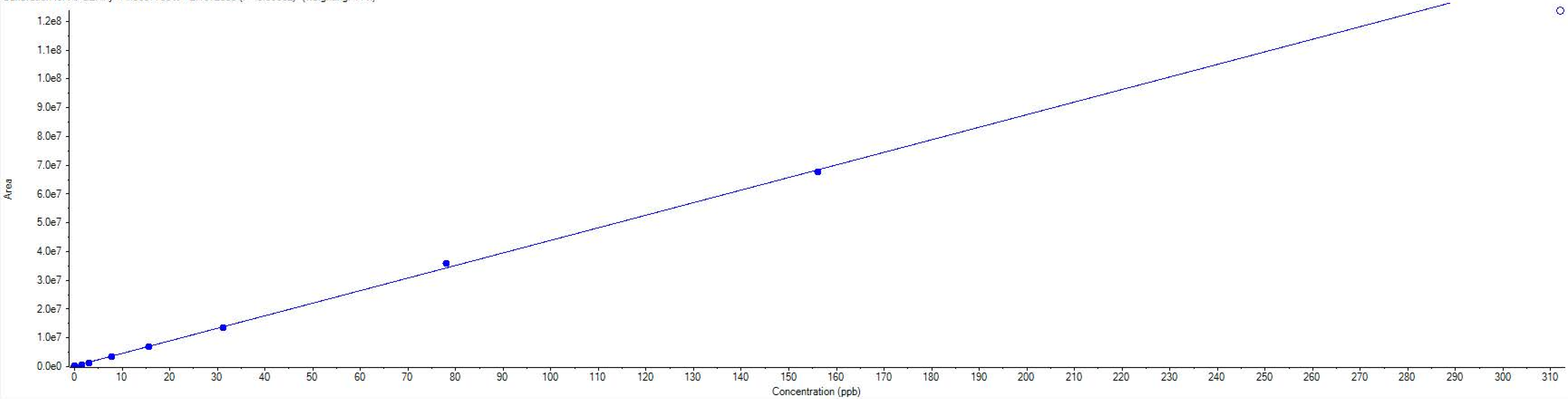

MultiQuant - [[MQ4] Results Table (Untitled)]

File Edit Process Window Help

Mycotoxins\2017

Components & Groups IS

All Components

3 Acetyl DON Group
AFB1 Group
AFB2 Group
AFG1 Group
AFG2 Group
Citrinin Group
DAS Group
DON Group
FB1 Group
FB2 Group
FX Group
HT2 Group
NEOS Group
NIV Group
OTA Group
Sterig Group
ZONE Group
FB3 Group
15-AC Group
T2 Group
CIT-neg Group
DON-neg Group
NIV-neg Group
ZON-neg Group
3AC-neg Group

3 Acetyl DON.1
3 Acetyl DON.2
AFB1.1
AFB1.2
AFB2.1
AFB2.2
AFG1.1
AFG1.2
AFG2.1
AFG2.2
Citrinin.1
Citrinin.2
DAS.1
DAS.2
DON.1
DON.2
FB1.1
FB1.2
FB2.1
FB2.2
FX.1
FX.2
HT2.1
HT2.2
NEOS.1
NEOS.2
NIV.1
NIV.2
OTA.1
OTA.2
Sterig.1
Sterig.2
ZONE.1
ZONE.2
FB3.1
FB3.3
15-AC.4
15-AC.5
T2.1
T2.2
CIT-neg.1
CIT-neg.2
DON-neg.1

All Sample Types

Index	Sample Name	Sample Type	Component Name	Component Group Name	Outlier Reasons	Actual Concentration	Area	Height	Retention Time	Width at 50%	Signal / Noise	Used	Calculated Concentration	Accuracy	Ion Ratio	Expected Ion Ratio
5	Neat Standard_1	Standard	AFB2.1	AFB2	Accuracy	0.0078	38365.661	9.106e3	5.62	0.06	57.8	☐	< 0	N/A	0.8059	0.7403
57	Neat Standard_2	Standard	AFB2.1	AFB2	Accuracy	0.0156	75793.532	1.732e4	5.60	0.06	89.2	☐	< 0	N/A	0.7812	0.7403
109	Neat Standard_3	Standard	AFB2.1	AFB2	Accuracy	0.0312	136778.631	3.036e4	5.61	0.06	113.1	☑	< 0	N/A	0.7727	0.7403
161	Neat Standard_4	Standard	AFB2.1	AFB2	Accuracy	0.0780	377055.088	8.312e4	5.62	0.06	166.7	☑	0.379	486.39	0.7358	0.7403
213	Neat Standard_5	Standard	AFB2.1	AFB2	Accuracy	1.5600	736255.688	1.652e5	5.62	0.06	198.5	☑	1.059	67.88	0.7832	0.7403
265	Neat Standard_6	Standard	AFB2.1	AFB2		3.1200	1616081.7...	3.446e5	5.61	0.06	245.7	☑	2.723	87.29	0.6560	0.7403
317	Neat Standard_7	Standard	AFB2.1	AFB2		7.8000	3843242.1...	8.962e5	5.63	0.06	264.4	☑	6.937	88.94	0.7917	0.7403
369	Neat Standard_8	Standard	AFB2.1	AFB2		15.6000	8352977.5...	1.740e6	5.63	0.06	260.7	☑	15.469	99.16	0.7022	0.7403
421	Neat Standard_9	Standard	AFB2.1	AFB2	Accuracy	31.2000	N/A	N/A	N/A	N/A	N/A	☑	N/A	N/A	N/A	0.7403
473	Neat Standard_10	Standard	AFB2.1	AFB2		78.0000	45875845...	9.489e6	5.62	0.07	284.0	☑	86.458	110.84	0.7129	0.7403
525	Neat Standard_11	Standard	AFB2.1	AFB2		156.0000	86994865...	1.772e7	5.62	0.07	281.5	☑	164.250	105.29	0.7412	0.7403
577	Neat Standard_12	Standard	AFB2.1	AFB2		312.0000	15715719...	3.058e7	5.62	0.07	291.8	☑	296.989	95.19	0.7588	0.7403

Regression

Calibration for AFB2.1: y = 5.28574e5 x + 1.76520e5 (r = 0.99660) (weighting: 1 / x)

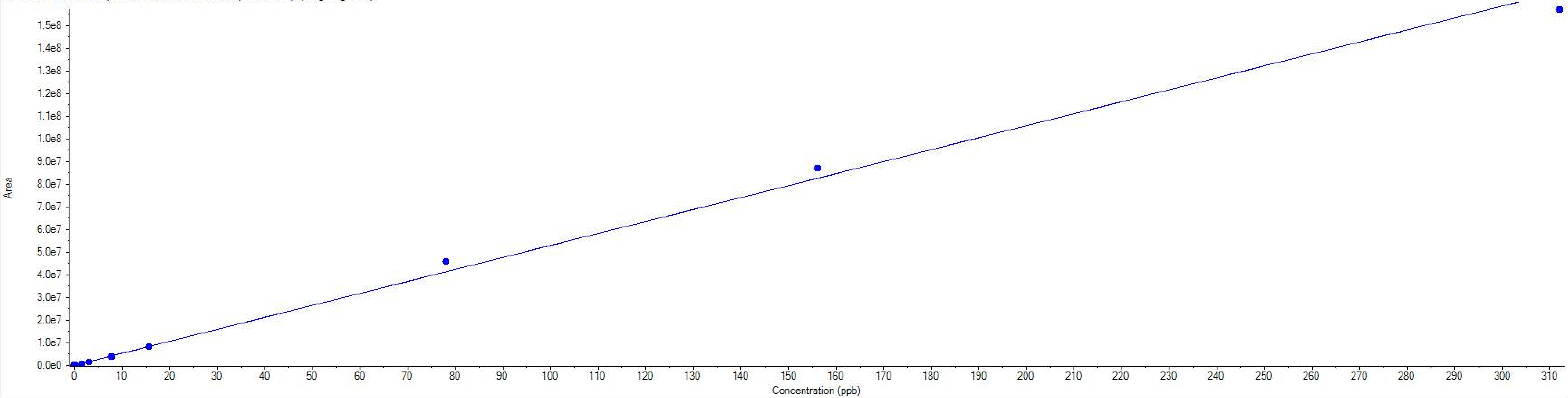

MultiQuant - [[MQ4] Results Table (Untitled)]

File Edit Process Window Help

Mycotoxins\2017

Components & Groups IS

All Components

3 Acetyl DON Group
AFB1 Group
AFB2 Group
AFG1 Group
AFG2 Group
Citrinin Group
DAS Group
DON Group
FB1 Group
FB2 Group
FX Group
HT2 Group
NEOS Group
NIV Group
OTA Group
Sterig Group
ZONE Group
FB3 Group
15-AC Group
T2 Group
CIT-neg Group
DON-neg Group
NIV-neg Group
ZON-neg Group
3AC-neg Group

3 Acetyl DON.1
3 Acetyl DON.2
AFB1.1
AFB1.2
AFB2.1
AFB2.2
AFG1.1
AFG1.2
AFG2.1
AFG2.2
Citrinin.1
Citrinin.2
DAS.1
DAS.2
DON.1
DON.2
FB1.1
FB1.2
FB2.1
FB2.2
FX.1
FX.2
HT2.1
HT2.2
NEOS.1
NEOS.2
NIV.1
NIV.2
OTA.1
OTA.2
Sterig.1
Sterig.2
ZONE.1
ZONE.2
FB3.1
FB3.3
15-AC.4
15-AC.5
T2.1
T2.2
CIT-neg.1
CIT-neg.2
DON-neg.1

All Sample Types

Index	Sample Name	Sample Type	Component Name	Component Group Name	Outlier Reasons	Actual Concentration	Area	Height	Retention Time	Width at 50%	Signal / Noise	Used	Calculated Concentration	Accuracy	Ion Ratio	Expected Ion Ratio
7	Neat Standard_1	Standard	AFG1.1	AFG1	Accuracy	0.0313	48742.071	1.435e4	5.30	0.06	66.2	☐	0.245	782.15	0.4375	0.4396
59	Neat Standard_2	Standard	AFG1.1	AFG1	Accuracy	0.0625	93419.137	2.482e4	5.28	0.05	86.8	☐	0.532	850.68	0.4897	0.4396
111	Neat Standard_3	Standard	AFG1.1	AFG1		1.2500	179198.288	4.931e4	5.29	0.05	114.9	☑	1.082	86.60	0.4414	0.4396
163	Neat Standard_4	Standard	AFG1.1	AFG1		3.1250	511649.350	1.337e5	5.29	0.05	221.5	☑	3.217	102.94	0.4279	0.4396
215	Neat Standard_5	Standard	AFG1.1	AFG1		6.2500	967505.399	2.484e5	5.29	0.05	242.7	☑	6.144	98.30	0.4224	0.4396
267	Neat Standard_6	Standard	AFG1.1	AFG1		12.5000	1890120.0...	4.692e5	5.28	0.06	293.2	☑	12.068	96.54	0.4471	0.4396
319	Neat Standard_7	Standard	AFG1.1	AFG1		31.2500	5081295.9...	1.361e6	5.31	0.05	346.1	☑	32.558	104.18	0.4584	0.4396
371	Neat Standard_8	Standard	AFG1.1	AFG1		62.5000	10616376...	2.478e6	5.31	0.06	290.1	☑	68.097	108.96	0.4403	0.4396
423	Neat Standard_9	Standard	AFG1.1	AFG1	Accuracy	125.0000	N/A	N/A	N/A	N/A	N/A	☑	N/A	N/A	N/A	0.4396
475	Neat Standard_10	Standard	AFG1.1	AFG1		312.5000	52069235...	1.179e7	5.30	0.06	295.0	☑	334.256	106.96	0.4880	0.4396
527	Neat Standard_11	Standard	AFG1.1	AFG1		625.0000	92983124...	1.975e7	5.30	0.06	310.5	☑	596.954	95.51	0.4770	0.4396
579	Neat Standard_12	Standard	AFG1.1	AFG1	Accuracy	1250.0000	15379178...	2.981e7	5.30	0.07	305.1	☐	987.391	78.99	0.4974	0.4396

Regression

Calibration for AFG1.1: y = 1.55745e5 x + 10613.84479 (r = 0.99833) (weighting: 1 / x)

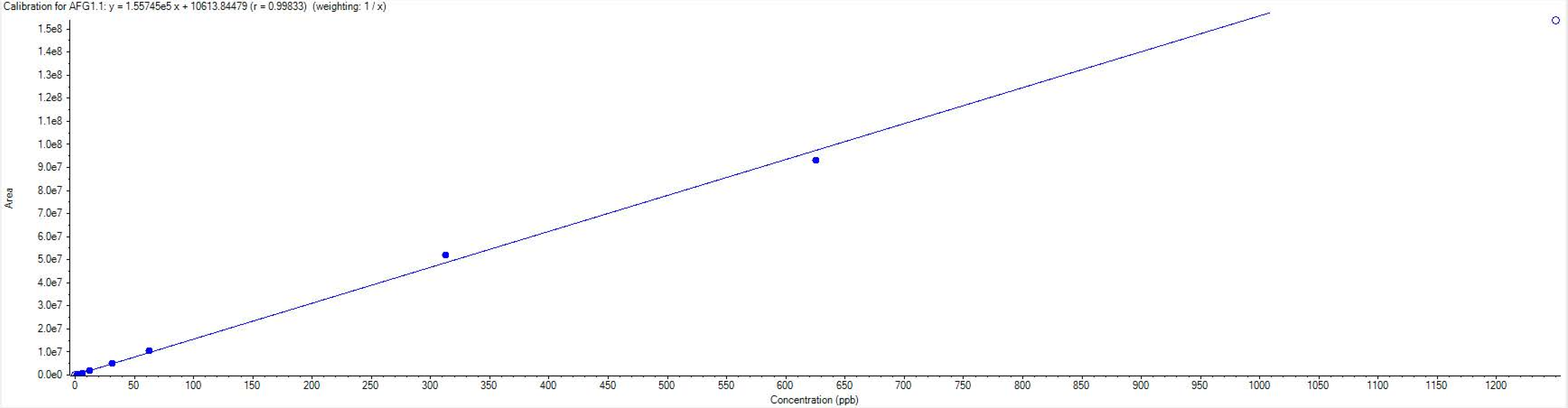

MultiQuant - [[MQ4] Results Table (Untitled)]

File Edit Process Window Help

Mycotoxins\2017

Components & Groups IS

All Components

3 Acetyl DON Group
AFB1 Group
AFB2 Group
AFG1 Group
AFG2 Group
Citrinin Group
DAS Group
DON Group
FB1 Group
FB2 Group
FX Group
HT2 Group
NEOS Group
NIV Group
OTA Group
Sterig Group
ZONE Group
15-AC Group
FB3 Group
T2 Group
CIT-neg Group
DON-neg Group
NIV-neg Group
ZON-neg Group
3AC-neg Group

3 Acetyl DON.1
3 Acetyl DON.2
AFB1.1
AFB1.2
AFB2.1
AFB2.2
AFG1.1
AFG1.2
AFG2.1
AFG2.2
Citrinin.1
Citrinin.2
DAS.1
DAS.2
DON.1
DON.2
FB1.1
FB1.2
FB2.1
FB2.2
FX.1
FX.2
HT2.1
HT2.2
NEOS.1
NEOS.2
NIV.1
NIV.2
OTA.1
OTA.2
Sterig.1
Sterig.2
ZONE.1
ZONE.2
FB3.1
FB3.3
15-AC.4
15-AC.5
T2.1
T2.2
CIT-neg.1
CIT-neg.2
DON-neg.1

All Sample Types

Index	Sample Name	Sample Type	Component Name	Component Group Name	Outlier Reasons	Actual Concentration	Area	Height	Retention Time	Width at 50%	Used	Calculated Concentration	Accuracy	Ion Ratio	Expected Ion Ratio
1	Neat Standard_1	Standard	3 Acetyl DON.1	3 Acetyl DON	Ion Ratio	0.0125	118246.772	3.927e4	4.90	0.05	☑	0.014	109.74	0.0079	0.7352
53	Neat Standard_2	Standard	3 Acetyl DON.1	3 Acetyl DON	Ion Ratio	0.0250	192867.502	6.092e4	4.88	0.05	☑	0.024	94.74	0.0118	0.7352
105	Neat Standard_3	Standard	3 Acetyl DON.1	3 Acetyl DON	Ion Ratio	0.0500	347737.437	1.142e5	4.88	0.05	☑	0.044	88.74	0.0079	0.7352
157	Neat Standard_4	Standard	3 Acetyl DON.1	3 Acetyl DON	Ion Ratio	0.1250	968808.426	3.070e5	4.89	0.05	☑	0.127	101.86	1.6651	0.7352
209	Neat Standard_5	Standard	3 Acetyl DON.1	3 Acetyl DON	Ion Ratio	0.2500	1869436.3...	6.153e5	4.88	0.05	☑	0.248	99.05	1.7009	0.7352
261	Neat Standard_6	Standard	3 Acetyl DON.1	3 Acetyl DON	Ion Ratio	0.5000	3822584.7...	1.178e6	4.88	0.05	☑	0.509	101.70	1.6128	0.7352
313	Neat Standard_7	Standard	3 Acetyl DON.1	3 Acetyl DON	Ion Ratio	1.2500	9471943.4...	3.106e6	4.90	0.05	☑	1.263	101.05	0.0018	0.7352
365	Neat Standard_8	Standard	3 Acetyl DON.1	3 Acetyl DON	Ion Ratio	2.5000	20017772...	6.457e6	4.89	0.05	☑	2.672	106.87	1.6064	0.7352
417	Neat Standard_9	Standard	3 Acetyl DON.1	3 Acetyl DON	Ion Ratio	5.0000	36043669...	1.081e7	4.94	0.05	☑	4.812	96.25	0.0020	0.7352
469	Neat Standard_10	Standard	3 Acetyl DON.1	3 Acetyl DON	Ion Ratio	12.5000	75506128...	2.140e7	4.89	0.05	☐	10.083	80.67	1.5822	0.7352
521	Neat Standard_11	Standard	3 Acetyl DON.1	3 Acetyl DON	Accurac...	25.0000	11718748...	3.193e7	4.89	0.06	☐	15.651	62.60	1.5306	0.7352
573	Neat Standard_12	Standard	3 Acetyl DON.1	3 Acetyl DON	Accurac...	50.0000	16685632...	4.256e7	4.89	0.06	☐	22.285	44.57	1.4499	0.7352

Regression

Calibration for 3 Acetyl DON.1: y = 7.48655e6 x + 15553.74562 (r = 0.99891) (weighting: 1 / x)

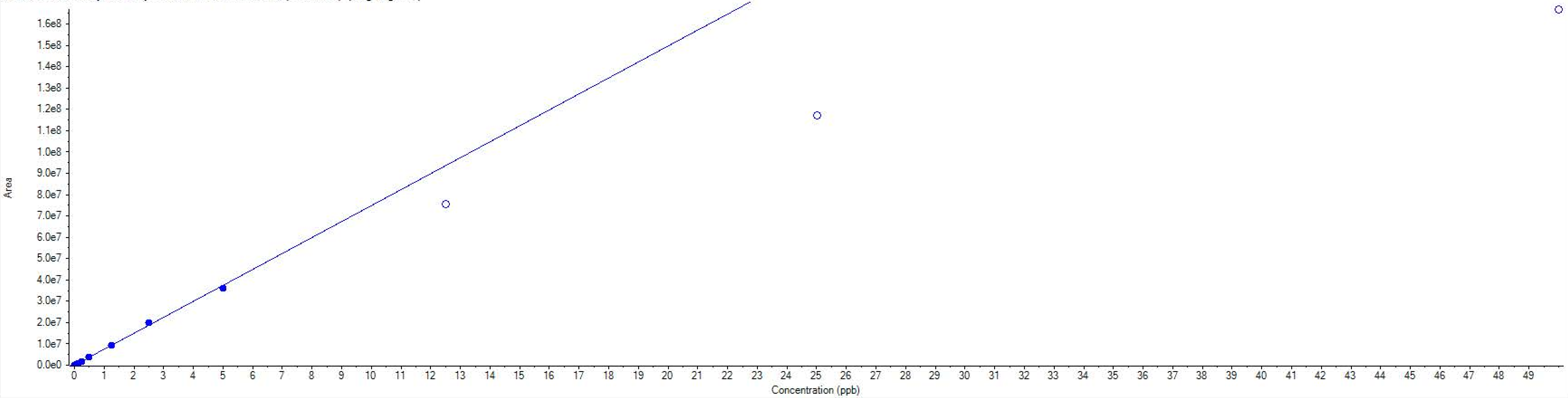

MultiQuant - [[MQ4] Results Table (Untitled)]

File Edit Process Window Help

Mycotoxins\2017

Components & Groups IS

All Components

3 Acetyl DON Group
AFB1 Group
AFB2 Group
AFG1 Group
AFG2 Group
Citrinin Group
DAS Group
DON Group
FB1 Group
FB2 Group
FX Group
HT2 Group
NEOS Group
NIV Group
OTA Group
Sterig Group
ZONE Group
FB3 Group
15-AC Group
T2 Group
CIT-neg Group
DON-neg Group
NIV-neg Group
ZON-neg Group
3AC-neg Group

3 Acetyl DON.1
3 Acetyl DON.2
AFB1.1
AFB1.2
AFB2.1
AFB2.2
AFG1.1
AFG1.2
AFG2.1
AFG2.2
Citrinin.1
Citrinin.2
DAS.1
DAS.2
DON.1
DON.2
FB1.1
FB1.2
FB2.1
FB2.2
FX.1
FX.2
HT2.1
HT2.2
NEOS.1
NEOS.2
NIV.1
NIV.2
OTA.1
OTA.2
Sterig.1
Sterig.2
ZONE.1
ZONE.2
FB3.1
FB3.3
15-AC.4
15-AC.5
T2.1
T2.2
CIT-neg.1
CIT-neg.2
DON-neg.1

All Sample Types

Index	Sample Name	Sample Type	Component Name	Component Group Name	Outlier Reasons	Actual Concentration	Area	Height	Retention Time	Width at 50%	Signal / Noise	Used	Calculated Concentration	Accuracy	Ion Ratio	Expected Ion Ratio
3	Neat Standard_1	Standard	AFB1.1	AFB1	Accuracy	0.0313	223533.131	3.814e4	6.00	0.08	131.5	☐	0.121	387.38	0.7430	0.7657
55	Neat Standard_2	Standard	AFB1.1	AFB1	Accuracy	0.0625	411640.666	6.855e4	5.98	0.07	199.0	☐	0.417	667.56	0.7485	0.7657
107	Neat Standard_3	Standard	AFB1.1	AFB1		1.2500	802486.279	1.381e5	5.99	0.07	264.2	☑	1.032	82.58	0.7410	0.7657
159	Neat Standard_4	Standard	AFB1.1	AFB1		3.1250	2036784.2...	3.441e5	6.00	0.08	309.5	☑	2.974	95.18	0.7948	0.7657
211	Neat Standard_5	Standard	AFB1.1	AFB1		6.2500	4002955.2...	6.923e5	6.00	0.07	325.5	☑	6.068	97.09	0.7755	0.7657
263	Neat Standard_6	Standard	AFB1.1	AFB1		12.5000	7957727.3...	1.333e6	5.99	0.08	324.0	☑	12.290	98.32	0.7774	0.7657
315	Neat Standard_7	Standard	AFB1.1	AFB1		31.2500	22414293....	3.651e6	6.02	0.08	334.3	☑	35.037	112.12	0.7707	0.7657
367	Neat Standard_8	Standard	AFB1.1	AFB1		62.5000	47659016....	7.155e6	6.02	0.08	402.8	☑	74.757	119.61	0.7348	0.7657
419	Neat Standard_9	Standard	AFB1.1	AFB1	Accurac...	125.0000	N/A	N/A	N/A	N/A	N/A	☑	N/A	N/A	N/A	0.7657
471	Neat Standard_10	Standard	AFB1.1	AFB1		312.5000	18904381...	3.202e7	6.00	0.08	362.8	☑	297.216	95.11	0.7894	0.7657
523	Neat Standard_11	Standard	AFB1.1	AFB1	Accuracy	625.0000	31756591...	4.945e7	6.00	0.09	392.2	☐	499.436	79.91	0.8035	0.7657
575	Neat Standard_12	Standard	AFB1.1	AFB1	Accuracy	1250.0000	48518639...	6.594e7	6.00	0.11	300.3	☐	763.174	61.05	0.8377	0.7657

Regression

Calibration for AFB1.1: y = 6.35556e5 x + 1.46473e5 (r = 0.99539) (weighting: 1 / x)

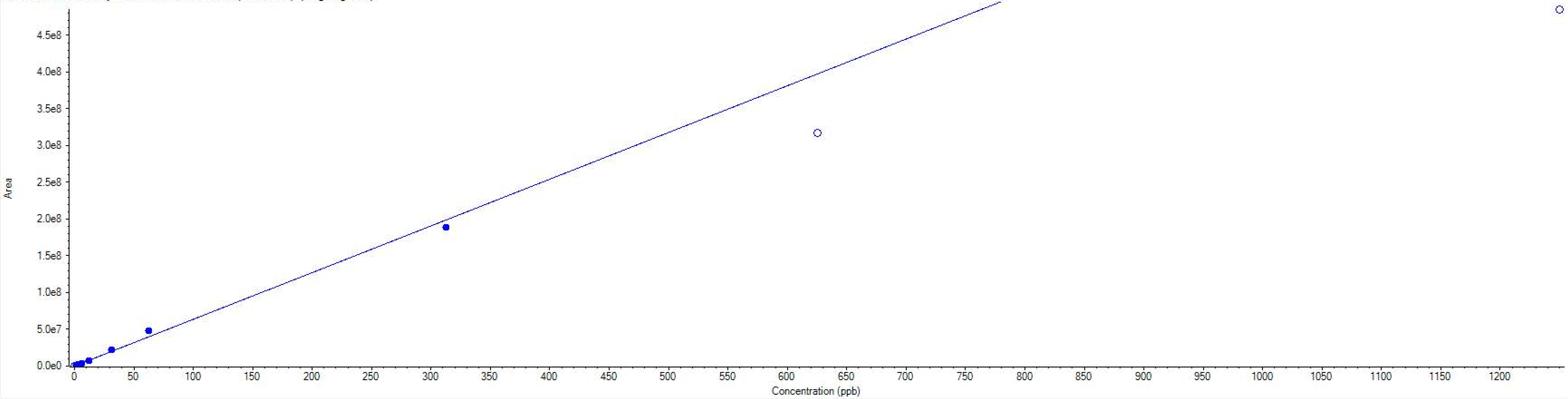

Supplement: Supplementary file 1 [file ijerph-23-00949-s001.zip › File S1 Method Validation Info_SG_2026.pdf]
