# Supplementary material for: Occurrence and Co-Occurrence of Regulated and Emerging Mycotoxins in Foods Marketed to U.S. Toddlers
Source: Int J Environ Res Public Health. 2026 Jul 23;23(8):949. doi: 10.3390/ijerph23080949 (PMC13512329; doi:10.3390/ijerph23080949)
Supplement: Supplementary file 1 [file ijerph-23-00949-s001.zip › File S2 LC-MSMS method uncertainty supplement.pdf]

Analytical Method Uncertainty Budget--3ACETYL DON LCMSMS

Uncertainty Source (Factor)	Estimated Value	Distribution Factor	Type of Uncertainty	Divisor	Method Uncertainty
**Volumetric pipette uncertainty**	+/- 0.1ml	Rectangular	B	1.732	0.06
**Tech pipetting repeatability**	0.20 %RSD	Normal	A	1	0.20
**Repeatability of HPLC system**	10.414 %RSD	Normal	A	1	10.41
**Tech pipetting repeatability 1-5ml**	0.71 %RSD	Normal	A	1	0.71
**Tech pipetting repeatability 200-1000µl**	0.58 %RSD	Normal	A	1	0.58
**Tech pipetting repeatability 20-200µl**	0.54 %RSD	Normal	A	1	0.54
**Tech repeatability 10-100ml dispensor**	0.25 %RSD	Normal	A	1	0.25
**Tech repeatability bench scale**	0.20 %RSD	Normal	A	1	0.20
**Combined Uncertainty**	10.48				
**Expanded Uncertainty**	20.95				

* Combined uncertainty is equal to the root sum square of all variables in the uncertainty budget after being adjusted using distribution factors to standard uncertainty form

**Expanded Uncertainty is equal to the combined uncertainty multiplied by a factor of two to give 95% confidence in the measurement of uncertainty

Calculations of uncertainty (k=2) are based on the criteria outlined in the ISO guide to the expression of uncertainty in measurement (GUM)

	4.9755	4.9532	4.9744
	4.9786	4.9635	4.9687
**Tech pipetting repeatability 30 repetitions using a 5ml ml A class Volumetric pipette**	4.9758	4.9602	4.9790
	4.9772	4.9695	4.9629
	4.9766	4.9504	4.9782
	4.9613	4.9541	4.9826
	4.9532	4.9592	4.9734
	4.9640	4.9794	4.9672
	4.9630	4.9598	4.9686
	4.9850	4.9521	4.9720
		**Standard deviation**	0.01
		**MEAN**	4.97
		**%RSD**	0.20

	82	86	84
	87	108	77
**LCMSMS repeatability 30 repetitions**	115	101	88
	91	89	91
	78	115	115
	85	117	93
	75	111	76
	104	91	71
	90	106	77
	114	74	100
		**Standard Deviation**	14.42
		**MEAN**	93.03
		**%RSD**	15.498

	4968.7000	4885.4000	4950.0000
	4922.1000	4974.5000	4924.8000
**Tech pipetting repeatability 30 repetitions using a 5ml pipette**	4937.5000	4999.1000	4942.8000
	4929.2000	4971.6000	4954.1000
	4941.1000	4991.1000	4947.3000
	4888.1000	4984.7000	4951.7000
	4911.4000	4967.4000	4938.4000
	4889.6000	4997.7000	4949.0000
	4867.3000	4949.6000	4897.4000
	4894.4000	4975.8000	4933.5000
		**Standard deviation**	35.25
		**MEAN**	4941.18
		**%RSD**	0.71

	998.4000	992.3000	1002.4000
	999.6000	997.8000	997.6000
**Tech pipetting repeatability 30 repetitions using a 200-1000µl pipette**	995.0000	997.9000	999.1000
	993.1000	995.5000	996.8000
	986.2000	989.7000	984.1000
	991.6000	996.5000	1001.2000
	980.5000	991.5000	997.2000
	992.2000	999.1000	997.6000
	991.3000	998.6000	1000.7000
	990.1000	980.2000	998.1000
		**Standard deviation**	5.81
		**MEAN**	994.40
		**%RSD**	0.58

	199.8000	197.9000	201.5000
	200.1000	198.9000	201.0000
**Tech pipetting repeatability 30 repetitions using a 20-200µl pipette**	200.3000	197.2000	201.3000
	199.5000	201.6000	201.2000
	199.6000	199.4000	201.0000
	199.8000	199.3000	201.1000
	200.9000	200.8000	201.2000
	199.0000	199.6000	200.9000
	200.0000	201.2000	201.0000
	199.6000	199.4000	201.1000
		**Standard deviation**	1.09
		**MEAN**	200.17
		**%RSD**	0.54

	49452.6000	49184.7000	49195.1000
	49432.1000	49130.8000	49273.6000
**Tech repeatability 30 repetitions using a 10-100ml dispensor**	49493.0000	49412.6000	49261.8000
	49450.7000	49523.6000	49252.6000
	49373.9000	49405.0000	49115.0000
	49387.7000	49443.8000	49169.4000
	49474.1000	49354.0000	49189.3000
	49357.5000	49443.3000	49135.2000
	49367.6000	49402.4000	49251.1000
	49372.6000	49453.0000	49194.4000
		**Standard deviation**	123.23
		**MEAN**	49331.75
		**%RSD**	0.25

	200.0000	200.1000	200.0000
	200.0000	200.0000	200.0000
**Tech repeatability 30 repetitions using a bench scale S/N 19353802**	200.0000	200.0000	200.0000
	200.1000	200.0000	200.0000
	200.0000	200.0000	200.1000
	200.0000	200.0000	200.0000
	200.0000	200.0000	200.0000
	200.0000	200.0000	200.0000
	200.0000	200.0000	200.0000
	200.1000	200.0000	200.0000
		**Standard deviation**	0.03
		**MEAN**	200.01
		**%RSD**	0.02

REVISION HISTORY

REVISED DATE	REVISED BY	CHANGES MADE
6/22/2016	Julie Brunkhorst	Orginal Document

Analytical Method Uncertainty Budget--15ACETYL DON LCMSMS

Uncertainty Source (Factor)	Estimated Value	Distribution Factor	Type of Uncertainty	Divisor	Method Uncertainty
**Volumetric pipette uncertainty**	+/- 0.1ml	Rectangular	B	1.732	0.06
**Tech pipetting repeatability**	0.20 %RSD	Normal	A	1	0.20
**Repeatability of HPLC system**	10.414 %RSD	Normal	A	1	10.41
**Tech pipetting repeatability 1-5ml**	0.71 %RSD	Normal	A	1	0.71
**Tech pipetting repeatability 200-1000µl**	0.58 %RSD	Normal	A	1	0.58
**Tech pipetting repeatability 20-200µl**	0.54 %RSD	Normal	A	1	0.54
**Tech repeatability 10-100ml dispensor**	0.25 %RSD	Normal	A	1	0.25
**Tech repeatability bench scale**	0.20 %RSD	Normal	A	1	0.20
**Combined Uncertainty**	10.48				
**Expanded Uncertainty**	20.95				

* Combined uncertainty is equal to the root sum square of all variables in the uncertainty budget after being adjusted using distribution factors to standard uncertainty form

**Expanded Uncertainty is equal to the combined uncertainty multiplied by a factor of two to give 95% confidence in the measurement of uncertainty

Calculations of uncertainty (k=2) are based on the criteria outlined in the ISO guide to the expression of uncertainty in measurement (GUM)

	4.9755	4.9532	4.9744
	4.9786	4.9635	4.9687
**Tech pipetting repeatability 30 repetitions using a 5ml ml A class Volumetric pipette**	4.9758	4.9602	4.9790
	4.9772	4.9695	4.9629
	4.9766	4.9504	4.9782
	4.9613	4.9541	4.9826
	4.9532	4.9592	4.9734
	4.9640	4.9794	4.9672
	4.9630	4.9598	4.9686
	4.9850	4.9521	4.9720
		**Standard deviation**	0.01
		**MEAN**	4.97
		**%RSD**	0.20

	99	99	101
	84	98	101
**LCMSMS repeatability 30 repetitions**	91	118	84
	99	78	87
	108	92	80
	104	75	90
	83	115	91
	74	95	82
	83	92	98
	93	81	80
		**Standard Deviation**	11.19
		**MEAN**	91.83
		**%RSD**	12.190

	4968.7000	4885.4000	4950.0000
	4922.1000	4974.5000	4924.8000
**Tech pipetting repeatability 30 repetitions using a 5ml pipette**	4937.5000	4999.1000	4942.8000
	4929.2000	4971.6000	4954.1000
	4941.1000	4991.1000	4947.3000
	4888.1000	4984.7000	4951.7000
	4911.4000	4967.4000	4938.4000
	4889.6000	4997.7000	4949.0000
	4867.3000	4949.6000	4897.4000
	4894.4000	4975.8000	4933.5000
		**Standard deviation**	35.25
		**MEAN**	4941.18
		**%RSD**	0.71

	998.4000	992.3000	1002.4000
	999.6000	997.8000	997.6000
**Tech pipetting repeatability 30 repetitions using a 200-1000µl pipette**	995.0000	997.9000	999.1000
	993.1000	995.5000	996.8000
	986.2000	989.7000	984.1000
	991.6000	996.5000	1001.2000
	980.5000	991.5000	997.2000
	992.2000	999.1000	997.6000
	991.3000	998.6000	1000.7000
	990.1000	980.2000	998.1000
		**Standard deviation**	5.81
		**MEAN**	994.40
		**%RSD**	0.58

	199.8000	197.9000	201.5000
	200.1000	198.9000	201.0000
**Tech pipetting repeatability 30 repetitions using a 20-200µl pipette**	200.3000	197.2000	201.3000
	199.5000	201.6000	201.2000
	199.6000	199.4000	201.0000
	199.8000	199.3000	201.1000
	200.9000	200.8000	201.2000
	199.0000	199.6000	200.9000
	200.0000	201.2000	201.0000
	199.6000	199.4000	201.1000
		**Standard deviation**	1.09
		**MEAN**	200.17
		**%RSD**	0.54

	49452.6000	49184.7000	49195.1000
	49432.1000	49130.8000	49273.6000
**Tech repeatability 30 repetitions using a 10-100ml dispensor**	49493.0000	49412.6000	49261.8000
	49450.7000	49523.6000	49252.6000
	49373.9000	49405.0000	49115.0000
	49387.7000	49443.8000	49169.4000
	49474.1000	49354.0000	49189.3000
	49357.5000	49443.3000	49135.2000
	49367.6000	49402.4000	49251.1000
	49372.6000	49453.0000	49194.4000
		**Standard deviation**	123.23
		**MEAN**	49331.75
		**%RSD**	0.25

	200.0000	200.1000	200.0000
	200.0000	200.0000	200.0000
**Tech repeatability 30 repetitions using a bench scale S/N 19353802**	200.0000	200.0000	200.0000
	200.1000	200.0000	200.0000
	200.0000	200.0000	200.1000
	200.0000	200.0000	200.0000
	200.0000	200.0000	200.0000
	200.0000	200.0000	200.0000
	200.0000	200.0000	200.0000
	200.1000	200.0000	200.0000
		**Standard deviation**	0.03
		**MEAN**	200.01
		**%RSD**	0.02

REVISION HISTORY

REVISED DATE	REVISED BY	CHANGES MADE
6/22/2016	Julie Brunkhorst	Orginal Document

Analytical Method Uncertainty Budget--Afla LCMSMS 2019

Uncertainty Source (Factor)	Estimated Value	Distribution Factor	Type of Uncertainty	Divisor	Method Uncertainty
**Purity**	99.5 % (+/- 0.5 %)	Rectangular	B	1.732	0.29
**Amount of Crystalline**	+/- 0.1mg	Rectangular	B	1.732	0.06
**Volumetric pipette uncertainty**	+/- 0.1ml	Rectangular	B	1.732	0.06
**Tech pipetting repeatability**	0.20 %RSD	Normal	A	1	0.20
**Repeatability of UV-Spectrophotometer**	0.145 %RSD	Normal	A	1	0.15
**Repeatability of HPLC system**	7.476 %RSD	Normal	A	1	7.48
**Uncertainty of Gold STD (sigma)**	1.0 ug/ml	Rectangular	B	1.732	0.58
**Tech pipetting repeatability 1-5ml**	0.71 %RSD	Normal	A	1	0.71
**Tech pipetting repeatability 200-1000µl**	0.58 %RSD	Normal	A	1	0.58
**Tech pipetting repeatability 20-200µl**	0.54 %RSD	Normal	A	1	0.54
**Tech repeatability 10-100ml dispensor**	0.25 %RSD	Normal	A	1	0.25
**Tech repeatability bench scale**	0.20 %RSD	Normal	A	1	0.20
**Combined Uncertainty**	7.59				
**Expanded Uncertainty**	15.18				

* Combined uncertainty is equal to the root sum square of all variables in the uncertainty budget after being adjusted using distribution factors to standard uncertainty form

**Expanded Uncertainty is equal to the combined uncertainty multiplied by a factor of two to give 95% confidence in the measurement of uncertainty

Calculations of uncertainty (k=2) are based on the criteria outlined in the ISO guide to the expression of uncertainty in measurement (GUM)

Tech pipetting repeatability 30 repetitions using a 5ml ml A class Volumetric pipette

4.9755	4.9532	4.9744
4.9786	4.9635	4.9687
4.9758	4.9602	4.9790
4.9772	4.9695	4.9629
4.9766	4.9504	4.9782
4.9613	4.9541	4.9826
4.9532	4.9592	4.9734
4.9640	4.9794	4.9672
4.9630	4.9598	4.9686
4.9850	4.9521	4.9720
	**Standard deviation**	0.01
	**MEAN**	4.97
	**%RSD**	0.20

UV - Spectrophotometer repeatability 30 repetitions

1.6754	1.6768	1.6795
1.6742	1.6799	1.6795
1.6775	1.6785	1.6821
1.6756	1.6779	1.6813
1.6767	1.6797	1.6802
1.6771	1.6789	1.6817
1.6773	1.6779	1.6802
1.6757	1.6821	1.6820
1.6749	1.6787	1.6771
1.6754	1.6806	1.6830
	**Standard deviation**	0.002
	**MEAN**	1.68
	**%RSD**	0.145

LCMSMS repeatability 30 repetitions

13.32	10.01	10.58
9.27	9.91	10.17
10.93	9.81	10.47
10.92	9.62	11.41
10.55	11.34	9.7
10.98	12.64	10.51
9.76	10.33	13.16
11.9	11.82	13.78
10.37	11.13	9.22
10.1	10.01	10.77
	**Standard Deviation**	1.18
	**MEAN**	10.82
	**%RSD**	10.913

	4968.7000	4885.4000	4950.0000
	4922.1000	4974.5000	4924.8000
**Tech pipetting repeatability**	4937.5000	4999.1000	4942.8000
**30 repetitions using a 5ml pipette**	4929.2000	4971.6000	4954.1000
	4941.1000	4991.1000	4947.3000
	4888.1000	4984.7000	4951.7000
	4911.4000	4967.4000	4938.4000
	4889.6000	4997.7000	4949.0000
	4867.3000	4949.6000	4897.4000
	4894.4000	4975.8000	4933.5000
		**Standard deviation**	35.25
		**MEAN**	4941.18
		**%RSD**	0.71

	998.4000	992.3000	1002.4000
	999.6000	997.8000	997.6000
**Tech pipetting repeatability**	995.0000	997.9000	999.1000
**30 repetitions using a 200-1000µl pipette**	993.1000	995.5000	996.8000
	986.2000	989.7000	984.1000
	991.6000	996.5000	1001.2000
	980.5000	991.5000	997.2000
	992.2000	999.1000	997.6000
	991.3000	998.6000	1000.7000
	990.1000	980.2000	998.1000
		**Standard deviation**	5.81
		**MEAN**	994.40
		**%RSD**	0.58

	199.8000	197.9000	201.5000
	200.1000	198.9000	201.0000
**Tech pipetting repeatability**	200.3000	197.2000	201.3000
**30 repetitions using a 20-200µl pipette**	199.5000	201.6000	201.2000
	199.6000	199.4000	201.0000
	199.8000	199.3000	201.1000
	200.9000	200.8000	201.2000
	199.0000	199.6000	200.9000
	200.0000	201.2000	201.0000
	199.6000	199.4000	201.1000
		**Standard deviation**	1.09
		**MEAN**	200.17
		**%RSD**	0.54

	49452.6000	49184.7000	49195.1000
	49432.1000	49130.8000	49273.6000
**Tech repeatability**	49493.0000	49412.6000	49261.8000
**30 repetitions using a 10-100ml dispensor**	49450.7000	49523.6000	49252.6000
	49373.9000	49405.0000	49115.0000
	49387.7000	49443.8000	49169.4000
	49474.1000	49354.0000	49189.3000
	49357.5000	49443.3000	49135.2000
	49367.6000	49402.4000	49251.1000
	49372.6000	49453.0000	49194.4000
		**Standard deviation**	123.23
		**MEAN**	49331.75
		**%RSD**	0.25

	200.0000	200.1000	200.0000
	200.0000	200.0000	200.0000
**Tech repeatability**	200.0000	200.0000	200.0000
**30 repetitions using a bench scale S/N 19353802**	200.1000	200.0000	200.0000
	200.0000	200.0000	200.1000
	200.0000	200.0000	200.0000
	200.0000	200.0000	200.0000
	200.0000	200.0000	200.0000
	200.0000	200.0000	200.0000
	200.1000	200.0000	200.0000
		**Standard deviation**	0.03
		**MEAN**	200.01
		**%RSD**	0.02

REVISION HISTORY

REVISED DATE	REVISED BY	CHANGES MADE
6/22/2016	Julie Brunkhorst	Orginal Document

Analytical Method Uncertainty Budget--FUM LCMSMS 2019

Uncertainty Source (Factor)	Estimated Value	Distribution Factor	Type of Uncertainty	Divisor	Method Uncertainty
**Purity**	99.5 % (+/- 0.5 %)	Rectangular	B	1.732	0.29
**Amount of Crystalline**	+/- 0.1mg	Rectangular	B	1.732	0.06
**Volumetric pipette uncertainty**	+/- 0.1ml	Rectangular	B	1.732	0.06
**Tech pipetting repeatability**	0.20 %RSD	Normal	A	1	0.20
**Repeatability of UV-Spectrophotometer**	0.145 %RSD	Normal	A	1	0.15
**Repeatability of HPLC system**	7.446 %RSD	Normal	A	1	7.45
**Uncertainty of Gold STD (sigma)**	1.0 ug/ml	Rectangular	B	1.732	0.58
**Tech pipetting repeatability 1-5ml**	0.71 %RSD	Normal	A	1	0.71
**Tech pipetting repeatability 200-1000µl**	0.58 %RSD	Normal	A	1	0.58
**Tech pipetting repeatability 20-200µl**	0.54 %RSD	Normal	A	1	0.54
**Tech repeatability 10-100ml dispensor**	0.25 %RSD	Normal	A	1	0.25
**Tech repeatability bench scale**	0.20 %RSD	Normal	A	1	0.20
**Combined Uncertainty**	7.56				
**Expanded Uncertainty**	15.12				

* Combined uncertainty is equal to the root sum square of all variables in the uncertainty budget after being adjusted using distribution factors to standard uncertainty form

**Expanded Uncertainty is equal to the combined uncertainty multiplied by a factor of two to give 95% confidence in the measurement of uncertainty

Calculations of uncertainty (k=2) are based on the criteria outlined in the ISO guide to the expression of uncertainty in measurement (GUM)

Tech pipetting repeatability 30 repetitions using a 5ml ml A class Volumetric pipette

4.9755	4.9532	4.9744
4.9786	4.9635	4.9687
4.9758	4.9602	4.9790
4.9772	4.9695	4.9629
4.9766	4.9504	4.9782
4.9613	4.9541	4.9826
4.9532	4.9592	4.9734
4.9640	4.9794	4.9672
4.9630	4.9598	4.9686
4.9850	4.9521	4.9720
	**Standard deviation**	0.01
	**MEAN**	4.97
	**%RSD**	0.20

UV - Spectrophotometer repeatability 30 repetitions

1.6754	1.6768	1.6795
1.6742	1.6799	1.6795
1.6775	1.6785	1.6821
1.6756	1.6779	1.6813
1.6767	1.6797	1.6802
1.6771	1.6789	1.6817
1.6773	1.6779	1.6802
1.6757	1.6821	1.6820
1.6749	1.6787	1.6771
1.6754	1.6806	1.6830
	**Standard deviation**	0.002
	**MEAN**	1.68
	**%RSD**	0.145

LCMSMS repeatability 30 repetitions

3.51	3.74	2.97
3.28	3.44	2.9
3.13	3.06	2.66
3.98	3.6	2.74
3.21	3.71	3.43
3.67	2.95	2.83
3.68	3.4	3.79
3.26	3.58	3.41
3.18	2.89	3.26
3.39	3.11	3.51
	**Standard Deviation**	0.34
	**MEAN**	3.31
	**%RSD**	10.223

	4968.7000	4885.4000	4950.0000
	4922.1000	4974.5000	4924.8000
**Tech pipetting repeatability 30 repetitions using a 5ml pipette**	4937.5000	4999.1000	4942.8000
	4929.2000	4971.6000	4954.1000
	4941.1000	4991.1000	4947.3000
	4888.1000	4984.7000	4951.7000
	4911.4000	4967.4000	4938.4000
	4889.6000	4997.7000	4949.0000
	4867.3000	4949.6000	4897.4000
	4894.4000	4975.8000	4933.5000
		**Standard deviation**	35.25
		**MEAN**	4941.18
		**%RSD**	0.71

	998.4000	992.3000	1002.4000
	999.6000	997.8000	997.6000
**Tech pipetting repeatability 30 repetitions using a 200-1000µl pipette**	995.0000	997.9000	999.1000
	993.1000	995.5000	996.8000
	986.2000	989.7000	984.1000
	991.6000	996.5000	1001.2000
	980.5000	991.5000	997.2000
	992.2000	999.1000	997.6000
	991.3000	998.6000	1000.7000
	990.1000	980.2000	998.1000
		**Standard deviation**	5.81
		**MEAN**	994.40
		**%RSD**	0.58

	199.8000	197.9000	201.5000
	200.1000	198.9000	201.0000
**Tech pipetting repeatability 30 repetitions using a 20-200µl pipette**	200.3000	197.2000	201.3000
	199.5000	201.6000	201.2000
	199.6000	199.4000	201.0000
	199.8000	199.3000	201.1000
	200.9000	200.8000	201.2000
	199.0000	199.6000	200.9000
	200.0000	201.2000	201.0000
	199.6000	199.4000	201.1000
		**Standard deviation**	1.09
		**MEAN**	200.17
		**%RSD**	0.54

	49452.6000	49184.7000	49195.1000
	49432.1000	49130.8000	49273.6000
**Tech repeatability 30 repetitions using a 10-100ml dispensor**	49493.0000	49412.6000	49261.8000
	49450.7000	49523.6000	49252.6000
	49373.9000	49405.0000	49115.0000
	49387.7000	49443.8000	49169.4000
	49474.1000	49354.0000	49189.3000
	49357.5000	49443.3000	49135.2000
	49367.6000	49402.4000	49251.1000
	49372.6000	49453.0000	49194.4000
		**Standard deviation**	123.23
		**MEAN**	49331.75
		**%RSD**	0.25

	200.0000	200.1000	200.0000
	200.0000	200.0000	200.0000
**Tech repeatability 30 repetitions using a bench scale S/N 19353802**	200.0000	200.0000	200.0000
	200.1000	200.0000	200.0000
	200.0000	200.0000	200.1000
	200.0000	200.0000	200.0000
	200.0000	200.0000	200.0000
	200.0000	200.0000	200.0000
	200.0000	200.0000	200.0000
	200.1000	200.0000	200.0000
		**Standard deviation**	0.03
		**MEAN**	200.01
		**%RSD**	0.02

REVISION HISTORY

REVISED DATE	REVISED BY	CHANGES MADE
6/22/2016	Julie Brunkhorst	Orginal Document

Analytical Method Uncertainty Budget--HT2 LCMSMS 2019

Uncertainty Source (Factor)	Estimated Value	Distribution Factor	Type of Uncertainty	Divisor	Method Uncertainty
**Volumetric pipette uncertainty**	+/- 0.1ml	Rectangular	B	1.732	0.06
**Tech pipetting repeatability**	0.20 %RSD	Normal	A	1	0.20
**Repeatability of LCMSMS system**	13.182 %RSD	Normal	A	1	13.18
**Tech pipetting repeatability 1-5ml**	0.71 %RSD	Normal	A	1	0.71
**Tech pipetting repeatability 200-1000µl**	0.58 %RSD	Normal	A	1	0.58
**Tech pipetting repeatability 20-200µl**	0.54 %RSD	Normal	A	1	0.54
**Tech repeatability 10-100ml dispensor**	0.25 %RSD	Normal	A	1	0.25
**Tech repeatability bench scale**	0.20 %RSD	Normal	A	1	0.20
**Combined Uncertainty**	13.23				
**Expanded Uncertainty**	26.46				

* Combined uncertainty is equal to the root sum square of all variables in the uncertainty budget after being adjusted using distribution factors to standard uncertainty form

**Expanded Uncertainty is equal to the combined uncertainty multiplied by a factor of two to give 95% confidence in the measurement of uncertainty

Calculations of uncertainty (k=2) are based on the criteria outlined in the ISO guide to the expression of uncertainty in measurement (GUM)

	4.9755	4.9532	4.9744
	4.9786	4.9635	4.9687
**Tech pipetting repeatability 30 repetitions using a 5ml ml A class Volumetric pipette**	4.9758	4.9602	4.9790
	4.9772	4.9695	4.9629
	4.9766	4.9504	4.9782
	4.9613	4.9541	4.9826
	4.9532	4.9592	4.9734
	4.9640	4.9794	4.9672
	4.9630	4.9598	4.9686
	4.9850	4.9521	4.9720
		**Standard deviation**	0.01
		**MEAN**	4.97
		**%RSD**	0.20

	149.55	224.69	201.32
	186.57	223.9	147.78
**LCMSMS repeatability 30 repetitions**	134.49	193.67	175.45
	172.9	194.99	191.48
	149.16	203.81	182.81
	196.46	188.7	188.22
	220.54	201.83	128.23
	151.58	181.19	175.25
	158.31	172.54	204.33
	219.94	202.33	206.59
		**Standard Deviation**	26.27
		**MEAN**	184.29
		**%RSD**	14.255

Tech pipetting repeatability 30 repetitions using a 5ml pipette			
	4968.7000	4885.4000	4950.0000
	4922.1000	4974.5000	4924.8000
	4937.5000	4999.1000	4942.8000
	4929.2000	4971.6000	4954.1000
	4941.1000	4991.1000	4947.3000
	4888.1000	4984.7000	4951.7000
	4911.4000	4967.4000	4938.4000
	4889.6000	4997.7000	4949.0000
	4867.3000	4949.6000	4897.4000
	4894.4000	4975.8000	4933.5000
		**Standard deviation**	35.25
		**MEAN**	4941.18
		**%RSD**	0.71

Tech pipetting repeatability 30 repetitions using a 200-1000µl pipette			
	998.4000	992.3000	1002.4000
	999.6000	997.8000	997.6000
	995.0000	997.9000	999.1000
	993.1000	995.5000	996.8000
	986.2000	989.7000	984.1000
	991.6000	996.5000	1001.2000
	980.5000	991.5000	997.2000
	992.2000	999.1000	997.6000
	991.3000	998.6000	1000.7000
	990.1000	980.2000	998.1000
		**Standard deviation**	5.81
		**MEAN**	994.40
		**%RSD**	0.58

Tech pipetting repeatability 30 repetitions using a 20-200µl pipette			
	199.8000	197.9000	201.5000
	200.1000	198.9000	201.0000
	200.3000	197.2000	201.3000
	199.5000	201.6000	201.2000
	199.6000	199.4000	201.0000
	199.8000	199.3000	201.1000
	200.9000	200.8000	201.2000
	199.0000	199.6000	200.9000
	200.0000	201.2000	201.0000
	199.6000	199.4000	201.1000
		**Standard deviation**	1.09
		**MEAN**	200.17
		**%RSD**	0.54

Tech repeatability 30 repetitions using a 10-100ml dispensor			
	49452.6000	49184.7000	49195.1000
	49432.1000	49130.8000	49273.6000
	49493.0000	49412.6000	49261.8000
	49450.7000	49523.6000	49252.6000
	49373.9000	49405.0000	49115.0000
	49387.7000	49443.8000	49169.4000
	49474.1000	49354.0000	49189.3000
	49357.5000	49443.3000	49135.2000
	49367.6000	49402.4000	49251.1000
	49372.6000	49453.0000	49194.4000
		**Standard deviation**	123.23
		**MEAN**	49331.75
		**%RSD**	0.25

Tech repeatability 30 repetitions using a bench scale S/N 19353802			
	200.0000	200.1000	200.0000
	200.0000	200.0000	200.0000
	200.0000	200.0000	200.0000
	200.1000	200.0000	200.0000
	200.0000	200.0000	200.1000
	200.0000	200.0000	200.0000
	200.0000	200.0000	200.0000
	200.0000	200.0000	200.0000
	200.0000	200.0000	200.0000
	200.1000	200.0000	200.0000
		**Standard deviation**	0.03
		**MEAN**	200.01
		**%RSD**	0.02

REVISION HISTORY

REVISED DATE	REVISED BY	CHANGES MADE
6/22/2016	Julie Brunkhorst	Orginal Document

Analytical Method Uncertainty Budget--OTA LCMSMS

Uncertainty Source (Factor)	Estimated Value	Distribution Factor	Type of Uncertainty	Divisor	Method Uncertainty
**Purity**	99.5 % (+/- 0.5 %)	Rectangular	B	1.732	0.29
**Amount of Crystalline**	+/- 0.1mg	Rectangular	B	1.732	0.06
**Volumetric pipette uncertainty**	+/- 0.1ml	Rectangular	B	1.732	0.06
**Tech pipetting repeatability**	0.20 %RSD	Normal	A	1	0.20
**Repeatability of UV-Spectrophotometer**	0.145 %RSD	Normal	A	1	0.15
**Repeatability of HPLC system**	18.095 %RSD	Normal	A	1	18.10
**Uncertainty of Gold STD (sigma)**	1.0 ug/ml	Rectangular	B	1.732	0.58
**Tech pipetting repeatability 1-5ml**	0.71 %RSD	Normal	A	1	0.71
**Tech pipetting repeatability 200-1000µl**	0.58 %RSD	Normal	A	1	0.58
**Tech pipetting repeatability 20-200µl**	0.54 %RSD	Normal	A	1	0.54
**Tech repeatability 10-100ml dispensor**	0.25 %RSD	Normal	A	1	0.25
**Tech repeatability bench scale**	0.20 %RSD	Normal	A	1	0.20
**Combined Uncertainty**	18.14				
**Expanded Uncertainty**	36.28				

* Combined uncertainty is equal to the root sum square of all variables in the uncertainty budget after being adjusted using distribution factors to standard uncertainty form

**Expanded Uncertainty is equal to the combined uncertainty multiplied by a factor of two to give 95% confidence in the measurement of uncertainty

Calculations of uncertainty (k=2) are based on the criteria outlined in the ISO guide to the expression of uncertainty in measurement (GUM)

	4.9755	4.9532	4.9744
	4.9786	4.9635	4.9687
**Tech pipetting repeatability 30 repetitions using a 5ml ml A class Volumetric pipette**	4.9758	4.9602	4.9790
	4.9772	4.9695	4.9629
	4.9766	4.9504	4.9782
	4.9613	4.9541	4.9826
	4.9532	4.9592	4.9734
	4.9640	4.9794	4.9672
	4.9630	4.9598	4.9686
	4.9850	4.9521	4.9720
		**Standard deviation**	0.01
		**MEAN**	4.97
		**%RSD**	0.20

	1.6754	1.6768	1.6795
	1.6742	1.6799	1.6795
**UV - Spectrophotometer repeatability 30 repetitions**	1.6775	1.6785	1.6821
	1.6756	1.6779	1.6813
	1.6767	1.6797	1.6802
	1.6771	1.6789	1.6817
	1.6773	1.6779	1.6802
	1.6757	1.6821	1.6820
	1.6749	1.6787	1.6771
	1.6754	1.6806	1.6830
		**Standard deviation**	0.002
		**MEAN**	1.68
		**%RSD**	0.145

	3.92	3.88	4.47
	3.89	2.45	5.35
**HPLC repeatability 30 repetitions**	4.61	5.22	4.06
	3.51	4.95	4.4
	3.46	4.88	3.44
	4.77	3.62	4.54
	2.65	4.43	5.05
	4.59	5.01	3.31
	5	4.13	5.02
	5.02	3.7	5.33
		**Standard Deviation**	0.78
		**MEAN**	4.29
		**%RSD**	18.095

Tech pipetting repeatability
30 repetitions using a 5ml pipette

4968.7000	4885.4000	4950.0000
4922.1000	4974.5000	4924.8000
4937.5000	4999.1000	4942.8000
4929.2000	4971.6000	4954.1000
4941.1000	4991.1000	4947.3000
4888.1000	4984.7000	4951.7000
4911.4000	4967.4000	4938.4000
4889.6000	4997.7000	4949.0000
4867.3000	4949.6000	4897.4000
4894.4000	4975.8000	4933.5000
	**Standard deviation**	35.25
	**MEAN**	4941.18
	**%RSD**	0.71

Tech pipetting repeatability
30 repetitions using a 200-1000µl pipette

998.4000	992.3000	1002.4000
999.6000	997.8000	997.6000
995.0000	997.9000	999.1000
993.1000	995.5000	996.8000
986.2000	989.7000	984.1000
991.6000	996.5000	1001.2000
980.5000	991.5000	997.2000
992.2000	999.1000	997.6000
991.3000	998.6000	1000.7000
990.1000	980.2000	998.1000
	**Standard deviation**	5.81
	**MEAN**	994.40
	**%RSD**	0.58

Tech pipetting repeatability
30 repetitions using a 20-200µl pipette

199.8000	197.9000	201.5000
200.1000	198.9000	201.0000
200.3000	197.2000	201.3000
199.5000	201.6000	201.2000
199.6000	199.4000	201.0000
199.8000	199.3000	201.1000
200.9000	200.8000	201.2000
199.0000	199.6000	200.9000
200.0000	201.2000	201.0000
199.6000	199.4000	201.1000
	**Standard deviation**	1.09
	**MEAN**	200.17
	**%RSD**	0.54

Tech repeatability
30 repetitions using a 10-100ml dispensor

49452.6000	49184.7000	49195.1000
49432.1000	49130.8000	49273.6000
49493.0000	49412.6000	49261.8000
49450.7000	49523.6000	49252.6000
49373.9000	49405.0000	49115.0000
49387.7000	49443.8000	49169.4000
49474.1000	49354.0000	49189.3000
49357.5000	49443.3000	49135.2000
49367.6000	49402.4000	49251.1000
49372.6000	49453.0000	49194.4000
	**Standard deviation**	123.23
	**MEAN**	49331.75
	**%RSD**	0.25

Tech repeatability
30 repetitions using a bench scale S/N 19353802

200.0000	200.1000	200.0000
200.0000	200.0000	200.0000
200.0000	200.0000	200.0000
200.1000	200.0000	200.0000
200.0000	200.0000	200.1000
200.0000	200.0000	200.0000
200.0000	200.0000	200.0000
200.0000	200.0000	200.0000
200.0000	200.0000	200.0000
200.1000	200.0000	200.0000
	**Standard deviation**	0.03
	**MEAN**	200.01
	**%RSD**	0.02

REVISION HISTORY

REVISED DATE	REVISED BY	CHANGES MADE
6/22/2016	Julie Brunkhorst	Orginal Document

Analytical Method Uncertainty Budget--T2 LCMSMS 2019

Uncertainty Source (Factor)	Estimated Value	Distribution Factor	Type of Uncertainty	Divisor	Method Uncertainty
**Volumetric pipette uncertainty**	+/- 0.1ml	Rectangular	B	1.732	0.06
**Tech pipetting repeatability**	0.20 %RSD	Normal	A	1	0.20
**Repeatability of HPLC system**	10.414 %RSD	Normal	A	1	10.41
**Tech pipetting repeatability 1-5ml**	0.71 %RSD	Normal	A	1	0.71
**Tech pipetting repeatability 200-1000µl**	0.58 %RSD	Normal	A	1	0.58
**Tech pipetting repeatability 20-200µl**	0.54 %RSD	Normal	A	1	0.54
**Tech repeatability 10-100ml dispensor**	0.25 %RSD	Normal	A	1	0.25
**Tech repeatability bench scale**	0.20 %RSD	Normal	A	1	0.20
**Combined Uncertainty**	10.48				
**Expanded Uncertainty**	20.95				

* Combined uncertainty is equal to the root sum square of all variables in the uncertainty budget after being adjusted using distribution factors to standard uncertainty form

**Expanded Uncertainty is equal to the combined uncertainty multiplied by a factor of two to give 95% confidence in the measurement of uncertainty

Calculations of uncertainty (k=2) are based on the criteria outlined in the ISO guide to the expression of uncertainty in measurement (GUM)

	4.9755	4.9532	4.9744
	4.9786	4.9635	4.9687
**Tech pipetting repeatability 30 repetitions using a 5ml ml A class Volumetric pipette**	4.9758	4.9602	4.9790
	4.9772	4.9695	4.9629
	4.9766	4.9504	4.9782
	4.9613	4.9541	4.9826
	4.9532	4.9592	4.9734
	4.9640	4.9794	4.9672
	4.9630	4.9598	4.9686
	4.9850	4.9521	4.9720
		**Standard deviation**	0.01
		**MEAN**	4.97
		**%RSD**	0.20

	107.8	147.39	
	158.43	137.74	146.33
**LCMSMS repeatability 30 repetitions**	129.18	138.22	116.24
	114.14	124.68	117.77
	134.51	144.31	116.01
	144.49	136.68	115.25
	142.98	140.44	111.58
	116.18	151	131.52
	130.92	127.13	111.99
	121.5	123.24	131.37
		**Standard Deviation**	13.65
		**MEAN**	129.97
		**%RSD**	10.500

Tech pipetting repeatability 30 repetitions using a 5ml pipette			
	4968.7000	4885.4000	4950.0000
	4922.1000	4974.5000	4924.8000
	4937.5000	4999.1000	4942.8000
	4929.2000	4971.6000	4954.1000
	4941.1000	4991.1000	4947.3000
	4888.1000	4984.7000	4951.7000
	4911.4000	4967.4000	4938.4000
	4889.6000	4997.7000	4949.0000
	4867.3000	4949.6000	4897.4000
	4894.4000	4975.8000	4933.5000
		**Standard deviation**	35.25
		**MEAN**	4941.18
		**%RSD**	0.71

Tech pipetting repeatability 30 repetitions using a 200-1000µl pipette			
	998.4000	992.3000	1002.4000
	999.6000	997.8000	997.6000
	995.0000	997.9000	999.1000
	993.1000	995.5000	996.8000
	986.2000	989.7000	984.1000
	991.6000	996.5000	1001.2000
	980.5000	991.5000	997.2000
	992.2000	999.1000	997.6000
	991.3000	998.6000	1000.7000
	990.1000	980.2000	998.1000
		**Standard deviation**	5.81
		**MEAN**	994.40
		**%RSD**	0.58

Tech pipetting repeatability 30 repetitions using a 20-200µl pipette			
	199.8000	197.9000	201.5000
	200.1000	198.9000	201.0000
	200.3000	197.2000	201.3000
	199.5000	201.6000	201.2000
	199.6000	199.4000	201.0000
	199.8000	199.3000	201.1000
	200.9000	200.8000	201.2000
	199.0000	199.6000	200.9000
	200.0000	201.2000	201.0000
	199.6000	199.4000	201.1000
		**Standard deviation**	1.09
		**MEAN**	200.17
		**%RSD**	0.54

Tech repeatability 30 repetitions using a 10-100ml dispensor			
	49452.6000	49184.7000	49195.1000
	49432.1000	49130.8000	49273.6000
	49493.0000	49412.6000	49261.8000
	49450.7000	49523.6000	49252.6000
	49373.9000	49405.0000	49115.0000
	49387.7000	49443.8000	49169.4000
	49474.1000	49354.0000	49189.3000
	49357.5000	49443.3000	49135.2000
	49367.6000	49402.4000	49251.1000
	49372.6000	49453.0000	49194.4000
		**Standard deviation**	123.23
		**MEAN**	49331.75
		**%RSD**	0.25

Tech repeatability 30 repetitions using a bench scale S/N 19353802			
	200.0000	200.1000	200.0000
	200.0000	200.0000	200.0000
	200.0000	200.0000	200.0000
	200.1000	200.0000	200.0000
	200.0000	200.0000	200.1000
	200.0000	200.0000	200.0000
	200.0000	200.0000	200.0000
	200.0000	200.0000	200.0000
	200.0000	200.0000	200.0000
	200.1000	200.0000	200.0000
		**Standard deviation**	0.03
		**MEAN**	200.01
		**%RSD**	0.02

REVISION HISTORY

REVISED DATE	REVISED BY	CHANGES MADE
6/22/2016	Julie Brunkhorst	Orginal Document

Analytical Method Uncertainty Budget--Zone LCMSMS

Uncertainty Source (Factor)	Estimated Value	Distribution Factor	Type of Uncertainty	Divisor	Method Uncertainty
**Purity**	99.5 % (+/- 0.5 %)	Rectangular	B	1.732	0.29
**Amount of Crystalline**	+/- 0.1mg	Rectangular	B	1.732	0.06
**Volumetric pipette uncertainty**	+/- 0.1ml	Rectangular	B	1.732	0.06
**Tech pipetting repeatability**	0.20 %RSD	Normal	A	1	0.20
**Repeatability of UV-Spectrophotometer**	0.145 %RSD	Normal	A	1	0.15
**Repeatability of HPLC system**	11.864 %RSD	Normal	A	1	11.86
**Uncertainty of Gold STD (sigma)**	1.0 ug/ml	Rectangular	B	1.732	0.58
**Tech pipetting repeatability 1-5ml**	0.71 %RSD	Normal	A	1	0.71
**Tech pipetting repeatability 200-1000µl**	0.58 %RSD	Normal	A	1	0.58
**Tech pipetting repeatability 20-200µl**	0.54 %RSD	Normal	A	1	0.54
**Tech repeatability 10-100ml dispensor**	0.25 %RSD	Normal	A	1	0.25
**Tech repeatability bench scale**	0.20 %RSD	Normal	A	1	0.20
**Combined Uncertainty**	11.94				
**Expanded Uncertainty**	23.87				

* Combined uncertainty is equal to the root sum square of all variables in the uncertainty budget after being adjusted using distribution factors to standard uncertainty form

**Expanded Uncertainty is equal to the combined uncertainty multiplied by a factor of two to give 95% confidence in the measurement of uncertainty

Calculations of uncertainty (k=2) are based on the criteria outlined in the ISO guide to the expression of uncertainty in measurement (GUM)

Tech pipetting repeatability 30 repetitions using a 5ml ml A class Volumetric pipette

4.9755	4.9532	4.9744
4.9786	4.9635	4.9687
4.9758	4.9602	4.9790
4.9772	4.9695	4.9629
4.9766	4.9504	4.9782
4.9613	4.9541	4.9826
4.9532	4.9592	4.9734
4.9640	4.9794	4.9672
4.9630	4.9598	4.9686
4.9850	4.9521	4.9720
	**Standard deviation**	0.01
	**MEAN**	4.97
	**%RSD**	0.20

UV - Spectrophotometer repeatability 30 repetitions

1.6754	1.6768	1.6795
1.6742	1.6799	1.6795
1.6775	1.6785	1.6821
1.6756	1.6779	1.6813
1.6767	1.6797	1.6802
1.6771	1.6789	1.6817
1.6773	1.6779	1.6802
1.6757	1.6821	1.6820
1.6749	1.6787	1.6771
1.6754	1.6806	1.6830
	**Standard deviation**	0.002
	**MEAN**	1.68
	**%RSD**	0.145

HPLC repeatability 30 repetitions

187.17	181.88	162.99
169.42	147.33	169.47
160.9	173.24	166.01
177.8	193.65	219.94
196.78	210.6	164.78
166	155.92	177.1
139.61	163.25	163.74
139.99	154.16	167.05
167.7	159.2	195.03
172.52	169.2	222.28
	**Standard Deviation**	20.54
	**MEAN**	173.16
	**%RSD**	11.864

Tech pipetting repeatability 30 repetitions using a 5ml pipette			
	4968.7000	4885.4000	4950.0000
	4922.1000	4974.5000	4924.8000
	4937.5000	4999.1000	4942.8000
	4929.2000	4971.6000	4954.1000
	4941.1000	4991.1000	4947.3000
	4888.1000	4984.7000	4951.7000
	4911.4000	4967.4000	4938.4000
	4889.6000	4997.7000	4949.0000
	4867.3000	4949.6000	4897.4000
	4894.4000	4975.8000	4933.5000
		**Standard deviation**	35.25
		**MEAN**	4941.18
		**%RSD**	0.71

Tech pipetting repeatability 30 repetitions using a 200-1000µl pipette			
	998.4000	992.3000	1002.4000
	999.6000	997.8000	997.6000
	995.0000	997.9000	999.1000
	993.1000	995.5000	996.8000
	986.2000	989.7000	984.1000
	991.6000	996.5000	1001.2000
	980.5000	991.5000	997.2000
	992.2000	999.1000	997.6000
	991.3000	998.6000	1000.7000
	990.1000	980.2000	998.1000
		**Standard deviation**	5.81
		**MEAN**	994.40
		**%RSD**	0.58

Tech pipetting repeatability 30 repetitions using a 20-200µl pipette			
	199.8000	197.9000	201.5000
	200.1000	198.9000	201.0000
	200.3000	197.2000	201.3000
	199.5000	201.6000	201.2000
	199.6000	199.4000	201.0000
	199.8000	199.3000	201.1000
	200.9000	200.8000	201.2000
	199.0000	199.6000	200.9000
	200.0000	201.2000	201.0000
	199.6000	199.4000	201.1000
		**Standard deviation**	1.09
		**MEAN**	200.17
		**%RSD**	0.54

Tech repeatability 30 repetitions using a 10-100ml dispensor			
	49452.6000	49184.7000	49195.1000
	49432.1000	49130.8000	49273.6000
	49493.0000	49412.6000	49261.8000
	49450.7000	49523.6000	49252.6000
	49373.9000	49405.0000	49115.0000
	49387.7000	49443.8000	49169.4000
	49474.1000	49354.0000	49189.3000
	49357.5000	49443.3000	49135.2000
	49367.6000	49402.4000	49251.1000
	49372.6000	49453.0000	49194.4000
		**Standard deviation**	123.23
		**MEAN**	49331.75
		**%RSD**	0.25

Tech repeatability 30 repetitions using a bench scale S/N 19353802			
	200.0000	200.1000	200.0000
	200.0000	200.0000	200.0000
	200.0000	200.0000	200.0000
	200.1000	200.0000	200.0000
	200.0000	200.0000	200.1000
	200.0000	200.0000	200.0000
	200.0000	200.0000	200.0000
	200.0000	200.0000	200.0000
	200.0000	200.0000	200.0000
	200.1000	200.0000	200.0000
		**Standard deviation**	0.03
		**MEAN**	200.01
		**%RSD**	0.02

REVISION HISTORY

REVISED DATE	REVISED BY	CHANGES MADE
6/22/2016	Julie Brunkhorst	Orginal Document

Analytical Method Uncertainty Budget--DON LCMSMS 2019

Uncertainty Source (Factor)	Estimated Value	Distribution Factor	Type of Uncertainty	Divisor	Method Uncertainty
**Purity**	99.5 % (+/- 0.5 %)	Rectangular	B	1.732	0.29
**Amount of Crystalline**	+/- 0.1mg	Rectangular	B	1.732	0.06
**Volumetric pipette uncertainty**	+/- 0.1ml	Rectangular	B	1.732	0.06
**Tech pipetting repeatability**	0.20 %RSD	Normal	A	1	0.20
**Repeatability of UV-Spectrophotometer**	0.145 %RSD	Normal	A	1	0.15
**Repeatability of LCMSMS system**	9.204 %RSD	Normal	A	1	9.20
**Uncertainty of Gold STD (sigma)**	1.0 ug/ml	Rectangular	B	1.732	0.58
**Tech pipetting repeatability 1-5ml**	0.71 %RSD	Normal	A	1	0.71
**Tech pipetting repeatability 200-1000µl**	0.58 %RSD	Normal	A	1	0.58
**Tech pipetting repeatability 20-200µl**	0.54 %RSD	Normal	A	1	0.54
**Tech repeatability 10-100ml dispensor**	0.25 %RSD	Normal	A	1	0.25
**Tech repeatability bench scale**	0.20 %RSD	Normal	A	1	0.20
**Combined Uncertainty**	9.30				
**Expanded Uncertainty**	18.59				

* Combined uncertainty is equal to the root sum square of all variables in the uncertainty budget after being adjusted using distribution factors to standard uncertainty form

**Expanded Uncertainty is equal to the combined uncertainty multiplied by a factor of two to give 95% confidence in the measurement of uncertainty

Calculations of uncertainty (k=2) are based on the criteria outlined in the ISO guide to the expression of uncertainty in measurement (GUM)

	4.9755	4.9532	4.9744
	4.9786	4.9635	4.9687
**Tech pipetting repeatability 30 repetitions using a 5ml ml A class Volumetric pipette**	4.9758	4.9602	4.9790
	4.9772	4.9695	4.9629
	4.9766	4.9504	4.9782
	4.9613	4.9541	4.9826
	4.9532	4.9592	4.9734
	4.9640	4.9794	4.9672
	4.9630	4.9598	4.9686
	4.9850	4.9521	4.9720
		**Standard deviation**	0.01
		**MEAN**	4.97
		**%RSD**	0.20

	1.6754	1.6768	1.6795
	1.6742	1.6799	1.6795
**UV - Spectrophotometer repeatability 30 repetitions**	1.6775	1.6785	1.6821
	1.6756	1.6779	1.6813
	1.6767	1.6797	1.6802
	1.6771	1.6789	1.6817
	1.6773	1.6779	1.6802
	1.6757	1.6821	1.6820
	1.6749	1.6787	1.6771
	1.6754	1.6806	1.6830
		**Standard deviation**	0.002
		**MEAN**	1.68
		**%RSD**	0.145

	1.14	0.94	1.07
	0.97	1.08	0.94
**LCMSMS repeatability 30 repetitions**	0.82	1.12	1.1
	1.16	1.08	0.91
	0.83	1.09	0.91
	1.03	1.01	0.89
	0.99	1.12	0.92
	1	1.01	0.96
	1	0.91	0.9
	0.92	0.96	0.97
		**Standard Deviation**	0.09
		**MEAN**	0.99
		**%RSD**	9.204

	4968.7000	4885.4000	4950.0000
	4922.1000	4974.5000	4924.8000
**Tech pipetting repeatability 30 repetitions using a 5ml pipette**	4937.5000	4999.1000	4942.8000
	4929.2000	4971.6000	4954.1000
	4941.1000	4991.1000	4947.3000
	4888.1000	4984.7000	4951.7000
	4911.4000	4967.4000	4938.4000
	4889.6000	4997.7000	4949.0000
	4867.3000	4949.6000	4897.4000
	4894.4000	4975.8000	4933.5000
		**Standard deviation**	35.25
		**MEAN**	4941.18
		**%RSD**	0.71

	998.4000	992.3000	1002.4000
	999.6000	997.8000	997.6000
**Tech pipetting repeatability 30 repetitions using a 200-1000µl pipette**	995.0000	997.9000	999.1000
	993.1000	995.5000	996.8000
	986.2000	989.7000	984.1000
	991.6000	996.5000	1001.2000
	980.5000	991.5000	997.2000
	992.2000	999.1000	997.6000
	991.3000	998.6000	1000.7000
	990.1000	980.2000	998.1000
		**Standard deviation**	5.81
		**MEAN**	994.40
		**%RSD**	0.58

	199.8000	197.9000	201.5000
	200.1000	198.9000	201.0000
**Tech pipetting repeatability 30 repetitions using a 20-200µl pipette**	200.3000	197.2000	201.3000
	199.5000	201.6000	201.2000
	199.6000	199.4000	201.0000
	199.8000	199.3000	201.1000
	200.9000	200.8000	201.2000
	199.0000	199.6000	200.9000
	200.0000	201.2000	201.0000
	199.6000	199.4000	201.1000
		**Standard deviation**	1.09
		**MEAN**	200.17
		**%RSD**	0.54

	49452.6000	49184.7000	49195.1000
	49432.1000	49130.8000	49273.6000
**Tech repeatability 30 repetitions using a 10-100ml dispensor**	49493.0000	49412.6000	49261.8000
	49450.7000	49523.6000	49252.6000
	49373.9000	49405.0000	49115.0000
	49387.7000	49443.8000	49169.4000
	49474.1000	49354.0000	49189.3000
	49357.5000	49443.3000	49135.2000
	49367.6000	49402.4000	49251.1000
	49372.6000	49453.0000	49194.4000
		**Standard deviation**	123.23
		**MEAN**	49331.75
		**%RSD**	0.25

	200.0000	200.1000	200.0000
	200.0000	200.0000	200.0000
**Tech repeatability 30 repetitions using a bench scale S/N 19353802**	200.0000	200.0000	200.0000
	200.1000	200.0000	200.0000
	200.0000	200.0000	200.1000
	200.0000	200.0000	200.0000
	200.0000	200.0000	200.0000
	200.0000	200.0000	200.0000
	200.0000	200.0000	200.0000
	200.1000	200.0000	200.0000
		**Standard deviation**	0.03
		**MEAN**	200.01
		**%RSD**	0.02

REVISION HISTORY

REVISED DATE	REVISED BY	CHANGES MADE
6/22/2016	Julie Brunkhorst	Orginal Document
9/15/2025	Julie Brunkhorst	LCMSMS data was incorrect
