# Supplementary material for: Occurrence and Co-Occurrence of Regulated and Emerging Mycotoxins in Foods Marketed to U.S. Toddlers
Source: Int J Environ Res Public Health. 2026 Jul 23;23(8):949. doi: 10.3390/ijerph23080949 (PMC13512329; doi:10.3390/ijerph23080949)
Supplement: Supplementary file 1 [file ijerph-23-00949-s001.zip › File S3 Toddler Foods Study_PT data_SG_2026.pdf]

Bipea 7-2931-0088 Oats PT2024

**Analytical Criterion**	**Determined Conc. (μg/ml)**	**Assingned Conc. (μg/ml)**	**α PT**
AFB1	1.1	1.21	0.37
AFB2	3.1	2.03	0.61
AFG1	1.6	1.42	0.43
AFG2	2.3	2.14	0.64
OTA	5.5	6.65	2.01
DON	100	94	1.92
3aDON	<100	55	25
15aDON	<100	52	16
NIV	<100	124	33
T2	77	82	27
HT2	137	144	31
ZONE	23	44	25

.

Bipea 3-4431-0150 Sorghum PT2025

**Analytical Criterion**	**Determined Conc. (μg/ml)**	**Assingned Conc. (μg/ml)**	**α PT**
AFB1	2.3	1.86	0.56
AFB2	3	2.43	0.73
AFG1	2.3	1.89	0.57
AFG2	3.1	2.55	0.77
OTA	5.1	6	1.75
DON	125	102	27
3aDON	173	144	38
15aDON	183	168	44
NIV	95	102	27
T2	40	35	11
HT2	27	25	8
ZONE	71	80	22

z-score
-0.3
1.75
0.42
0.25
-0.6
0.24
N/A
N/A
N/A
-0.23
-0.19
-1.68

z-score
0.79
0.78
0.73
0.72
-0.51
0.85
0.77
0.34
-0.26
0.48
0.25
-0.42
